# Exploring the Causal Link Between Autoimmune Diseases and Pulmonary Arterial Hypertension: A Bidirectional Mendelian Randomization Study

**DOI:** 10.5334/gh.1445

**Published:** 2025-07-04

**Authors:** Chao Ma, Cheng Gong, Bin Wang, Yangmeina Li, Yongxiang Qian, Xiaoying Zhang, Dongmei Di, Min Wang

**Affiliations:** 1 Department of Cardiothoracic Surgery, The Third Affiliated Hospital of Soochow University, Changzhou, Jiangsu Province 213003, China; 2 Department of Otolaryngology, The Third Affiliated Hospital of Soochow University, Changzhou, Jiangsu Province 213003, China

**Keywords:** Pulmonary Arterial Hypertension, Autoimmune Diseases, Rheumatoid Arthritis, Inflammatory Bowel Disease, Mendelian Randomization

## Abstract

**Background:**

Pulmonary arterial hypertension (PAH) is a severe vascular disorder with a multifactorial etiology, including potential genetic predispositions. Understanding the causal relationship between autoimmune diseases and the risk of developing PAH can inform clinical strategies for prevention and treatment.

**Methods:**

We conducted a two-sample Mendelian Randomization (MR) analysis to evaluate the causal effect of genetic predisposition to five autoimmune diseases (systemic lupus erythematosus [SLE], rheumatoid arthritis [RA], inflammatory bowel disease [IBD], multiple sclerosis [MS], and type 1 diabetes [T1D]) on the risk of PAH. This involved employing various MR methods (IVW, MR-Egger, Weighted median, Simple mode, and Weighted mode), as well as conducting tests for heterogeneity and horizontal pleiotropy.

**Results:**

The analysis revealed a significant association between genetic predisposition to RA and IBD with an increased risk of PAH (RA: OR = 1.28, 95% CI [1.01–1.61], p = 0.042; IBD: OR = 1.29, 95% CI [1.01–1.64], p = 0.043). However, no association was observed between genetically determined MS, SLE, and T1D with the risk of PAH (MS: p = 0.876; SLE: p = 0.564; T1D: p = 0.061). Additionally, tests for heterogeneity and pleiotropy provided no evidence of their influence, suggesting the robustness of these associations. Reverse MR analysis also indicated no significant effect of PAH on the genetic susceptibility to these autoimmune diseases.

**Conclusion:**

The findings suggest a possible genetic causative link between RA and IBD and the risk of developing PAH. Conversely, genetic predisposition to MS, SLE, and T1D does not appear to influence PAH risk. Understanding these relationships may offer insights into the pathophysiology of PAH and inform screening strategies within at-risk populations.

## Introduction

Pulmonary arterial hypertension (PAH) is a progressive, highly fatal disorder characterized by varying etiologies and an exceptionally poor prognosis, with an estimated three-year survival rate of only 58.2% for patients with idiopathic, familial, or anorexigen-associated PAH (1). According to recent data in Europe, the annual incidence of PAH is estimated at approximately cases per million population, with a reported prevalence ranging from 48 to 55 cases per million population (2). The hallmark pathophysiological changes of PAH consist of constricted and remodeled distal pulmonary vasculature, as well as right ventricular hypertrophy and dilation (3,4). The initiation of PAH is thought to be a confluence of factors such as aberrant angiogenesis, metabolic dysfunctions, DNA damage, genetic mutations, and compromised vascular reactivity (5). Moreover, PAH frequently complicates certain autoimmune diseases, including systemic sclerosis (SSc), systemic lupus erythematosus (SLE), and mixed connective tissue disease, though the exact causal mechanisms remain unclear (6).

Numerous observational studies have reported a connection between autoimmune diseases and PAH (7,8). Notably, 8–12% of SSc patients develop PAH, particularly those with localized skin involvement (9). In the case of SLE, around 5% of patients are affected by PAH, with the presence of anti-RNP and anti-SSA/Ro antibodies, as well as Raynaud’s phenomenon, serving as independent predictive indicators for SLE-associated PAH (10,11). Additionally, PAH presents as a rarer but grave complication in inflammatory bowel disease (IBD) (12), rheumatoid arthritis (RA) (13), and type 1 diabetes (T1D) (14). Autoimmune diseases such as RA and multiple sclerosis (MS) are known for heightened cardiovascular risks, largely attributed to chronic systemic inflammation driven by pro-inflammatory cytokines and autoantibodies, offering a potential explanation for the correlations observed in studies (15). Although randomized controlled trials are deemed the benchmark for establishing causality, their interpretations can be contentious due to potential selection bias and confounders (16).

Mendelian randomization (MR) leverages genetic variations to deduce causal relationships between exposures and outcomes while mitigating the impact of unmeasured confounding (17). Utilizing single nucleotide polymorphisms (SNPs) as instrumental variables, which are genetic variations imparted at conception, MR provides more robust and less confounded estimates (18). Recent advances in genetic studies have identified several key genes associated with PAH, providing valuable insights into disease pathogenesis. The bone morphogenetic protein receptor type 2 (BMPR2) gene remains the most prevalent genetic determinant, with mutations identified in approximately 75% of heritable PAH cases and 26% of idiopathic PAH cases (19). Furthermore, the mutation in T-Box factor 4 (TBX4) account for approximately 5% of pediatric PAH cases and demonstrate distinct clinical phenotypes, including earlier disease onset and more severe pulmonary vascular disease (20,21). The contributions of pharmacological treatments, such as corticosteroids, non-steroidal anti-inflammatory drugs, and biologics, to the manifestation of PAH in autoimmune diseases remain under investigation. This paper endeavors to ascertain the causal associations between PAH and five autoimmune diseases—SLE, RA, IBD, MS, and T1D—employing our MR approach (15). Our research provides novel insights into the complex interplay between autoimmune diseases and PAH, underscoring the importance of genetic underpinnings in disease risk and manifestation. These findings warrant further inquiry into the mechanisms underlying these associations, potentially informing future strategies for prevention and treatment of patients burdened by PAH.

## Methods

### Data sources and study design

**Figure 1:**
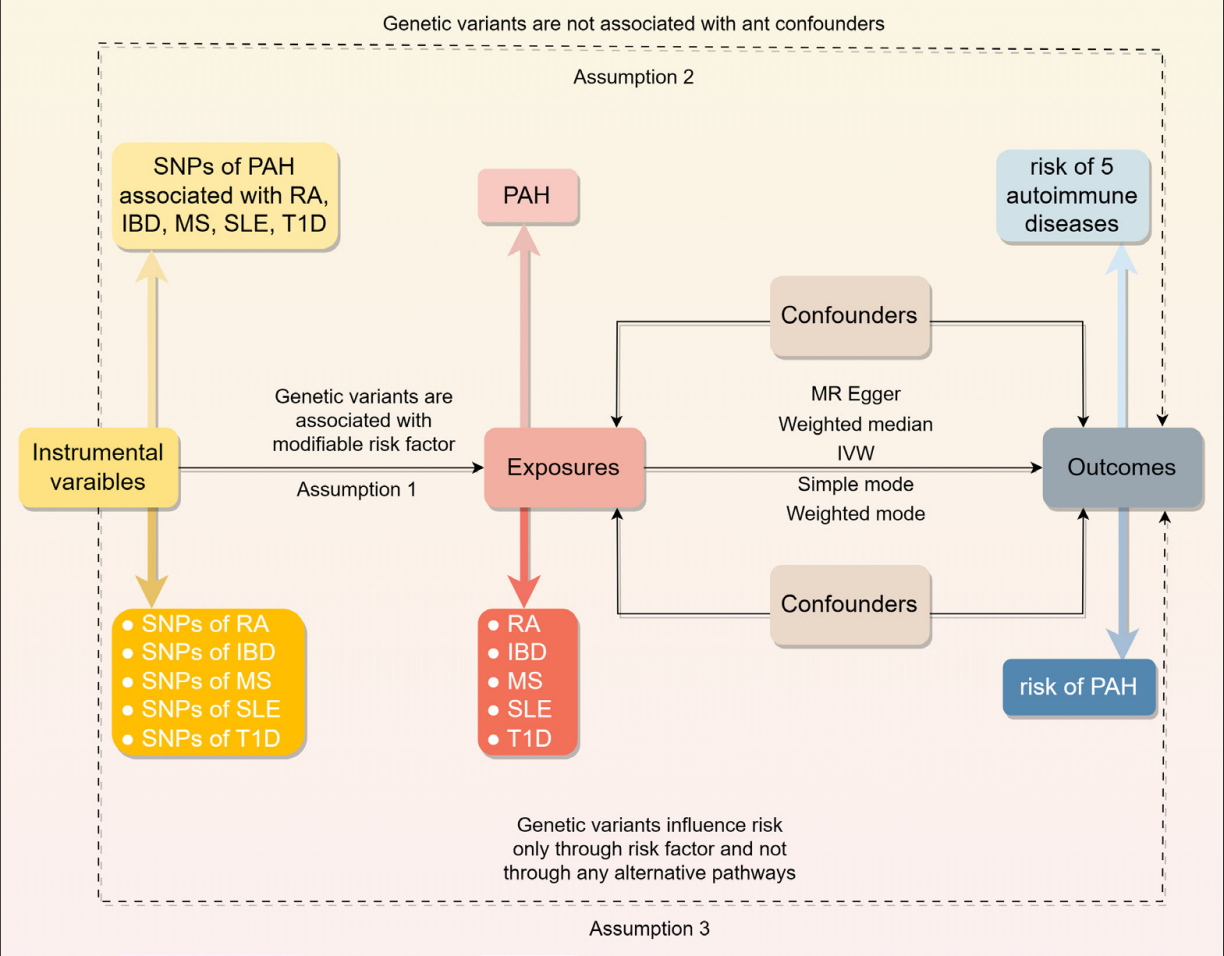
Overview of the study design in this bidirectional MR study.

We employed a two-sample MR design, utilizing a classical MR analysis framework. Figure 1 illustrates the overall study design. Summary-level statistical data for RA were obtained from GWAS meta-analysis comprising 14,361 cases and 42,923 controls. IBD GWAS data were sourced from FinnGen (https://www.finngen.fi/en), including 3,753 cases and 210,300 controls. MS statistics were acquired from the International Multiple Sclerosis Genetics Consortium, encompassing 47,429 MS cases and 68,374 healthy controls of European ancestry. Instrumental variables (IVs) for SLE were derived from GWAS meta-analyses consisting of 5,201 cases and 9,066 controls. T1D data were obtained from a large meta-analysis of 12 European cohorts (phenocode “ebi-a-GCST010681”), including 9,266 cases and 15,574 controls, with a total of 12,783,129 SNPs genotyped. For the outcome dataset, PAH-associated SNPs were retrieved from a public GWAS meta-analysis comprising 125 cases and 162,837 controls of European ancestry. Table 1 summarizes the demographic profiles included in this study.

**Table 1:** Data sources and instrumental variables strength assessment.


TRAITS	PHENOTYPIC CODE	SAMPLE SIZE (CASES/CONTROLS)	ANCESTRY	R^2^(%) (TOTAL)	F (TOTAL)

**Exposures**					

RA	ebi-a-GCST002318	14,361/42,923	European	0.1106	6365

IBD	finn-b-K11_IBD_STRICT	3,753/210,300	European	0.0079	1690

MS	ieu-b-18	47,429/68,374	European	0.0600	6978

SLE	ebi-a-GCST003156	5,201/9,066	European	0.3523	5081

T1D	ebi-a-GCST010681	9,266/15,574	European	0.2969	7486

**Outcomes**					

PAH	finn-b-I9_HYPTENSPUL	125/162,837	European		


F = R^2^(N-K-1)/[K(1-R^2^)], R^2^ = 2 × (1-EAF) × EAF×(b/SD)^2^, SD = SE × N^1/2^, where EAF is the effect allele frequency, b is the estimated effect on adipokines, N is the sample size of the GWAS and SE is the standard error of the estimated effect.

### Two-sample MR

We conducted a two-sample MR analysis to assess the causal relationship between genetic susceptibility to RA, IBD, MS, SLE, T1D, and PAH risk, using SNPs as IVs (15). The MR analysis relies on three fundamental assumptions: (1) IVs must be directly associated with the exposure; (2) IVs must be independent of potential confounders; and (3) IVs should influence the outcome exclusively through the exposure, without alternative pathways. All original studies obtained ethical approval and informed consent. This study was conducted in accordance with the latest STROBE-MR guidelines (29164242).

### Ethical approval

The MR study utilized publicly available GWAS summary statistics, with each contributing GWAS having obtained ethical approval. Summary statistics were accessed through the GWAS Catalog (https://www.ebi.ac.uk). All data are freely available for download and use without restrictions.

### Instrumental variable selection

We considered genetic variants significantly associated with RA, IBD, MS, SLE, and T1D (P < 1 × 10^–5^) as potential IVs (Supplementary Table 1). We assessed linkage disequilibrium (LD) to identify independent SNPs, using a pruning threshold of r² < 0.001 within a 10,000 kb window. We excluded SNPs associated with outcome-related phenotypes, retaining the remaining SNPs for subsequent analysis. We calculated the proportion of variance (R²) explained by the SNPs and the F statistic to evaluate instrument strength (24159078). All IVs in our analyses demonstrated F-statistics > 10, indicating minimal risk of weak instrument bias.

### Statistical analyses

We harmonized aggregated SNP-RA and SNP-PAH statistics to ensure allele consistency between RA and PAH datasets. This adjustment was similarly applied to other exposures (IBD, MS, SLE, and T1D). In the MR analysis, we primarily used the inverse variance weighted (IVW) method, with model selection based on heterogeneity assessment (15). We complemented this approach with weighted median, MR-Egger, simple mode, and weighted mode methods to comprehensively assess causal relationships. These methods operate under different assumptions regarding IV validity: the weighted median provides consistent estimates when at least 50% of IVs are valid, while MR-Egger, despite lower statistical power, offers estimates corrected for pleiotropic effects.

### Pleiotropy and heterogeneity analyses

We implemented multiple sensitivity analyses to ensure robust results. First, we used Cochran’s Q test to assess heterogeneity among individual SNP estimates, guiding the selection of appropriate analysis methods. A p-value > 0.05 indicated no significant heterogeneity, warranting the use of fixed-effects IVW; otherwise, we employed random-effects models. Second, we applied the MR-Egger intercept method to test for horizontal pleiotropy (22), where a significant intercept (p < 0.05) suggested potential bias in IVW estimates. Third, we conducted leave-one-out sensitivity analyses to evaluate the influence of individual SNPs. Fourth, we generated funnel and forest plots to visually assess potential pleiotropy. All analyses were performed using R software (version 4.2.2) with the “TwoSampleMR”, “MR-PRESSO”, and “mr.raps” packages. All p-values were two-sided.

## Results

### Causality of genetic susceptibility to RA and IBD on the risk of PAH

As shown in Table 2, results obtained by the IVW method indicated that RA was related to increased risk of PAH. As observed, the prevalence of PAH in RA cases was 1.275-fold that of the control group (95% Confidence Interval (CI) [1.009–1.610], OR = 1.275, p = 0.042), and we observed that each one unit increase in the IBD resulted in 28.6% higher odds of PAH (95%CI [1.008–1.643], OR = 1.286, p = 0.043). No meaningful results were found in subsequent MR egger, Weighted median, Simple mode and Weighted mode analyses. The linear regression estimates of the five MR methods are detailed in the Supplementary Figure 1. Nevertheless, results analyzed by IVW approach demonstrated that genetic susceptibility to MS, SLE and T1D did not increase the risk of PAH (Respectively, for MS, p = 0.876; for SLE, p = 0.564 and for T1D, p = 0.061). Detailed information was shown in Table 2. Similarly, no meaningful results were found in subsequent MR egger, Weighted median, Simple mode and Weighted mode analyses. The linear regression estimates of the five MR methods are detailed in the Supplementary Figure 2.

**Table 2:** Mendelian randomization estimates of RA, IBD, MS, SLE and T1D on the risk for PAH.


EXPOSURES	METHODS	SNPs (n)	OR	95%CI	*p*-VALUE

RA	MR Egger	109	1.31	0.84–2.06	0.236

	Weighted median	109	1.02	0.71–1.49	0.897

	IVW	109	1.27	1.01–1.61	0.042

	Simple mode	109	0.92	0.37–2.31	0.864

	Weighted mode	109	1.09	0.70–1.70	0.702

IBD	MR Egger	56	1.17	0.64–2.14	0.607

	Weighted median	56	1.14	0.79–1.66	0.484

	IVW	56	1.29	1.01–1.64	0.043

	Simple mode	56	1.11	0.52–2.39	0.789

	Weighted mode	56	1.05	0.56–1.97	0.878

MS	MR Egger	138	1.18	0.88–1.58	0.278

	Weighted median	138	1.07	0.77–1.50	0.675

	IVW	138	0.99	0.83–1.18	0.876

	Simple mode	138	1.11	0.57–2.17	0.751

	Weighted mode	138	1.09	0.78–1.52	0.597

SLE	MR Egger	92	0.93	0.71–1.22	0.601

	Weighted median	92	0.86	0.70–1.05	0.145

	IVW	92	1.04	0.91–1.19	0.564

	Simple mode	92	0.78	0.54–1.13	0.193

	Weighted mode	92	0.84	0.66–1.07	0.164

T1D	MR Egger	175	1.07	0.92–1.23	0.380

	Weighted median	175	0.98	0.83–1.15	0.786

	IVW	175	1.10	1.00–1.21	0.061

	Simple mode	175	1.11	0.76–1.60	0.594

	Weighted mode	175	0.99	0.85–1.15	0.929


### Analysis of horizontal pleiotropy and heterogeneity

In addition to different methods, we performed heterogeneity tests and horizontal pleiotropic analyses as shown in Table 3. In terms of the heterogeneity test, we found weak heterogeneity between RA, IBD, MS, SLE, T1D and PAH. There was no evidence for pleiotropy as detected by MRPRESSO global test, and MR-Egger regression and no other abnormal instrumental variables were measured in the MRPRESSO outlier test. Leave-one-out analysis demonstrated robustness of the effect estimates, suggesting that the potential causal correlation between five autoimmune diseases and PAH risk was not driven by a single SNP (Supplementary Figure 3). The funnel plots (Supplementary Figure 4) and forest plots (Supplementary Figure 5) suggested no significant asymmetry of the MR analyses.

**Table 3:** Pleiotropy and heterogeneity test of the RA, IBD, MS, SLE and T1D IVs from PAH GWAS.


OUTCOMES	EXPOSURE	PLEIOTROPY TEST			HETEROGENEITY TEST						
	
		MR-EGGER			MR-EGGER			INVERSE-VARIANCE WEIGHTED		
		
		INTERCEPT	SE	*P*	Q	Q_-_df	Q_-_*p*val	Q	Q_-_df	Q_-_*p*val

PAH										

	RA	–0.0045	0.0289	0.876	118.1564	107	0.217	118.1835	108	0.237

	IBD	0.0171	0.0520	0.743	42.9250	54	0.861	43.0334	55	0.879

	MS	–0.0326	0.0222	0.144	145.0318	136	0.282	147.3330	137	0.258

	SLE	0.0346	0.0363	0.344	101.7267	90	0.187	102.7517	91	0.188

	T1D	0.0118	0.0220	0.592	154.7772	173	0.837	155.0649	174	0.846


### Reverse MR analysis of the relationship between PAH and five autoimmune diseases

Reverse MR analysis was further performed to evaluate the effect of PAH on the five autoimmune diseases of the forward MR analysis. SNPs met a threshold of p < 5 × 10–8 were selected as genetic instruments strongly associated with PAH, and used for LD clumping. As shown in Table 4, lack of association was obtained between genetic liability to PAH with change of the candidate autoimmune diseases in the IVW analysis, as well as in the MR-Egger, Weighted median, Simple mode and Weighted mode analyses listed in the table (Respectively, for RA, p = 0.310; for IBD, p = 0.513; for MS, p = 0.621; for SLE, p = 0.542 and for T1D, p = 0.262).

**Table 4:** Mendelian randomization results for effect of genetic liability to PAH on RA, IBD, MS, SLE and T1D.


OUTCOMES	METHODS	SNPs (n)	OR	95%CI	*p*-VALUE

RA	MR Egger	9	1.01	0.97–1.06	0.627

	Weighted median	9	1.01	0.98–1.03	0.678

	IVW	9	1.01	0.99–1.03	0.310

	Simple mode	9	1.00	0.97–1.03	0.921

	Weighted mode	9	1.00	0.99–1.03	0.805

IBD	MR Egger	12	1.06	1.00–1.13	0.066

	Weighted median	12	0.99	0.96–1.02	0.665

	IVW	12	0.99	0.97–1.02	0.513

	Simple mode	12	1.00	0.95–1.05	0.996

	Weighted mode	12	1.00	0.96–1.05	0.989

MS	MR Egger	7	1.02	0.96–1.08	0.497

	Weighted median	7	0.99	0.97–1.02	0.669

	IVW	7	0.99	0.97–1.02	0.621

	Simple mode	7	0.98	0.94–1.03	0.549

	Weighted mode	7	0.98	0.94–1.03	0.537

SLE	MR Egger	9	0.96	0.89–1.04	0.347

	Weighted median	9	1.03	0.99–1.07	0.180

	IVW	9	1.01	0.97–1.05	0.542

	Simple mode	9	1.03	0.95–1.11	0.523

	Weighted mode	9	1.03	0.98–1.08	0.252

T1D	MR Egger	12	1.02	0.96–1.08	0.591

	Weighted median	12	1.00	0.97–1.03	0.951

	IVW	12	1.01	0.99–1.04	0.262

	Simple mode	12	1.00	0.95–1.06	0.994

	Weighted mode	12	1.00	0.95–1.05	0.892


## Discussion

The relationship between PAH and autoimmune diseases is an area of growing interest in medical research. Our study contributes novel insights into this interaction with robust MR analyses that reveal the causal effects of RA and IBD on the risk of PAH. These findings are in alignment with observational studies that have suggested associations between specific autoimmune conditions and PAH (22). Our results underscore the increased likelihood of PAH development in patients with RA or IBD, highlighting the need for increased surveillance and early intervention strategies within these populations.

In previous observational research, RA’s complication with PAH was less prevalent compared to SSc or SLE, albeit still significant (23,24). National registries, such as the Korean REOPARD and the UK registry, report low percentages of RA-associated PAH within the context of connective tissue disease-associated PAH (CTD-PH) (13,25). However, associated survival rates after PAH diagnosis in RA patients underscore the condition’s serious implications. Reeves et al.’s use of exercise echocardiography reinforced the notion of RA as a potential risk factor for PAH, with consistent findings across multiple studies highlighting this link (26,27,28). Despite the shared risk of heart failure between RA and general populations, survival outcomes in RA-associated PAH appear to be on par with PAH (29,30). Unexpectedly, our MR findings attribute a causal role to IBD in the development of PAH, diverging from conventional views that implicate IBD medication side effects (31). A specific instance (32) detailed in a Japanese study showed reversible PAH linked to the treatment with natural indigo in ulcerative colitis patients. Additionally, anti-inflammatory medications used in IBD treatment, particularly TNF-α inhibitors, may influence PAH risk through modulation of endothelial function and vascular remodeling (33,34). However, our MR design minimizes confounding by treatment effects as genetic variants are fixed at conception. This outcome emphasizes the association between RA and IBD in PAH, as both independent risk factors and as a consequence of anti-inflammatory drug regimens.

RA encompasses a range of pulmonary manifestations that elevate the overall mortality of affected individuals (35). The pulmonary complications can manifest as direct consequences of the disease or secondary reactions to pharmacological interventions targeting RA, thereby compounding the risk of PAH development (36). Evidence also points to pre-existing venous thrombosis in RA patients as a contributing factor to PAH (37,38). Studies have confirmed that RA can cause deep vein thrombosis and pulmonary artery thrombosis to increase venous thrombosis, with a relative risk of two compared to the control group (39). About a quarter of the RA-PAH population registered in France has deep vein thrombotic PAH (40). Additionally, RA-associated treatments with specific drugs, such as sDMARDS (methotrexate) and certain BDMARDs (rituximab and TNF-α inhibitors), carry a risk of exacerbating PAH (41). The pathogenesis of PAH in connective tissue diseases, including RA, encompasses traditional pathways associated with endothelin-1, nitric oxide, and prostacycline, alongside inflammation and autoimmunity (42). The presence of rheumatoid factor, complement, and immune cells in the lung vasculature of CTD-PAH patients provides further evidence of the autoimmune component (43). Our MR analysis strengthens the causal connection previously established by clinical and pathogenic studies, calling for more in-depth research to unravel the intricacies of this association.

However, our study finds no causal evidence for PAH associated with SSc, SLE, MS, or T1D. This contrasts with traditional observational studies, where factors like therapeutic interventions or confounders could influence results. The pathogenesis of PAH in these conditions remains contentious, despite SSc and SLE being considered primary culprits in the development of PAH within connective tissue diseases (13,24,42,44). Complications such as PAH and interstitial pneumonia serve as significant mortality factors in SSc (45,46). Reports of MS complications with PAH point towards treatments as potential contributors (47,48). Moreover, diabetes-related pathological mechanisms suggest possible contributions to the development of PAH (49), including thickening of the alveolar epithelium and the basal layer of pulmonary capillaries (50), excess production of reactive oxygen species promoted by insulin resistance (51), vasoconstriction response of hyperserotonin (52), antivasodilation and promotion of vascular and myocardial fibrosis (53,54). An interesting study showed that the hemodynamic and histological changes in diabetes were mild, comparable to moderate hypoxia, and only increased PA pressure and RV hypertrophy were seen in diabetes combined with moderate hypoxia, suggesting that diabetic-induced PAH was multifactorial (55). The heterogeneity tests and horizontal pleiotropic analyses strengthened the validity of our findings by showing weak heterogeneity and no evidence of pleiotropy, indicating that the SNP instruments chosen for the study largely meet the assumptions of MR. Furthermore, the reverse MR analysis revealed no association between the genetic susceptibility to PAH and the risk of the autoimmune diseases tested, suggesting a unidirectional relationship where genetic predispositions to certain autoimmune diseases might increase PAH risk, but not vice versa.

Our study’s strength lies in the utilization of extensive datasets, specifically incorporating a large cohort of T1D patients and PAH data from European populations, minimizing heterogeneity and avoiding biases such as reverse causation. The novel contribution of this MR study to the understanding of PAH’s complex etiology across different autoimmune conditions can potentially lead to better diagnostic, preventative, and therapeutic strategies, tailored to the unique clinical presentations that accompany these diseases.

Additionally, epigenetic mechanisms play an important role in the pathogenesis of pulmonary hypertension. Here, we further discuss the potential biological influences of DNA methylation and histone modifications. DNA methylation is a key epigenetic modification that regulates gene expression and is implicated in the pathological processes of PAH. One study has shown that BMPR2 may be silenced due to promoter hypermethylation, contributing to vascular remodeling and cell proliferation (56). Another study further discussed the role of SIN3a (switch-independent 3a), a transcriptional regulator, in the epigenetic mechanisms underlying hypermethylation of BMPR2 in the pathogenesis of PAH (57). These findings suggest that DNA methylation plays a significant role in PAH pathogenesis and may serve as a potential biomarker or therapeutic target. Histone modifications regulate gene expression by altering chromatin structure. Key modifications include acetylation (58), methylation (59), and phosphorylation (60), which are involved in PAH pathology. These mechanisms influence gene expression, thereby affecting cell proliferation, apoptosis, metabolism, and vascular remodeling in the process of PAH.

### Limitation

Some limitations should be noted, such as the modest case numbers, potential lack of representativeness beyond Europeans, and the absence of age and gender considerations in our analysis. Given the lack of corroborative findings in sensitivity analyses, these results should be interpreted prudently, considering the potential for residual confounding and the need for additional studies to confirm these findings. Our study identified several SNP loci with potential associations to PAH, but the analysis lacked granularity in linking these variants to established PAH-associated genes or functional pathways. While the GWAS approach provides broad genetic insights, the mechanistic connections between these SNPs and PAH pathogenesis remain unclear. Future research could benefit from larger sample sizes and a more detailed understanding of the genetic architecture of these diseases to fully elucidate the potential causative pathways that increase the risk of PAH in the context of autoimmune disorders.

## Conclusion

Our study highlights the potential causal relationship between genetic susceptibility to RA and IBD and the increased risk of PAH, which has prospective implications for screening and preventative strategies in these patient populations. However, other autoimmune diseases, including SLE, MS, and T1D, did not display a causal relationship with PAH in this study, indicating that drug-related and additional factors may underlie the link between these autoimmune conditions and PAH. Our findings reinforce the need for further examination and emphasize the importance of precision in assessing risk and managing PAH within the context of autoimmune diseases.

## Additional Files

The additional files for this article can be found as follows:

10.5334/gh.1445.s1 Supplementary Table 1. SNPs used to analyze the causal relationship between five autoimmune diseases and PAH.

10.5334/gh.1445.s2 Supplementary Figure 1. Mendelian randomization analysis of autoimmune diseases and the risk of PAH. (A)RA to PAH; (B) IBD to PAH.

10.5334/gh.1445.s3 Supplementary Figure 2. Mendelian randomization analysis of autoimmune diseases and the risk of PAH. (A) MS to PAH; (B)SLE to PAH; (C)T1D to PAH.

10.5334/gh.1445.s4 Supplementary Figure 3. The MR “leave-one-out” sensitivity analysis of five autoimmune diseases and PAH. (A)RA to PAH; (B) IBD to PAH; (C) MS to PAH; (D)SLE to PAH; (E)T1D to PAH.

10.5334/gh.1445.s5 Supplementary Figure 4. Funnel plots of five autoimmune diseases with PAH. The X-axis represents odds ratio (OR), and the Y-axis represents standard error (SE). (A)RA to PAH; (B) IBD to PAH; (C) MS to PAH; (D)SLE to PAH; (E)T1D to PAH.

10.5334/gh.1445.s6 Supplementary Figure 5. Forest plots of five autoimmune diseases with PAH. (A)RA to PAH; (B) IBD to PAH; (C) MS to PAH; (D)SLE to PAH; (E)T1D to PAH.
