# Supplementary material for: Exploring the Causal Link Between Autoimmune Diseases and Pulmonary Arterial Hypertension: A Bidirectional Mendelian Randomization Study
Source: Glob Heart. 2025 Jul 4;20(1):58. doi: 10.5334/gh.1445 (PMC12227091; doi:10.5334/gh.1445)
Supplement: Supplementary Table 1. — SNPs used to analyze the causal relationship between five autoimmune diseases and PAH. [file gh-20-1-1445-s1.pdf]

Supplementary Table 1

SNP	effect_allele	other_allele	effect_allele	other_allele	beta.exposu	beta.outcon	eaf.exposure	eaf.outcome
rs10012656	A	G	A	G	-0.094311	-0.0459	0.502	0.6241
rs10175798	A	G	A	G	0.0861777	-0.0368	0.6203	0.5713
rs1039548	C	T	C	T	-0.067659	-0.3096	0.7505	0.723
rs10774624	A	G	A	G	-0.083382	-0.321	0.5298	0.5996
rs10798056	G	A	G	A	0.0943107	0.0514	0.829	0.7724
rs10842364	A	G	A	G	0.0953102	0.0085	0.5666	0.516
rs10919605	T	C	T	C	-0.061875	-0.1207	0.4105	0.4063
rs10958974	T	C	T	C	0.0676586	0.0179	0.506	0.5042
rs11088320	T	C	T	C	-0.072571	-0.1234	0.2952	0.2935
rs11089637	C	T	C	T	0.0833816	-0.0052	0.167	0.2659
rs11102660	G	A	G	A	-0.09531	-0.0186	0.173	0.1779
rs11148065	C	T	C	T	0.430783	-0.1265	0.0139	0.00501
rs11217044	C	T	C	T	-0.131028	-0.3825	0.2028	0.2257
rs11574914	A	G	A	G	0.113329	-0.1337	0.3151	0.2799
rs11678056	C	T	C	T	0.0943107	0.0773	0.1302	0.1264
rs11757863	T	C	T	C	-0.18633	-0.6286	0.0417	0.02197
rs11889341	T	C	T	C	0.131028	-0.0035	0.2286	0.2316
rs11933540	C	T	C	T	0.139262	0.1845	0.3221	0.3098
rs12126142	A	G	A	G	-0.083382	0.341	0.3598	0.2977
rs12137270	T	C	T	C	0.0861777	-0.0148	0.2614	0.247
rs12466919	T	C	T	C	0.113329	-0.0394	0.3618	0.3449
rs12574838	G	A	G	A	0.116534	-0.2269	0.8708	0.8706
rs12887885	T	G	T	G	0.076961	0.1221	0.2008	0.2498
rs13097127	T	C	T	C	-0.072571	-0.2151	0.4125	0.4679
rs13142500	C	T	C	T	0.0943107	0.057	0.493	0.4903
rs13191810	T	C	T	C	-0.261365	0.0329	0.3191	0.2814
rs1424813	C	T	C	T	-0.122218	0.1397	0.8976	0.9265
rs1516971	C	T	C	T	-0.113329	0.2059	0.1054	0.1165
rs1571878	T	C	T	C	-0.150823	-0.0621	0.5785	0.5537
rs1633360	T	C	T	C	0.0676586	0.2476	0.6024	0.5512
rs1813375	T	G	T	G	0.0582689	0.036	0.4871	0.4258
rs1858036	G	A	G	A	-0.113329	-0.1749	0.339	0.2926
rs18778617	A	G	A	G	-0.116534	0.26	0.3121	0.3686
rs1893592	C	A	C	A	-0.10436	-0.1161	0.2763	0.3166
rs1953126	C	T	C	T	-0.086178	-0.014	0.6402	0.5642
rs1954404	T	C	T	C	-0.072571	-0.1551	0.4712	0.445
rs2105325	C	A	C	A	0.105361	0.1757	0.7545	0.802
rs212389	A	G	A	G	0.0953102	-0.2019	0.6262	0.7153
rs2201584	A	G	A	G	-0.083382	-0.1715	0.1581	0.1369
rs2233424	T	C	T	C	0.231112	-0.3786	0.0497	0.06554
rs2235924	A	G	A	G	-0.094311	0.0608	0.3131	0.3904
rs2301888	A	G	A	G	-0.127833	0.037	0.3608	0.3305
rs2317230	T	G	T	G	0.076961	0.2103	0.4622	0.4111
rs2421206	G	T	G	T	-0.086178	0.0024	0.339	0.2908
rs2422408	C	T	C	T	-0.076961	-0.1063	0.6074	0.556
rs2476601	G	A	G	A	-0.593327	-0.5763	0.9056	0.8518

rs2504235	G	A	G	A	0.0833816	0.1355	0.5944	0.6138
rs2510066	T	C	T	C	-0.061875	0.0863	0.3718	0.4187
rs2561477	A	G	A	G	-0.083382	0.0753	0.3022	0.3151
rs2671692	A	G	A	G	0.076961	-0.0107	0.6044	0.6169
rs2736337	C	T	C	T	0.105361	0.211	0.2445	0.2583
rs28411352	T	C	T	C	0.113329	0.0039	0.2505	0.2919
rs2858329	G	A	G	A	0.446287	-0.0614	0.5139	0.4349
rs3087243	A	G	A	G	-0.139262	-0.0061	0.4702	0.33
rs35086414	C	A	C	A	-0.086178	-0.2678	0.1978	0.1806
rs3778753	G	A	G	A	0.105361	0.0504	0.4751	0.4409
rs3784099	A	G	A	G	-0.094311	-0.0462	0.3439	0.3058
rs3806624	G	A	G	A	0.0833816	-0.1167	0.4672	0.4769
rs3824660	T	C	T	C	-0.072571	-0.1034	0.6044	0.5763
rs4094864	A	G	A	G	-0.061875	-0.1195	0.4901	0.5292
rs41264261	T	C	T	C	-0.328504	-0.445	0.0288	0.03551
rs4239702	C	T	C	T	0.116534	-0.1287	0.7127	0.7126
rs4272	G	A	G	A	0.0943107	-0.1641	0.2237	0.2218
rs4409785	C	T	C	T	0.105361	0.2898	0.1899	0.1671
rs4616297	A	G	A	G	0.0861777	-0.0195	0.2783	0.2594
rs4780401	T	G	T	G	0.0676586	-0.0424	0.5934	0.6104
rs4938534	A	G	A	G	0.076961	0.1736	0.6402	0.6348
rs5019428	A	G	A	G	0.0861777	0.1436	0.5229	0.6087
rs56262258	C	A	C	A	0.0943107	-0.2524	0.1809	0.1336
rs59716545	G	T	G	T	0.0943107	-0.0798	0.4702	0.5159
rs60726493	T	C	T	C	0.076961	-0.0192	0.329	0.3266
rs62321692	C	A	C	A	0.127833	-0.3233	0.0835	0.08474
rs62442789	A	C	A	C	-0.094311	0.2097	0.171	0.2
rs6442323	G	A	G	A	-0.076961	0.0387	0.4861	0.4952
rs6561043	A	G	A	G	-0.072571	-0.2176	0.7654	0.7925
rs6712515	C	T	C	T	-0.10436	-0.1176	0.5408	0.5399
rs67250450	C	T	C	T	-0.086178	-0.0839	0.2256	0.1801
rs6732565	G	A	G	A	-0.076961	0.0947	0.3757	0.4008
rs678347	A	G	A	G	-0.072571	-0.0537	0.7525	0.7928
rs6930468	G	A	G	A	0.0943107	-0.2188	0.6501	0.6252
rs6986109	T	G	T	G	-0.083382	-0.0077	0.7008	0.7535
rs706778	T	C	T	C	0.0861777	-0.053	0.4135	0.5237
rs71508903	T	C	T	C	0.157004	-0.0511	0.2197	0.1862
rs72634030	A	C	A	C	0.113329	0.3935	0.0915	0.131
rs73000522	T	C	T	C	-0.18633	-0.02	0.0885	0.1392
rs73013527	T	C	T	C	-0.094311	0.0477	0.5298	0.5586
rs73081554	T	C	T	C	0.165514	-0.0207	0.0517	0.06063
rs74433766	A	G	A	G	-0.150823	0.4743	0.0229	0.01721
rs74984480	T	C	T	C	0.139762	0.1547	0.0646	0.08267
rs75525553	G	A	G	A	0.198451	0.2589	0.0268	0.03318
rs76153210	T	C	T	C	0.173953	-0.697	0.0159	0.01178
rs773125	G	A	G	A	-0.086178	-0.0674	0.3887	0.4209
rs7731626	A	G	A	G	-0.18633	0.0683	0.3777	0.2787

rs7752903	G	T	G	T	0.328504	-0.0573	0.0199	0.01783
rs793108	T	C	T	C	0.0676586	-0.0867	0.4543	0.5755
rs79893749	T	C	T	C	-0.116534	0.1512	0.1372	0.14
rs8026898	A	G	A	G	0.14842	-0.0171	0.2853	0.283
rs8032939	C	T	C	T	0.116534	0.1742	0.2416	0.2805
rs8065686	T	C	T	C	0.113329	-0.1999	0.0765	0.04081
rs8083786	G	A	G	A	0.127833	0.0642	0.1362	0.1716
rs8126756	C	T	C	T	-0.086178	-0.0818	0.1421	0.1836
rs9258276	T	C	T	C	-0.235722	0.2588	0.0249	0.01572
rs9261602	G	A	G	A	0.116534	-0.2124	0.5477	0.4951
rs9277411	T	C	T	C	-0.287682	-0.2524	0.3131	0.2564
rs9372120	G	T	G	T	0.0943107	-0.0495	0.1938	0.2349
rs947474	A	G	A	G	0.10436	-0.0694	0.8211	0.8188
rs9603616	T	C	T	C	-0.105361	0.2398	0.338	0.3424
rs968567	T	C	T	C	-0.105361	0.0784	0.1501	0.1086
rs9826828	A	G	A	G	0.350657	1.3026	0.0189	0.001916

remove	palindromic	ambiguous	id.outcome	chr	pos	se.outcome	samplesize.c	pval.outcom
FALSE	FALSE	FALSE	finn-b-I9_HY	4	80940592	0.131		0.726001
FALSE	FALSE	FALSE	finn-b-I9_HY	2	30449594	0.1275		0.7732
FALSE	FALSE	FALSE	finn-b-I9_HY	14	83734935	0.1413		0.0284302
FALSE	FALSE	FALSE	finn-b-I9_HY	12	111833788	0.1305		0.0139101
FALSE	FALSE	FALSE	finn-b-I9_HY	1	186711910	0.1515		0.7345
FALSE	FALSE	FALSE	finn-b-I9_HY	12	24678572	0.1275		0.9472
FALSE	FALSE	FALSE	finn-b-I9_HY	1	198898427	0.1296		0.3517
FALSE	FALSE	FALSE	finn-b-I9_HY	9	10249906	0.1266		0.8879
FALSE	FALSE	FALSE	finn-b-I9_HY	21	36815090	0.1395		0.3765
FALSE	FALSE	FALSE	finn-b-I9_HY	22	21979096	0.1436		0.9711
FALSE	FALSE	FALSE	finn-b-I9_HY	1	114150717	0.1671		0.9113
FALSE	FALSE	FALSE	finn-b-I9_HY	4	27782743	0.9405		0.893
FALSE	FALSE	FALSE	finn-b-I9_HY	11	118696022	0.1522		0.0119801
FALSE	FALSE	FALSE	finn-b-I9_HY	9	34710338	0.1421		0.3469
FALSE	FALSE	FALSE	finn-b-I9_HY	2	62460013	0.1906		0.685001
FALSE	FALSE	FALSE	finn-b-I9_HY	6	27228184	0.4471		0.1597
FALSE	FALSE	FALSE	finn-b-I9_HY	2	191943742	0.1506		0.9813
FALSE	FALSE	FALSE	finn-b-I9_HY	4	26120001	0.1371		0.1785
FALSE	FALSE	FALSE	finn-b-I9_HY	1	154425456	0.1393		0.01434
FALSE	FALSE	FALSE	finn-b-I9_HY	1	117264336	0.1471		0.9197
FALSE	FALSE	FALSE	finn-b-I9_HY	2	61092858	0.134		0.7687
FALSE	FALSE	FALSE	finn-b-I9_HY	11	36533885	0.1881		0.2278
FALSE	FALSE	FALSE	finn-b-I9_HY	14	35376082	0.1467		0.4049
FALSE	FALSE	FALSE	finn-b-I9_HY	3	192752605	0.1269		0.0899891
FALSE	FALSE	FALSE	finn-b-I9_HY	4	10727357	0.1272		0.654201
FALSE	FALSE	FALSE	finn-b-I9_HY	6	32586831	0.1839		0.8578
FALSE	FALSE	FALSE	finn-b-I9_HY	3	189225809	0.2471		0.5719
FALSE	FALSE	FALSE	finn-b-I9_HY	8	129542100	0.1957		0.2927
FALSE	FALSE	FALSE	finn-b-I9_HY	6	167540842	0.1278		0.6271
FALSE	FALSE	FALSE	finn-b-I9_HY	12	58108052	0.1281		0.0531998
FALSE	FALSE	FALSE	finn-b-I9_HY	3	28078571	0.1285		0.7795
FALSE	FALSE	FALSE	finn-b-I9_HY	2	65598241	0.14		0.2117
FALSE	FALSE	FALSE	finn-b-I9_HY	1	2525665	0.1312		0.0474602
FALSE	FALSE	FALSE	finn-b-I9_HY	21	43855067	0.1361		0.3936
FALSE	FALSE	FALSE	finn-b-I9_HY	9	123640500	0.1279		0.9126
FALSE	FALSE	FALSE	finn-b-I9_HY	10	111502338	0.1274		0.2233
FALSE	FALSE	FALSE	finn-b-I9_HY	1	173349725	0.1598		0.2715
FALSE	FALSE	FALSE	finn-b-I9_HY	6	159489791	0.1396		0.1483
FALSE	FALSE	FALSE	finn-b-I9_HY	1	67787715	0.1877		0.361
FALSE	FALSE	FALSE	finn-b-I9_HY	6	44233921	0.252		0.133
FALSE	FALSE	FALSE	finn-b-I9_HY	1	17415655	0.1298		0.639699
FALSE	FALSE	FALSE	finn-b-I9_HY	1	17672730	0.1345		0.7831
FALSE	FALSE	FALSE	finn-b-I9_HY	1	157674997	0.1295		0.1044
FALSE	FALSE	FALSE	finn-b-I9_HY	19	11262477	0.1413		0.9866
FALSE	FALSE	FALSE	finn-b-I9_HY	3	171677350	0.1271		0.403
FALSE	FALSE	FALSE	finn-b-I9_HY	1	114377568	0.1793		0.001308

FALSE	FALSE	FALSE	finn-b-I9_HY	13	28612886	0.1303	0.2982
FALSE	FALSE	FALSE	finn-b-I9_HY	11	64052447	0.1289	0.503
FALSE	FALSE	FALSE	finn-b-I9_HY	5	102608924	0.1362	0.5805
FALSE	FALSE	FALSE	finn-b-I9_HY	10	50097819	0.1305	0.9347
FALSE	FALSE	FALSE	finn-b-I9_HY	8	11341880	0.1598	0.1869
FALSE	FALSE	FALSE	finn-b-I9_HY	1	38278579	0.1392	0.9775
FALSE	FALSE	FALSE	finn-b-I9_HY	6	32665079	0.1295	0.6356
FALSE	FALSE	FALSE	finn-b-I9_HY	2	204738919	0.135	0.9642
FALSE	FALSE	FALSE	finn-b-I9_HY	1	197800965	0.1647	0.1039
FALSE	FALSE	FALSE	finn-b-I9_HY	7	128580042	0.1277	0.6934
FALSE	FALSE	FALSE	finn-b-I9_HY	14	68749927	0.1371	0.7362
FALSE	FALSE	FALSE	finn-b-I9_HY	3	27764623	0.1269	0.3581
FALSE	FALSE	FALSE	finn-b-I9_HY	10	8104722	0.1285	0.4211
FALSE	FALSE	FALSE	finn-b-I9_HY	18	67545498	0.1272	0.3475
FALSE	FALSE	FALSE	finn-b-I9_HY	1	203743798	0.3469	0.1995
FALSE	FALSE	FALSE	finn-b-I9_HY	20	44749251	0.1403	0.3592
FALSE	FALSE	FALSE	finn-b-I9_HY	7	92236829	0.1531	0.2838
FALSE	FALSE	FALSE	finn-b-I9_HY	11	95311422	0.17	0.0882999
FALSE	FALSE	FALSE	finn-b-I9_HY	16	30730679	0.1465	0.8943
FALSE	FALSE	FALSE	finn-b-I9_HY	16	11839326	0.1305	0.745101
FALSE	FALSE	FALSE	finn-b-I9_HY	11	111275133	0.1321	0.1887
FALSE	FALSE	FALSE	finn-b-I9_HY	3	17046866	0.1301	0.2696
FALSE	FALSE	FALSE	finn-b-I9_HY	2	204605029	0.1859	0.1746
FALSE	FALSE	FALSE	finn-b-I9_HY	17	38032680	0.1266	0.5284
FALSE	FALSE	FALSE	finn-b-I9_HY	15	91011262	0.1354	0.8871
FALSE	FALSE	FALSE	finn-b-I9_HY	4	123261530	0.2257	0.152
FALSE	FALSE	FALSE	finn-b-I9_HY	6	149823896	0.1568	0.1812
FALSE	FALSE	FALSE	finn-b-I9_HY	3	12722870	0.1276	0.7618
FALSE	FALSE	FALSE	finn-b-I9_HY	13	42997574	0.1559	0.1629
FALSE	FALSE	FALSE	finn-b-I9_HY	2	100806514	0.1274	0.356
FALSE	FALSE	FALSE	finn-b-I9_HY	7	28174986	0.1646	0.6105
FALSE	FALSE	FALSE	finn-b-I9_HY	2	111607832	0.1292	0.4639
FALSE	FALSE	FALSE	finn-b-I9_HY	8	102463602	0.1581	0.734199
FALSE	FALSE	FALSE	finn-b-I9_HY	6	426268	0.1314	0.0959489
FALSE	FALSE	FALSE	finn-b-I9_HY	8	81286298	0.1479	0.9587
FALSE	FALSE	FALSE	finn-b-I9_HY	10	6098949	0.1272	0.677001
FALSE	FALSE	FALSE	finn-b-I9_HY	10	63779871	0.1629	0.7536
FALSE	FALSE	FALSE	finn-b-I9_HY	17	5272580	0.1851	0.0335598
FALSE	FALSE	FALSE	finn-b-I9_HY	11	107966040	0.1818	0.9125
FALSE	FALSE	FALSE	finn-b-I9_HY	11	128496952	0.1288	0.7112
FALSE	FALSE	FALSE	finn-b-I9_HY	3	58302935	0.2694	0.9389
FALSE	FALSE	FALSE	finn-b-I9_HY	3	24889399	0.4989	0.3418
FALSE	FALSE	FALSE	finn-b-I9_HY	6	14103212	0.2304	0.5018
FALSE	FALSE	FALSE	finn-b-I9_HY	6	138008508	0.3593	0.4711
FALSE	FALSE	FALSE	finn-b-I9_HY	6	44284508	0.5916	0.2387
FALSE	FALSE	FALSE	finn-b-I9_HY	12	56401085	0.1282	0.5991
FALSE	FALSE	FALSE	finn-b-I9_HY	5	55444683	0.1414	0.629001

FALSE	FALSE	FALSE	finn-b-I9_HY	6	138227364	0.4896	0.9069
FALSE	FALSE	FALSE	finn-b-I9_HY	10	31415106	0.1284	0.499401
FALSE	FALSE	FALSE	finn-b-I9_HY	3	46253650	0.1838	0.4107
FALSE	FALSE	FALSE	finn-b-I9_HY	15	69991417	0.1408	0.9031
FALSE	FALSE	FALSE	finn-b-I9_HY	15	38834033	0.141	0.2167
FALSE	FALSE	FALSE	finn-b-I9_HY	17	42193897	0.3255	0.5391
FALSE	FALSE	FALSE	finn-b-I9_HY	18	12881361	0.1682	0.7027
FALSE	FALSE	FALSE	finn-b-I9_HY	21	34775444	0.1638	0.6173
FALSE	FALSE	FALSE	finn-b-I9_HY	6	29731791	0.5064	0.609399
FALSE	FALSE	FALSE	finn-b-I9_HY	2	76934183	0.1266	0.09356
FALSE	FALSE	FALSE	finn-b-I9_HY	6	33051683	0.1511	0.0948091
FALSE	FALSE	FALSE	finn-b-I9_HY	6	106667535	0.1499	0.7411
FALSE	FALSE	FALSE	finn-b-I9_HY	10	6390450	0.1646	0.6732
FALSE	FALSE	FALSE	finn-b-I9_HY	13	40368069	0.1342	0.0738397
FALSE	FALSE	FALSE	finn-b-I9_HY	11	61595564	0.2071	0.705
FALSE	FALSE	FALSE	finn-b-I9_HY	3	136402060	1.5268	0.3936

outcome	originalnam	outcome.de	mr_keep.ou	data_source	proxy.outco	target_snp.c	proxy_snp.o	target_a1.ou
Hypertensio	Hypertensio	Hypertensio	TRUE	igd				
Hypertensio	Hypertensio	Hypertensio	TRUE	igd				
Hypertensio	Hypertensio	Hypertensio	TRUE	igd				
Hypertensio	Hypertensio	Hypertensio	TRUE	igd				
Hypertensio	Hypertensio	Hypertensio	TRUE	igd				
Hypertensio	Hypertensio	Hypertensio	TRUE	igd				
Hypertensio	Hypertensio	Hypertensio	TRUE	igd				
Hypertensio	Hypertensio	Hypertensio	TRUE	igd				
Hypertensio	Hypertensio	Hypertensio	TRUE	igd				
Hypertensio	Hypertensio	Hypertensio	TRUE	igd				
Hypertensio	Hypertensio	Hypertensio	TRUE	igd				
Hypertensio	Hypertensio	Hypertensio	TRUE	igd				
Hypertensio	Hypertensio	Hypertensio	TRUE	igd				
Hypertensio	Hypertensio	Hypertensio	TRUE	igd				
Hypertensio	Hypertensio	Hypertensio	TRUE	igd				
Hypertensio	Hypertensio	Hypertensio	TRUE	igd				
Hypertensio	Hypertensio	Hypertensio	TRUE	igd				
Hypertensio	Hypertensio	Hypertensio	TRUE	igd				
Hypertensio	Hypertensio	Hypertensio	TRUE	igd				
Hypertensio	Hypertensio	Hypertensio	TRUE	igd				
Hypertensio	Hypertensio	Hypertensio	TRUE	igd				
Hypertensio	Hypertensio	Hypertensio	TRUE	igd	TRUE	rs12574838	rs5030409	A
Hypertensio	Hypertensio	Hypertensio	TRUE	igd				
Hypertensio	Hypertensio	Hypertensio	TRUE	igd				
Hypertensio	Hypertensio	Hypertensio	TRUE	igd				
Hypertensio	Hypertensio	Hypertensio	TRUE	igd				
Hypertensio	Hypertensio	Hypertensio	TRUE	igd				
Hypertensio	Hypertensio	Hypertensio	TRUE	igd				
Hypertensio	Hypertensio	Hypertensio	TRUE	igd				
Hypertensio	Hypertensio	Hypertensio	TRUE	igd				
Hypertensio	Hypertensio	Hypertensio	TRUE	igd				
Hypertensio	Hypertensio	Hypertensio	TRUE	igd				
Hypertensio	Hypertensio	Hypertensio	TRUE	igd	TRUE	rs18778617	rs3748817	A
Hypertensio	Hypertensio	Hypertensio	TRUE	igd				
Hypertensio	Hypertensio	Hypertensio	TRUE	igd				
Hypertensio	Hypertensio	Hypertensio	TRUE	igd				
Hypertensio	Hypertensio	Hypertensio	TRUE	igd				
Hypertensio	Hypertensio	Hypertensio	TRUE	igd				
Hypertensio	Hypertensio	Hypertensio	TRUE	igd				
Hypertensio	Hypertensio	Hypertensio	TRUE	igd				
Hypertensio	Hypertensio	Hypertensio	TRUE	igd				
Hypertensio	Hypertensio	Hypertensio	TRUE	igd				
Hypertensio	Hypertensio	Hypertensio	TRUE	igd				
Hypertensio	Hypertensio	Hypertensio	TRUE	igd				
Hypertensio	Hypertensio	Hypertensio	TRUE	igd				
Hypertensio	Hypertensio	Hypertensio	TRUE	igd				

Hypertensio	Hypertensio	Hypertensio	TRUE	igd					
Hypertensio	Hypertensio	Hypertensio	TRUE	igd					
Hypertensio	Hypertensio	Hypertensio	TRUE	igd					
Hypertensio	Hypertensio	Hypertensio	TRUE	igd					
Hypertensio	Hypertensio	Hypertensio	TRUE	igd					
Hypertensio	Hypertensio	Hypertensio	TRUE	igd					
Hypertensio	Hypertensio	Hypertensio	TRUE	igd		TRUE	rs2858329	rs2856667	A
Hypertensio	Hypertensio	Hypertensio	TRUE	igd					
Hypertensio	Hypertensio	Hypertensio	TRUE	igd					
Hypertensio	Hypertensio	Hypertensio	TRUE	igd					
Hypertensio	Hypertensio	Hypertensio	TRUE	igd					
Hypertensio	Hypertensio	Hypertensio	TRUE	igd					
Hypertensio	Hypertensio	Hypertensio	TRUE	igd					
Hypertensio	Hypertensio	Hypertensio	TRUE	igd					
Hypertensio	Hypertensio	Hypertensio	TRUE	igd					
Hypertensio	Hypertensio	Hypertensio	TRUE	igd					
Hypertensio	Hypertensio	Hypertensio	TRUE	igd					
Hypertensio	Hypertensio	Hypertensio	TRUE	igd					
Hypertensio	Hypertensio	Hypertensio	TRUE	igd					
Hypertensio	Hypertensio	Hypertensio	TRUE	igd					
Hypertensio	Hypertensio	Hypertensio	TRUE	igd					
Hypertensio	Hypertensio	Hypertensio	TRUE	igd					
Hypertensio	Hypertensio	Hypertensio	TRUE	igd		TRUE	rs56262258	rs62183988	C
Hypertensio	Hypertensio	Hypertensio	TRUE	igd		TRUE	rs59716545	rs35736272	G
Hypertensio	Hypertensio	Hypertensio	TRUE	igd		TRUE	rs60726493	rs2238325	T
Hypertensio	Hypertensio	Hypertensio	TRUE	igd					
Hypertensio	Hypertensio	Hypertensio	TRUE	igd					
Hypertensio	Hypertensio	Hypertensio	TRUE	igd					
Hypertensio	Hypertensio	Hypertensio	TRUE	igd					
Hypertensio	Hypertensio	Hypertensio	TRUE	igd					
Hypertensio	Hypertensio	Hypertensio	TRUE	igd					
Hypertensio	Hypertensio	Hypertensio	TRUE	igd					
Hypertensio	Hypertensio	Hypertensio	TRUE	igd					
Hypertensio	Hypertensio	Hypertensio	TRUE	igd					
Hypertensio	Hypertensio	Hypertensio	TRUE	igd					
Hypertensio	Hypertensio	Hypertensio	TRUE	igd					
Hypertensio	Hypertensio	Hypertensio	TRUE	igd					
Hypertensio	Hypertensio	Hypertensio	TRUE	igd					
Hypertensio	Hypertensio	Hypertensio	TRUE	igd					
Hypertensio	Hypertensio	Hypertensio	TRUE	igd					
Hypertensio	Hypertensio	Hypertensio	TRUE	igd					
Hypertensio	Hypertensio	Hypertensio	TRUE	igd					
Hypertensio	Hypertensio	Hypertensio	TRUE	igd					
Hypertensio	Hypertensio	Hypertensio	TRUE	igd					
Hypertensio	Hypertensio	Hypertensio	TRUE	igd					
Hypertensio	Hypertensio	Hypertensio	TRUE	igd		TRUE	rs773125	rs10876864	G
Hypertensio	Hypertensio	Hypertensio	TRUE	igd					

Hypertensio	Hypertensio	Hypertensio	TRUE	igd
Hypertensio	Hypertensio	Hypertensio	TRUE	igd
Hypertensio	Hypertensio	Hypertensio	TRUE	igd
Hypertensio	Hypertensio	Hypertensio	TRUE	igd
Hypertensio	Hypertensio	Hypertensio	TRUE	igd
Hypertensio	Hypertensio	Hypertensio	TRUE	igd
Hypertensio	Hypertensio	Hypertensio	TRUE	igd
Hypertensio	Hypertensio	Hypertensio	TRUE	igd
Hypertensio	Hypertensio	Hypertensio	TRUE	igd
Hypertensio	Hypertensio	Hypertensio	TRUE	igd
Hypertensio	Hypertensio	Hypertensio	TRUE	igd
Hypertensio	Hypertensio	Hypertensio	TRUE	igd
Hypertensio	Hypertensio	Hypertensio	TRUE	igd
Hypertensio	Hypertensio	Hypertensio	TRUE	igd
Hypertensio	Hypertensio	Hypertensio	TRUE	igd
Hypertensio	Hypertensio	Hypertensio	TRUE	igd

target_a2.ou	proxy_a1.ou	proxy_a2.ou	pos.exposur	chr.exposur	samplesize.e	pval.exposu	se.exposure	id.exposure
			80940592	4	57284	5.3E-07	0.0219476	ebi-a-GCST0
			30449594	2	57284	5.4E-09	0.0138525	ebi-a-GCST0
			83734935	14	57284	9.7E-06	0.0145092	ebi-a-GCST0
			111833788	12	57284	2.3E-07	0.0163716	ebi-a-GCST0
			186711910	1	57284	1.7E-07	0.0171034	ebi-a-GCST0
			24678572	12	57284	5.4E-06	0.0182235	ebi-a-GCST0
			198898427	1	57284	9.9E-06	0.0160287	ebi-a-GCST0
			10249906	9	57284	9.9E-06	0.0141079	ebi-a-GCST0
			36815090	21	57284	2.2E-06	0.0161983	ebi-a-GCST0
			21979096	22	57284	1.4E-06	0.0112137	ebi-a-GCST0
			114150717	1	57284	1.4E-06	0.0188986	ebi-a-GCST0
			27782743	4	57284	4.7E-07	0.0852317	ebi-a-GCST0
			118696022	11	57284	3.6E-15	0.0182235	ebi-a-GCST0
			34710338	9	57284	2.1E-13	0.0179037	ebi-a-GCST0
			62460013	2	57284	5.5E-06	0.0229344	ebi-a-GCST0
			27228184	6	57284	9.3E-08	0.0356101	ebi-a-GCST0
			191943742	2	57284	6.7E-19	0.017595	ebi-a-GCST0
			26120001	4	57284	8.8E-17	0.0179037	ebi-a-GCST0
			154425456	1	57284	3.5E-09	0.0163716	ebi-a-GCST0
			117264336	1	57284	6.2E-07	0.0183877	ebi-a-GCST0
			61092858	2	57284	5.7E-13	0.0134864	ebi-a-GCST0
G	A	G	36528957	11	57284	1.8E-06	0.0234618	ebi-a-GCST0
			35376082	14	57284	0.0000015	0.0139791	ebi-a-GCST0
			192752605	3	57284	4.5E-06	0.0161983	ebi-a-GCST0
			10727357	4	57284	5E-09	0.0171034	ebi-a-GCST0
			32586831	6	57284	2.4E-37	0.0195006	ebi-a-GCST0
			189225809	3	57284	7.1E-06	0.0278362	ebi-a-GCST0
			129542100	8	57284	1.4E-06	0.023301	ebi-a-GCST0
			167540842	6	57284	6.1E-30	0.0117293	ebi-a-GCST0
			58108052	12	57284	1.9E-07	0.0141079	ebi-a-GCST0
			28078571	3	57284	5.5E-06	0.0142392	ebi-a-GCST0
			65598241	2	57284	1.2E-14	0.0138525	ebi-a-GCST0
G	C	T	2523811	1	57284	3.3E-14	0.0169144	ebi-a-GCST0
			43855067	21	57284	3.7E-12	0.0139791	ebi-a-GCST0
			123640500	9	57284	1E-09	0.0142392	ebi-a-GCST0
			111502338	10	57284	9.6E-06	0.0161983	ebi-a-GCST0
			173349725	1	57284	3.1E-10	0.0172967	ebi-a-GCST0
			159489791	6	57284	3.3E-10	0.0182235	ebi-a-GCST0
			67787715	1	57284	6.1E-07	0.0163716	ebi-a-GCST0
			44233921	6	57284	7.6E-19	0.0275853	ebi-a-GCST0
			17415655	1	57284	3.6E-09	0.0165486	ebi-a-GCST0
			17672730	1	57284	2.2E-18	0.0171034	ebi-a-GCST0
			157674997	1	57284	2.1E-08	0.0139791	ebi-a-GCST0
			11262477	19	57284	0.000001	0.0190753	ebi-a-GCST0
			171677350	3	57284	5.3E-07	0.0143729	ebi-a-GCST0
			114377568	1	57284	1E-149	0.0201233	ebi-a-GCST0

			28612886	13	57284	1.1E-06	0.0169144	ebi-a-GCST0
			64052447	11	57284	8.1E-06	0.0107415	ebi-a-GCST0
			102608924	5	57284	1.9E-09	0.0109726	ebi-a-GCST0
			50097819	10	57284	8.8E-08	0.0139791	ebi-a-GCST0
			11341880	8	57284	4.8E-12	0.0114657	ebi-a-GCST0
			38278579	1	57284	3.6E-12	0.0134864	ebi-a-GCST0
G	T	C	32658801	6	57284	1E-200	0.0161983	ebi-a-GCST0
			204738919	2	57284	1.7E-22	0.0172967	ebi-a-GCST0
			197800965	1	57284	4.5E-06	0.0190753	ebi-a-GCST0
			128580042	7	57284	1.1E-14	0.0172967	ebi-a-GCST0
			68749927	14	57284	7.1E-10	0.0165486	ebi-a-GCST0
			27764623	3	57284	1.9E-08	0.0112137	ebi-a-GCST0
			8104722	10	57284	5.4E-08	0.0108558	ebi-a-GCST0
			67545498	18	57284	1.9E-06	0.0107415	ebi-a-GCST0
			203743798	1	57284	5.8E-06	0.072538	ebi-a-GCST0
			44749251	20	57284	8.999E-15	0.011596	ebi-a-GCST0
			92236829	7	57284	1.9E-07	0.0171034	ebi-a-GCST0
			95311422	11	57284	3E-08	0.0172967	ebi-a-GCST0
			30730679	16	57284	3E-07	0.0138525	ebi-a-GCST0
			11839326	16	57284	5.5E-07	0.0141079	ebi-a-GCST0
			111275133	11	57284	1.3E-06	0.0185549	ebi-a-GCST0
			17046866	3	57284	7.2E-10	0.0138525	ebi-a-GCST0
A	T	C	204600731	2	57284	8.4E-07	0.0171034	ebi-a-GCST0
T	C	T	38031857	17	57284	1.2E-12	0.0171034	ebi-a-GCST0
C	G	A	91005213	15	57284	3.9E-06	0.0185549	ebi-a-GCST0
			123261530	4	57284	0.0000032	0.029845	ebi-a-GCST0
			149823896	6	57284	0.000001	0.0165486	ebi-a-GCST0
			12722870	3	57284	5.3E-06	0.0143729	ebi-a-GCST0
			42997574	13	57284	5.2E-06	0.0161983	ebi-a-GCST0
			100806514	2	57284	6.7E-15	0.0139791	ebi-a-GCST0
			28174986	7	57284	2.8E-06	0.0190753	ebi-a-GCST0
			111607832	2	57284	2.8E-06	0.0192553	ebi-a-GCST0
			102463602	8	57284	6.2E-07	0.0161983	ebi-a-GCST0
			426268	6	57284	5.5E-11	0.0113383	ebi-a-GCST0
			81286298	8	57284	1.5E-07	0.0163716	ebi-a-GCST0
			6098949	10	57284	1.5E-10	0.0138525	ebi-a-GCST0
			63779871	10	57284	2.3E-20	0.0171513	ebi-a-GCST0
			5272580	17	57284	7.7E-08	0.0179037	ebi-a-GCST0
			107966040	11	57284	4.4E-07	0.0356101	ebi-a-GCST0
			128496952	11	57284	9.799E-11	0.0165486	ebi-a-GCST0
			58302935	3	57284	4.6E-08	0.0294026	ebi-a-GCST0
			24889399	3	57284	7E-06	0.0344088	ebi-a-GCST0
			14103212	6	57284	1.8E-07	0.0259482	ebi-a-GCST0
			138008508	6	57284	2.2E-07	0.0387683	ebi-a-GCST0
			44284508	6	57284	2.2E-09	0.0250971	ebi-a-GCST0
A	G	A	56394954	12	57284	4.4E-10	0.0142392	ebi-a-GCST0
			55444683	5	57284	7.3E-24	0.0181157	ebi-a-GCST0

138227364	6	57284	2.7E-26	0.0291625	ebi-a-GCST0
31415106	10	57284	1.2E-07	0.0141079	ebi-a-GCST0
46253650	3	57284	4.1E-07	0.0224302	ebi-a-GCST0
69991417	15	57284	6.5E-19	0.0130272	ebi-a-GCST0
38834033	15	57284	4.8E-16	0.0174944	ebi-a-GCST0
42193897	17	57284	5.9E-06	0.0266254	ebi-a-GCST0
12881361	18	57284	1E-15	0.0176967	ebi-a-GCST0
34775444	21	57284	2.7E-07	0.0142392	ebi-a-GCST0
29731791	6	57284	9.701E-16	0.0313107	ebi-a-GCST0
76934183	2	57284	4.6E-10	0.0234618	ebi-a-GCST0
33051683	6	57284	1.5E-85	0.0134272	ebi-a-GCST0
106667535	6	57284	2.1E-07	0.0171034	ebi-a-GCST0
6390450	10	57284	1.5E-08	0.0180622	ebi-a-GCST0
40368069	13	57284	4.6E-12	0.0167295	ebi-a-GCST0
61595564	11	57284	7.3E-07	0.0221863	ebi-a-GCST0
136402060	3	57284	9E-08	0.0672292	ebi-a-GCST0

exposure	mr_keep.ex	pval_origin.e	data_source	action	mr_keep	reliability.ex	R2	F
|| id:ebi-a-(	TRUE	reported	igd	2	TRUE	high	0.0003222	18.464294
|| id:ebi-a-(	TRUE	reported	igd	2	TRUE	high	0.0006752	38.700655
|| id:ebi-a-(	TRUE	reported	igd	2	TRUE	high	0.0003795	21.744224
|| id:ebi-a-(	TRUE	reported	igd	2	TRUE	high	0.0004526	25.938391
|| id:ebi-a-(	TRUE	reported	igd	2	TRUE	high	0.0005305	30.40478
|| id:ebi-a-(	TRUE	reported	igd	2	TRUE	high	0.0004773	27.352688
|| id:ebi-a-(	TRUE	reported	igd	2	TRUE	high	0.0002601	14.901304
|| id:ebi-a-(	TRUE	reported	igd	2	TRUE	high	0.0004013	22.998849
|| id:ebi-a-(	TRUE	reported	igd	2	TRUE	high	0.0003503	20.07098
|| id:ebi-a-(	TRUE	reported	igd	2	TRUE	high	0.0009643	55.287559
|| id:ebi-a-(	TRUE	reported	igd	2	TRUE	high	0.0004438	25.433395
|| id:ebi-a-(	TRUE	reported	igd	2	TRUE	high	0.0004457	25.544632
|| id:ebi-a-(	TRUE	reported	igd	2	TRUE	high	0.0009017	51.695112
|| id:ebi-a-(	TRUE	reported	igd	2	TRUE	high	0.000699	40.066496
|| id:ebi-a-(	TRUE	reported	igd	2	TRUE	high	0.0002951	16.909548
|| id:ebi-a-(	TRUE	reported	igd	2	TRUE	high	0.0004777	27.378145
|| id:ebi-a-(	TRUE	reported	igd	2	TRUE	high	0.0009672	55.454209
|| id:ebi-a-(	TRUE	reported	igd	2	TRUE	high	0.0010551	60.501272
|| id:ebi-a-(	TRUE	reported	igd	2	TRUE	high	0.0004526	25.938391
|| id:ebi-a-(	TRUE	reported	igd	2	TRUE	high	0.0003833	21.964424
|| id:ebi-a-(	TRUE	reported	igd	2	TRUE	high	0.0012312	70.611409
|| id:ebi-a-(	TRUE	reported	igd	2	TRUE	high	0.0004305	24.669906
|| id:ebi-a-(	TRUE	reported	igd	2	TRUE	high	0.0005288	30.308736
|| id:ebi-a-(	TRUE	reported	igd	2	TRUE	high	0.0003503	20.07098
|| id:ebi-a-(	TRUE	reported	igd	2	TRUE	high	0.0005305	30.40478
|| id:ebi-a-(	TRUE	reported	igd	2	TRUE	high	0.0031261	179.63202
|| id:ebi-a-(	TRUE	reported	igd	2	TRUE	high	0.0003364	19.276815
|| id:ebi-a-(	TRUE	reported	igd	2	TRUE	high	0.0004128	23.654721
|| id:ebi-a-(	TRUE	reported	igd	2	TRUE	high	0.0028781	165.33919
|| id:ebi-a-(	TRUE	reported	igd	2	TRUE	high	0.0004013	22.998849
|| id:ebi-a-(	TRUE	reported	igd	2	TRUE	high	0.0002922	16.745083
|| id:ebi-a-(	TRUE	reported	igd	2	TRUE	high	0.001167	66.928429
|| id:ebi-a-(	TRUE	reported	igd	2	TRUE	high	0.0008279	47.465379
|| id:ebi-a-(	TRUE	reported	igd	2	TRUE	high	0.000972	55.730707
|| id:ebi-a-(	TRUE	reported	igd	2	TRUE	high	0.000639	36.627178
|| id:ebi-a-(	TRUE	reported	igd	2	TRUE	high	0.0003503	20.07098
|| id:ebi-a-(	TRUE	reported	igd	2	TRUE	high	0.0006473	37.103775
|| id:ebi-a-(	TRUE	reported	igd	2	TRUE	high	0.0004773	27.352688
|| id:ebi-a-(	TRUE	reported	igd	2	TRUE	high	0.0004526	25.938391
|| id:ebi-a-(	TRUE	reported	igd	2	TRUE	high	0.0012238	70.189865
|| id:ebi-a-(	TRUE	reported	igd	2	TRUE	high	0.0005667	32.477624
|| id:ebi-a-(	TRUE	reported	igd	2	TRUE	high	0.0009742	55.860638
|| id:ebi-a-(	TRUE	reported	igd	2	TRUE	high	0.0005288	30.308736
|| id:ebi-a-(	TRUE	reported	igd	2	TRUE	high	0.0003562	20.409477
|| id:ebi-a-(	TRUE	reported	igd	2	TRUE	high	0.0005003	28.670643
|| id:ebi-a-(	TRUE	reported	igd	2	TRUE	high	0.0149491	869.30996

|| id:ebi-a-(	TRUE	reported	igd	2	TRUE	high	0.000424	24.300326
|| id:ebi-a-(	TRUE	reported	igd	2	TRUE	high	0.0005789	33.181118
|| id:ebi-a-(	TRUE	reported	igd	2	TRUE	high	0.001007	57.74391
|| id:ebi-a-(	TRUE	reported	igd	2	TRUE	high	0.0005288	30.308736
|| id:ebi-a-(	TRUE	reported	igd	2	TRUE	high	0.0014719	84.439073
|| id:ebi-a-(	TRUE	reported	igd	2	TRUE	high	0.0012312	70.611409
|| id:ebi-a-(	TRUE	reported	igd	2	TRUE	high	0.0130779	759.05708
|| id:ebi-a-(	TRUE	reported	igd	2	TRUE	high	0.0011304	64.822173
|| id:ebi-a-(	TRUE	reported	igd	2	TRUE	high	0.0003562	20.409477
|| id:ebi-a-(	TRUE	reported	igd	2	TRUE	high	0.0006473	37.103775
|| id:ebi-a-(	TRUE	reported	igd	2	TRUE	high	0.0005667	32.477624
|| id:ebi-a-(	TRUE	reported	igd	2	TRUE	high	0.0009643	55.287559
|| id:ebi-a-(	TRUE	reported	igd	2	TRUE	high	0.0007795	44.687267
|| id:ebi-a-(	TRUE	reported	igd	2	TRUE	high	0.0005789	33.181118
|| id:ebi-a-(	TRUE	reported	igd	2	TRUE	high	0.0003579	20.508552
|| id:ebi-a-(	TRUE	reported	igd	2	TRUE	high	0.0017599	100.98892
|| id:ebi-a-(	TRUE	reported	igd	2	TRUE	high	0.0005305	30.40478
|| id:ebi-a-(	TRUE	reported	igd	2	TRUE	high	0.0006473	37.103775
|| id:ebi-a-(	TRUE	reported	igd	2	TRUE	high	0.0006752	38.700655
|| id:ebi-a-(	TRUE	reported	igd	2	TRUE	high	0.0004013	22.998849
|| id:ebi-a-(	TRUE	reported	igd	2	TRUE	high	0.0003002	17.203191
|| id:ebi-a-(	TRUE	reported	igd	2	TRUE	high	0.0006752	38.700655
|| id:ebi-a-(	TRUE	reported	igd	2	TRUE	high	0.0005305	30.40478
|| id:ebi-a-(	TRUE	reported	igd	2	TRUE	high	0.0005305	30.40478
|| id:ebi-a-(	TRUE	reported	igd	2	TRUE	high	0.0003002	17.203191
|| id:ebi-a-(	TRUE	reported	igd	2	TRUE	high	0.0003202	18.345419
|| id:ebi-a-(	TRUE	reported	igd	2	TRUE	high	0.0005667	32.477624
|| id:ebi-a-(	TRUE	reported	igd	2	TRUE	high	0.0005003	28.670643
|| id:ebi-a-(	TRUE	reported	igd	2	TRUE	high	0.0003503	20.07098
|| id:ebi-a-(	TRUE	reported	igd	2	TRUE	high	0.000972	55.730707
|| id:ebi-a-(	TRUE	reported	igd	2	TRUE	high	0.0003562	20.409477
|| id:ebi-a-(	TRUE	reported	igd	2	TRUE	high	0.0002788	15.974441
|| id:ebi-a-(	TRUE	reported	igd	2	TRUE	high	0.0003503	20.07098
|| id:ebi-a-(	TRUE	reported	igd	2	TRUE	high	0.0012063	69.184831
|| id:ebi-a-(	TRUE	reported	igd	2	TRUE	high	0.0004526	25.938391
|| id:ebi-a-(	TRUE	reported	igd	2	TRUE	high	0.0006752	38.700655
|| id:ebi-a-(	TRUE	reported	igd	2	TRUE	high	0.0014607	83.793858
|| id:ebi-a-(	TRUE	reported	igd	2	TRUE	high	0.000699	40.066496
|| id:ebi-a-(	TRUE	reported	igd	2	TRUE	high	0.0004777	27.378145
|| id:ebi-a-(	TRUE	reported	igd	2	TRUE	high	0.0005667	32.477624
|| id:ebi-a-(	TRUE	reported	igd	2	TRUE	high	0.0005529	31.687125
|| id:ebi-a-(	TRUE	reported	igd	2	TRUE	high	0.0003353	19.21237
|| id:ebi-a-(	TRUE	reported	igd	2	TRUE	high	0.0005062	29.010057
|| id:ebi-a-(	TRUE	reported	igd	2	TRUE	high	0.0004572	26.202209
|| id:ebi-a-(	TRUE	reported	igd	2	TRUE	high	0.000838	48.039845
|| id:ebi-a-(	TRUE	reported	igd	2	TRUE	high	0.000639	36.627178
|| id:ebi-a-(	TRUE	reported	igd	2	TRUE	high	0.0018434	105.78892

|| id:ebi-a-(	TRUE	reported	igd	2	TRUE	high	0.0022102	126.88686
|| id:ebi-a-(	TRUE	reported	igd	2	TRUE	high	0.0004013	22.998849
|| id:ebi-a-(	TRUE	reported	igd	2	TRUE	high	0.000471	26.991303
|| id:ebi-a-(	TRUE	reported	igd	2	TRUE	high	0.0022608	129.79786
|| id:ebi-a-(	TRUE	reported	igd	2	TRUE	high	0.000774	44.370267
|| id:ebi-a-(	TRUE	reported	igd	2	TRUE	high	0.0003162	18.116515
|| id:ebi-a-(	TRUE	reported	igd	2	TRUE	high	0.0009101	52.177855
|| id:ebi-a-(	TRUE	reported	igd	2	TRUE	high	0.000639	36.627178
|| id:ebi-a-(	TRUE	reported	igd	2	TRUE	high	0.0009884	56.676042
|| id:ebi-a-(	TRUE	reported	igd	2	TRUE	high	0.0004305	24.669906
|| id:ebi-a-(	TRUE	reported	igd	2	TRUE	high	0.0079498	459.02813
|| id:ebi-a-(	TRUE	reported	igd	2	TRUE	high	0.0005305	30.40478
|| id:ebi-a-(	TRUE	reported	igd	2	TRUE	high	0.0005824	33.381949
|| id:ebi-a-(	TRUE	reported	igd	2	TRUE	high	0.0006919	39.662372
|| id:ebi-a-(	TRUE	reported	igd	2	TRUE	high	0.0003935	22.551469
|| id:ebi-a-(	TRUE	reported	igd	2	TRUE	high	0.0004747	27.204076

SNP	effect_allele	other_allele	effect_allele	other_allele	beta.exposu	beta.outcon	eaf.exposur	eaf.outcome
rs10737481	G	T	G	T	0.1596	-0.0095	0.5497	0.5099
rs10748781	A	C	A	C	-0.1679	-0.0224	0.5755	0.6515
rs10761659	G	A	G	A	0.1272	-0.02	0.503	0.5289
rs10807943	C	T	C	T	-0.4683	0.1702	0.9006	0.9365
rs10870116	A	G	A	G	0.13	0.0066	0.2177	0.246
rs10951829	C	A	C	A	0.1291	0.0013	0.4851	0.4497
rs11382711	G	C	G	C	0.1893	0.0526	0.16	0.1862
rs11574435	T	C	T	C	0.1668	0.111	0.1193	0.132
rs11582525	G	C	G	C	-0.15	0.0468	0.1233	0.177
rs11584885	A	G	A	G	-0.1207	0.0737	0.2843	0.3071
rs11658993	T	C	T	C	0.1247	-0.0951	0.4771	0.5287
rs11735124	T	C	T	C	0.3589	0.8086	0.0159	0.0242
rs12132349	A	T	A	T	-0.1607	-0.1795	0.2734	0.2121
rs12132511	T	A	T	A	0.2568	0.3766	0.0169	0.05222
rs12536069	C	T	C	T	0.3504	0.476	0.0328	0.06841
rs13299529	A	C	A	C	0.5286	0.1647	0.0298	0.0114
rs13300483	T	C	T	C	0.145	0.1341	0.2545	0.3276
rs13800302	G	A	G	A	-0.3159	-0.2578	0.0288	0.03236
rs1391371	T	A	T	A	-0.2423	-0.0212	0.1779	0.1933
rs156109	T	C	T	C	0.1426	0.1296	0.6889	0.6262
rs1569498	T	C	T	C	0.1225	0.0097	0.2346	0.3008
rs16939895	A	G	A	G	0.1535	0.0827	0.1402	0.1769
rs18131645	C	G	C	G	0.7709	0.1028	0.0179	0.04749
rs18268870	G	A	G	A	0.5786	-0.6762	0.0109	0.01006
rs2149560	T	G	T	G	0.1327	-0.0032	0.5239	0.5531
rs2213903	T	C	T	C	0.1257	0.1708	0.661	0.6321
rs2297433	C	T	C	T	0.1563	0.263	0.7913	0.8064
rs2427516	C	G	C	G	-0.1442	0.0296	0.1531	0.1947
rs2721085	A	T	A	T	0.1145	0.1808	0.6541	0.6256
rs2836878	A	G	A	G	-0.1459	-0.1084	0.2575	0.2475
rs28514425	C	A	C	A	0.1396	-0.092	0.2724	0.1918
rs2964085	C	T	C	T	0.1251	0.0502	0.2724	0.2976
rs3024493	A	C	A	C	0.2065	-0.1366	0.166	0.1556
rs3197999	A	G	A	G	0.1791	-0.1663	0.3131	0.3928
rs34841888	C	G	C	G	-0.143	0.0756	0.8181	0.7416
rs35636125	G	A	G	A	-0.112	-0.1543	0.4771	0.4717
rs3823376	T	C	T	C	-0.1356	-0.2387	0.4672	0.4564
rs4676410	A	G	A	G	0.1677	-0.0958	0.1988	0.2717
rs4730274	C	T	C	T	-0.1116	-0.0187	0.4881	0.5083
rs4808776	A	G	A	G	-0.1164	0.1453	0.3797	0.3673
rs4921487	G	A	G	A	0.1286	-0.1417	0.6938	0.7498
rs54728771	A	T	A	T	0.3817	0.2109	0.0169	0.02117
rs56021266	T	C	T	C	0.1733	0.144	0.169	0.1419
rs6466310	A	C	A	C	0.1464	-0.0085	0.2217	0.1861
rs6798853	A	G	A	G	-0.1152	-0.0805	0.4115	0.4174
rs6865331	G	A	G	A	0.1959	-0.0574	0.1362	0.1921

rs6908236	C	A	C	A	-0.1152	-0.1531	0.4702	0.4466
rs7108648	C	T	C	T	-0.1175	0.1545	0.4026	0.3685
rs7214761	G	A	G	A	-0.1363	-0.0526	0.7664	0.7861
rs72735938	A	G	A	G	-0.1418	-0.1902	0.3032	0.1888
rs73067789	T	C	T	C	0.3218	-0.2775	0.0358	0.03828
rs74641213	T	C	T	C	0.282	-0.2428	0.0288	0.03989
rs77590396	A	C	A	C	0.188	0.1533	0.0815	0.1093
rs7936070	T	G	T	G	0.1572	0.031	0.4682	0.4158
rs9617090	T	C	T	C	-0.1207	-0.215	0.4115	0.3738
rs9988642	C	T	C	T	-0.3647	-0.5294	0.0716	0.04617

remove	palindromic	ambiguous	id.outcome	chr	pos	se.outcome	samplesize.(	pval.outcom
FALSE	FALSE	FALSE	finn-b-I9_HY	1	20171514	0.1266		0.9405
FALSE	FALSE	FALSE	finn-b-I9_HY	10	101283330	0.1336		0.8668
FALSE	FALSE	FALSE	finn-b-I9_HY	10	64445564	0.1265		0.8741
FALSE	FALSE	FALSE	finn-b-I9_HY	7	5340664	0.2647		0.5203
FALSE	FALSE	FALSE	finn-b-I9_HY	9	139287674	0.1471		0.964
FALSE	FALSE	FALSE	finn-b-I9_HY	7	5203268	0.1283		0.9919
FALSE	TRUE	FALSE	finn-b-I9_HY	7	5843808	0.1635		0.747799
FALSE	FALSE	FALSE	finn-b-I9_HY	3	46447972	0.1863		0.551099
FALSE	TRUE	FALSE	finn-b-I9_HY	1	151793571	0.1651		0.7769
FALSE	FALSE	FALSE	finn-b-I9_HY	1	1099437	0.1389		0.595799
FALSE	FALSE	FALSE	finn-b-I9_HY	17	37940808	0.1268		0.4532
FALSE	FALSE	FALSE	finn-b-I9_HY	15	82367823	0.4094		0.0482503
FALSE	TRUE	FALSE	finn-b-I9_HY	1	200875242	0.1555		0.2485
FALSE	TRUE	FALSE	finn-b-I9_HY	1	120530100	0.2834		0.1838
FALSE	FALSE	FALSE	finn-b-I9_HY	7	4909470	0.2506		0.0574897
FALSE	FALSE	FALSE	finn-b-I9_HY	9	94876863	0.6227		0.7914
FALSE	FALSE	FALSE	finn-b-I9_HY	9	117643362	0.1343		0.318
FALSE	FALSE	FALSE	finn-b-I9_HY	10	118389289	0.3615		0.4758
FALSE	TRUE	FALSE	finn-b-I9_HY	6	32603798	0.1627		0.8961
FALSE	FALSE	FALSE	finn-b-I9_HY	5	131626611	0.1313		0.3237
FALSE	FALSE	FALSE	finn-b-I9_HY	22	39714704	0.138		0.9437
FALSE	FALSE	FALSE	finn-b-I9_HY	18	12821903	0.1662		0.6186
FALSE	TRUE	FALSE	finn-b-I9_HY	7	5473610	0.3034		0.7347
FALSE	FALSE	FALSE	finn-b-I9_HY	1	73142243	0.5997		0.2595
FALSE	FALSE	FALSE	finn-b-I9_HY	9	5082106	0.1274		0.9797
FALSE	FALSE	FALSE	finn-b-I9_HY	8	17291890	0.1319		0.1955
FALSE	FALSE	FALSE	finn-b-I9_HY	20	62293460	0.1601		0.1005
FALSE	TRUE	FALSE	finn-b-I9_HY	20	61757326	0.161		0.8541
FALSE	TRUE	FALSE	finn-b-I9_HY	18	47483602	0.132		0.1708
FALSE	FALSE	FALSE	finn-b-I9_HY	21	40465534	0.1475		0.462701
FALSE	FALSE	FALSE	finn-b-I9_HY	4	67647605	0.163		0.5723
FALSE	FALSE	FALSE	finn-b-I9_HY	5	173742104	0.1403		0.7205
FALSE	FALSE	FALSE	finn-b-I9_HY	1	206943968	0.1739		0.4322
FALSE	FALSE	FALSE	finn-b-I9_HY	3	49721532	0.1301		0.2011
FALSE	TRUE	FALSE	finn-b-I9_HY	1	1293764	0.1446		0.600999
FALSE	FALSE	FALSE	finn-b-I9_HY	7	22819391	0.1273		0.2252
FALSE	FALSE	FALSE	finn-b-I9_HY	6	29944184	0.1303		0.0668498
FALSE	FALSE	FALSE	finn-b-I9_HY	2	241563739	0.1431		0.5031
FALSE	FALSE	FALSE	finn-b-I9_HY	7	107479719	0.1269		0.8826
FALSE	FALSE	FALSE	finn-b-I9_HY	19	18380072	0.1322		0.2717
FALSE	FALSE	FALSE	finn-b-I9_HY	5	158796325	0.1487		0.3405
FALSE	TRUE	FALSE	finn-b-I9_HY	2	161296466	0.4499		0.6392
FALSE	FALSE	FALSE	finn-b-I9_HY	22	41756571	0.1812		0.4269
FALSE	FALSE	FALSE	finn-b-I9_HY	7	78233434	0.1711		0.9603
FALSE	FALSE	FALSE	finn-b-I9_HY	3	174838151	0.1289		0.5322
FALSE	FALSE	FALSE	finn-b-I9_HY	5	40326099	0.1604		0.7202

FALSE	FALSE	FALSE	finn-b-I9_HY	6	33628922	0.128	0.2315
FALSE	FALSE	FALSE	finn-b-I9_HY	11	44619277	0.1323	0.2428
FALSE	FALSE	FALSE	finn-b-I9_HY	17	46347422	0.1554	0.735099
FALSE	FALSE	FALSE	finn-b-I9_HY	4	149609218	0.1636	0.245
FALSE	FALSE	FALSE	finn-b-I9_HY	20	5216316	0.3638	0.4457
FALSE	FALSE	FALSE	finn-b-I9_HY	18	59129353	0.3197	0.4476
FALSE	FALSE	FALSE	finn-b-I9_HY	15	96120424	0.2025	0.4491
FALSE	FALSE	FALSE	finn-b-I9_HY	11	76293527	0.128	0.8088
FALSE	FALSE	FALSE	finn-b-I9_HY	22	50439194	0.1316	0.1024
FALSE	FALSE	FALSE	finn-b-I9_HY	1	67726104	0.2985	0.07615

outcome	originalnam	outcome.de	mr_keep.ou	data_source	pos.exposur	samplesize.e	chr.exposur	pval.exposu
Hypertensio	Hypertensio	Hypertensio	TRUE	igd	20171514	214053	1	5.897E-11
Hypertensio	Hypertensio	Hypertensio	TRUE	igd	101283330	214053	10	6.741E-11
Hypertensio	Hypertensio	Hypertensio	TRUE	igd	64445564	214053	10	1.856E-07
Hypertensio	Hypertensio	Hypertensio	TRUE	igd	5340664	214053	7	6.827E-19
Hypertensio	Hypertensio	Hypertensio	TRUE	igd	139287674	214053	9	4.207E-06
Hypertensio	Hypertensio	Hypertensio	TRUE	igd	5203268	214053	7	1.588E-07
Hypertensio	Hypertensio	Hypertensio	TRUE	igd	5843808	214053	7	2.257E-09
Hypertensio	Hypertensio	Hypertensio	TRUE	igd	46447972	214053	3	3.431E-06
Hypertensio	Hypertensio	Hypertensio	TRUE	igd	151793571	214053	1	3.116E-06
Hypertensio	Hypertensio	Hypertensio	TRUE	igd	1099437	214053	1	6.019E-06
Hypertensio	Hypertensio	Hypertensio	TRUE	igd	37940808	214053	17	3.353E-07
Hypertensio	Hypertensio	Hypertensio	TRUE	igd	82367823	214053	15	6.271E-06
Hypertensio	Hypertensio	Hypertensio	TRUE	igd	200875242	214053	1	9.083E-08
Hypertensio	Hypertensio	Hypertensio	TRUE	igd	120530100	214053	1	3.452E-06
Hypertensio	Hypertensio	Hypertensio	TRUE	igd	4909470	214053	7	1.358E-12
Hypertensio	Hypertensio	Hypertensio	TRUE	igd	94876863	214053	9	8.64E-06
Hypertensio	Hypertensio	Hypertensio	TRUE	igd	117643362	214053	9	2.55E-08
Hypertensio	Hypertensio	Hypertensio	TRUE	igd	118389289	214053	10	8.383E-06
Hypertensio	Hypertensio	Hypertensio	TRUE	igd	32603798	214053	6	2.104E-14
Hypertensio	Hypertensio	Hypertensio	TRUE	igd	131626611	214053	5	1.778E-08
Hypertensio	Hypertensio	Hypertensio	TRUE	igd	39714704	214053	22	4.181E-06
Hypertensio	Hypertensio	Hypertensio	TRUE	igd	12821903	214053	18	1.595E-06
Hypertensio	Hypertensio	Hypertensio	TRUE	igd	5473610	214053	7	2.739E-34
Hypertensio	Hypertensio	Hypertensio	TRUE	igd	73142243	214053	1	5.158E-06
Hypertensio	Hypertensio	Hypertensio	TRUE	igd	5082106	214053	9	5.971E-08
Hypertensio	Hypertensio	Hypertensio	TRUE	igd	17291890	214053	8	7.647E-07
Hypertensio	Hypertensio	Hypertensio	TRUE	igd	62293460	214053	20	4.302E-07
Hypertensio	Hypertensio	Hypertensio	TRUE	igd	61757326	214053	20	3.884E-06
Hypertensio	Hypertensio	Hypertensio	TRUE	igd	47483602	214053	18	6.26E-06
Hypertensio	Hypertensio	Hypertensio	TRUE	igd	40465534	214053	21	2.853E-07
Hypertensio	Hypertensio	Hypertensio	TRUE	igd	67647605	214053	4	7.682E-06
Hypertensio	Hypertensio	Hypertensio	TRUE	igd	173742104	214053	5	3.325E-06
Hypertensio	Hypertensio	Hypertensio	TRUE	igd	206943968	214053	1	1.033E-09
Hypertensio	Hypertensio	Hypertensio	TRUE	igd	49721532	214053	3	8.845E-13
Hypertensio	Hypertensio	Hypertensio	TRUE	igd	1293764	214053	1	3.016E-07
Hypertensio	Hypertensio	Hypertensio	TRUE	igd	22819391	214053	7	4.748E-06
Hypertensio	Hypertensio	Hypertensio	TRUE	igd	29944184	214053	6	5.247E-08
Hypertensio	Hypertensio	Hypertensio	TRUE	igd	241563739	214053	2	1.226E-09
Hypertensio	Hypertensio	Hypertensio	TRUE	igd	107479719	214053	7	4.855E-06
Hypertensio	Hypertensio	Hypertensio	TRUE	igd	18380072	214053	19	4.553E-06
Hypertensio	Hypertensio	Hypertensio	TRUE	igd	158796325	214053	5	5.561E-06
Hypertensio	Hypertensio	Hypertensio	TRUE	igd	161296466	214053	2	8.933E-06
Hypertensio	Hypertensio	Hypertensio	TRUE	igd	41756571	214053	22	7.396E-07
Hypertensio	Hypertensio	Hypertensio	TRUE	igd	78233434	214053	7	8.24E-06
Hypertensio	Hypertensio	Hypertensio	TRUE	igd	174838151	214053	3	3.244E-06
Hypertensio	Hypertensio	Hypertensio	TRUE	igd	40326099	214053	5	2.376E-10

Hypertensio	Hypertensio	Hypertensio	TRUE	igd	33628922	214053	6	2.955E-06
Hypertensio	Hypertensio	Hypertensio	TRUE	igd	44619277	214053	11	3.628E-06
Hypertensio	Hypertensio	Hypertensio	TRUE	igd	46347422	214053	17	4.447E-06
Hypertensio	Hypertensio	Hypertensio	TRUE	igd	149609218	214053	4	6.978E-06
Hypertensio	Hypertensio	Hypertensio	TRUE	igd	5216316	214053	20	5.311E-06
Hypertensio	Hypertensio	Hypertensio	TRUE	igd	59129353	214053	18	5.974E-06
Hypertensio	Hypertensio	Hypertensio	TRUE	igd	96120424	214053	15	1.616E-06
Hypertensio	Hypertensio	Hypertensio	TRUE	igd	76293527	214053	11	1.933E-10
Hypertensio	Hypertensio	Hypertensio	TRUE	igd	50439194	214053	22	1.766E-06
Hypertensio	Hypertensio	Hypertensio	TRUE	igd	67726104	214053	1	1.75E-09

se.exposure	id.exposure	exposure	mr_keep.ex	pval_origin.	data_source	action	mr_keep	reliability.ex
0.0244	finn-b-K11_I	|| id:finn-b-	TRUE	reported	igd	2	TRUE	high
0.0257	finn-b-K11_I	|| id:finn-b-	TRUE	reported	igd	2	TRUE	high
0.0244	finn-b-K11_I	|| id:finn-b-	TRUE	reported	igd	2	TRUE	high
0.0527	finn-b-K11_I	|| id:finn-b-	TRUE	reported	igd	2	TRUE	high
0.0283	finn-b-K11_I	|| id:finn-b-	TRUE	reported	igd	2	TRUE	high
0.0246	finn-b-K11_I	|| id:finn-b-	TRUE	reported	igd	2	TRUE	high
0.0317	finn-b-K11_I	|| id:finn-b-	TRUE	reported	igd	2	TRUE	high
0.0359	finn-b-K11_I	|| id:finn-b-	TRUE	reported	igd	2	TRUE	high
0.0322	finn-b-K11_I	|| id:finn-b-	TRUE	reported	igd	2	TRUE	high
0.0267	finn-b-K11_I	|| id:finn-b-	TRUE	reported	igd	2	TRUE	high
0.0244	finn-b-K11_I	|| id:finn-b-	TRUE	reported	igd	2	TRUE	high
0.0795	finn-b-K11_I	|| id:finn-b-	TRUE	reported	igd	2	TRUE	high
0.0301	finn-b-K11_I	|| id:finn-b-	TRUE	reported	igd	2	TRUE	high
0.0553	finn-b-K11_I	|| id:finn-b-	TRUE	reported	igd	2	TRUE	high
0.0494	finn-b-K11_I	|| id:finn-b-	TRUE	reported	igd	2	TRUE	high
0.1188	finn-b-K11_I	|| id:finn-b-	TRUE	reported	igd	2	TRUE	high
0.026	finn-b-K11_I	|| id:finn-b-	TRUE	reported	igd	2	TRUE	high
0.0709	finn-b-K11_I	|| id:finn-b-	TRUE	reported	igd	2	TRUE	high
0.0317	finn-b-K11_I	|| id:finn-b-	TRUE	reported	igd	2	TRUE	high
0.0253	finn-b-K11_I	|| id:finn-b-	TRUE	reported	igd	2	TRUE	high
0.0266	finn-b-K11_I	|| id:finn-b-	TRUE	reported	igd	2	TRUE	high
0.032	finn-b-K11_I	|| id:finn-b-	TRUE	reported	igd	2	TRUE	high
0.0631	finn-b-K11_I	|| id:finn-b-	TRUE	reported	igd	2	TRUE	high
0.1269	finn-b-K11_I	|| id:finn-b-	TRUE	reported	igd	2	TRUE	high
0.0245	finn-b-K11_I	|| id:finn-b-	TRUE	reported	igd	2	TRUE	high
0.0254	finn-b-K11_I	|| id:finn-b-	TRUE	reported	igd	2	TRUE	high
0.0309	finn-b-K11_I	|| id:finn-b-	TRUE	reported	igd	2	TRUE	high
0.0312	finn-b-K11_I	|| id:finn-b-	TRUE	reported	igd	2	TRUE	high
0.0253	finn-b-K11_I	|| id:finn-b-	TRUE	reported	igd	2	TRUE	high
0.0284	finn-b-K11_I	|| id:finn-b-	TRUE	reported	igd	2	TRUE	high
0.0312	finn-b-K11_I	|| id:finn-b-	TRUE	reported	igd	2	TRUE	high
0.0269	finn-b-K11_I	|| id:finn-b-	TRUE	reported	igd	2	TRUE	high
0.0338	finn-b-K11_I	|| id:finn-b-	TRUE	reported	igd	2	TRUE	high
0.0251	finn-b-K11_I	|| id:finn-b-	TRUE	reported	igd	2	TRUE	high
0.0279	finn-b-K11_I	|| id:finn-b-	TRUE	reported	igd	2	TRUE	high
0.0245	finn-b-K11_I	|| id:finn-b-	TRUE	reported	igd	2	TRUE	high
0.0249	finn-b-K11_I	|| id:finn-b-	TRUE	reported	igd	2	TRUE	high
0.0276	finn-b-K11_I	|| id:finn-b-	TRUE	reported	igd	2	TRUE	high
0.0244	finn-b-K11_I	|| id:finn-b-	TRUE	reported	igd	2	TRUE	high
0.0254	finn-b-K11_I	|| id:finn-b-	TRUE	reported	igd	2	TRUE	high
0.0283	finn-b-K11_I	|| id:finn-b-	TRUE	reported	igd	2	TRUE	high
0.0859	finn-b-K11_I	|| id:finn-b-	TRUE	reported	igd	2	TRUE	high
0.035	finn-b-K11_I	|| id:finn-b-	TRUE	reported	igd	2	TRUE	high
0.0328	finn-b-K11_I	|| id:finn-b-	TRUE	reported	igd	2	TRUE	high
0.0248	finn-b-K11_I	|| id:finn-b-	TRUE	reported	igd	2	TRUE	high
0.0309	finn-b-K11_I	|| id:finn-b-	TRUE	reported	igd	2	TRUE	high

0.0246	finn-b-K11_I	|| id:finn-b-	TRUE	reported	igd	2	TRUE	high
0.0254	finn-b-K11_I	|| id:finn-b-	TRUE	reported	igd	2	TRUE	high
0.0297	finn-b-K11_I	|| id:finn-b-	TRUE	reported	igd	2	TRUE	high
0.0316	finn-b-K11_I	|| id:finn-b-	TRUE	reported	igd	2	TRUE	high
0.0707	finn-b-K11_I	|| id:finn-b-	TRUE	reported	igd	2	TRUE	high
0.0623	finn-b-K11_I	|| id:finn-b-	TRUE	reported	igd	2	TRUE	high
0.0392	finn-b-K11_I	|| id:finn-b-	TRUE	reported	igd	2	TRUE	high
0.0247	finn-b-K11_I	|| id:finn-b-	TRUE	reported	igd	2	TRUE	high
0.0253	finn-b-K11_I	|| id:finn-b-	TRUE	reported	igd	2	TRUE	high
0.0606	finn-b-K11_I	|| id:finn-b-	TRUE	reported	igd	2	TRUE	high

R2	F
0.0001998	42.784067
0.0001994	42.680656
0.0001269	27.176312
0.0003688	78.962889
9.857E-05	21.101327
0.0001286	27.540905
0.0001666	35.659779
0.0001008	21.58734
0.0001014	21.700349
9.546E-05	20.435627
0.000122	26.118558
9.52E-05	20.380208
0.0001331	28.503271
0.0001007	21.564318
0.000235	50.311844
9.248E-05	19.79782
0.0001453	31.10178
9.274E-05	19.851929
0.0002729	58.423053
0.0001484	31.768298
9.907E-05	21.208251
0.0001075	23.009795
0.0006968	149.25652
9.711E-05	20.788806
0.000137	29.336319
0.0001144	24.49058
0.0001195	25.585679
9.978E-05	21.360788
9.568E-05	20.481694
0.0001233	26.391851
9.352E-05	20.019701
0.000101	21.627484
0.0001743	37.325244
0.0002378	50.914288
0.0001227	26.269972
9.762E-05	20.897764
0.0001385	29.656277
0.0001724	36.918488
9.772E-05	20.919181
9.81E-05	21.000734
9.646E-05	20.649285
9.224E-05	19.744854
0.0001145	24.516416
9.306E-05	19.921884
0.0001008	21.577322
0.0001877	40.192762

0.0001024	21.929599
9.996E-05	21.39953
9.838E-05	21.060795
9.406E-05	20.136048
9.678E-05	20.717111
9.571E-05	20.488873
0.0001074	23.000618
0.0001892	40.504858
0.0001063	22.759852
0.0001692	36.217813

SNP	effect_allele	other_allele	effect_allele	other_allele	beta.exposu	beta.outcon	eaf.exposur	eaf.outcome
rs10063294	A	G	A	G	-0.099047	-0.027	0.5577	0.6248
rs1014486	C	T	C	T	0.10508	0.04	0.4592	0.4149
rs1026916	G	A	G	A	-0.129653	-0.226	0.6342	0.6164
rs10495961	T	C	T	C	0.0967637	0.0113	0.1799	0.1183
rs1077667	T	C	T	C	-0.151862	-0.2109	0.2286	0.3134
rs10801908	T	C	T	C	-0.21495	-0.2631	0.1382	0.1752
rs1087056	A	G	A	G	0.0775292	0.0773	0.4215	0.5219
rs10898270	T	G	T	G	0.0957646	-0.2924	0.6143	0.6001
rs10914539	T	G	T	G	0.133189	-0.0036	0.1759	0.1677
rs10951042	C	T	C	T	0.0785339	-0.0615	0.3708	0.4333
rs1112718	G	A	G	A	-0.10562	0.2052	0.3926	0.4061
rs11152081	G	A	G	A	0.0832376	0.068	0.666	0.6083
rs11190283	T	C	T	C	0.0830556	0.2269	0.2555	0.3274
rs11256593	T	C	T	C	0.186314	0.0219	0.5547	0.5094
rs1145160	C	A	C	A	-0.082041	-0.0012	0.4016	0.4575
rs11487278	T	C	T	C	-2.45044	-0.1776	0.0189	0.01016
rs11577513	A	G	A	G	-0.087828	0.1572	0.2316	0.2534
rs11629628	A	G	A	G	0.355675	-0.2515	0.0417	0.0728
rs11666263	G	A	G	A	-0.102827	0.0115	0.3091	0.3479
rs11711621	T	C	T	C	-0.10427	-0.0457	0.2425	0.2727
rs11749040	A	G	A	G	0.196745	-0.0595	0.1352	0.1927
rs11762408	A	C	A	C	-0.479706	-0.8284	0.0318	0.02024
rs1177228	G	A	G	A	0.107418	-0.0571	0.7555	0.7495
rs11809700	T	C	T	C	0.144448	-0.1762	0.2853	0.2412
rs11852059	C	A	C	A	0.0965109	-0.0319	0.1938	0.1861
rs1204649	C	A	C	A	-0.087957	-0.0282	0.4364	0.4029
rs12147246	G	A	G	A	-0.099378	-0.1486	0.6551	0.6258
rs12211604	A	G	A	G	-0.093103	0.0405	0.6243	0.5992
rs12365699	A	G	A	G	-0.143754	-0.3623	0.165	0.1571
rs1250551	T	G	T	G	0.115748	0.0257	0.3111	0.4155
rs12612620	A	G	A	G	0.211833	0.1562	0.2247	0.1672
rs12925972	C	T	C	T	0.0945826	-0.0967	0.5328	0.5384
rs1323657	G	A	G	A	-0.076683	-0.0923	0.5109	0.6047
rs13327021	T	C	T	C	0.115186	-0.0708	0.3797	0.3476
rs13428812	G	A	G	A	0.0820781	0.0669	0.3499	0.3076
rs134490	T	C	T	C	0.131905	-0.2527	0.8857	0.8193
rs1365120	T	C	T	C	-0.119572	-0.0963	0.9036	0.9026
rs140522	C	T	C	T	-0.110596	0.3313	0.6799	0.6856
rs1465697	T	C	T	C	0.124317	-0.2755	0.2376	0.1703
rs16822584	T	C	T	C	-0.292968	-0.232	0.0239	0.06098
rs17051321	T	C	T	C	0.0946404	0.2404	0.2207	0.1943
rs17124032	A	G	A	G	-0.216803	-0.0168	0.0815	0.06476
rs17313062	G	A	G	A	-0.085076	0.2543	0.2913	0.2601
rs1738074	C	T	C	T	0.113729	-0.1084	0.5666	0.5831
rs17724508	C	T	C	T	-0.208071	-0.0647	0.0408	0.05552
rs1793397	G	A	G	A	-0.109034	0.0052	0.8579	0.8217

rs1860545	A	G	A	G	0.116534	-0.158	0.4225	0.422
rs1887427	G	A	G	A	-0.085168	-0.1061	0.2594	0.2502
rs1992382	A	G	A	G	-0.151862	-0.3873	0.0626	0.06366
rs1997768	G	A	G	A	-0.118227	0.0016	0.1571	0.1469
rs2007403	T	C	T	C	0.0813185	-0.3397	0.6262	0.5369
rs2084007	T	C	T	C	-0.079366	-0.0069	0.508	0.5296
rs2150879	A	G	A	G	-0.103549	0.0397	0.5567	0.4974
rs2242508	G	A	G	A	-0.088831	0.0276	0.4135	0.3617
rs2248461	A	G	A	G	-0.108142	-0.1776	0.3817	0.3201
rs2269434	C	T	C	T	0.0861027	-0.0648	0.3469	0.4426
rs2289746	C	T	C	T	0.085168	-0.0521	0.66	0.6408
rs2317231	T	G	T	G	-0.100569	0.2294	0.4672	0.415
rs2546890	G	A	G	A	-0.116983	0.2076	0.5089	0.4107
rs2681424	C	T	C	T	-0.121155	-0.0659	0.506	0.4693
rs2857700	C	T	C	T	-0.767224	-0.3882	0.8946	0.8743
rs28703878	G	A	G	A	0.133646	-0.2645	0.3201	0.2634
rs290260	T	C	T	C	-0.103819	-0.0545	0.2167	0.2392
rs34299154	A	C	A	C	-0.257584	-0.3515	0.0974	0.03698
rs34550882	T	C	T	C	0.139492	-0.0275	0.1441	0.09979
rs34681760	C	T	C	T	0.0801965	-0.0429	0.6501	0.631
rs34695601	C	T	C	T	-0.109482	-0.0153	0.2495	0.1967
rs354033	A	G	A	G	-0.107957	-0.1653	0.2604	0.199
rs35486093	G	A	G	A	0.179486	0.2015	0.0865	0.06937
rs35540610	C	T	C	T	0.135143	-0.3649	0.2117	0.1712
rs35703946	A	G	A	G	-0.172524	-0.0934	0.1372	0.1671
rs3737798	G	A	G	A	-0.087186	0.0749	0.4712	0.4507
rs3783196	A	C	A	C	-0.08333	-0.0547	0.502	0.5563
rs3795131	C	T	C	T	-0.078904	0.1528	0.2982	0.2928
rs3809627	A	C	A	C	-0.096952	0.0955	0.4165	0.4832
rs415759	C	T	C	T	0.118784	-0.0846	0.1889	0.1381
rs4325907	T	C	T	C	-0.099268	-0.0438	0.6531	0.6528
rs438613	C	T	C	T	0.138021	0.0352	0.4871	0.426
rs4707386	A	G	A	G	-0.087461	0.1933	0.2684	0.2752
rs478093	G	A	G	A	0.105138	-0.0986	0.6859	0.6687
rs4796224	G	A	G	A	0.085449	-0.1138	0.4503	0.4406
rs4944014	T	C	T	C	-0.092488	0.2299	0.2237	0.2186
rs4947255	T	C	T	C	-0.552217	0.2552	0.0378	0.04917
rs55970742	T	C	T	C	-0.100373	-0.1522	0.6789	0.6394
rs56232455	A	G	A	G	0.15841	0.0576	0.4433	0.5206
rs56272720	G	A	G	A	0.478681	-0.1983	0.0467	0.0399
rs57532281	T	C	T	C	0.083599	0.203	0.4404	0.367
rs58546351	G	A	G	A	-1.89446	0.3674	0.0338	0.008804
rs59655222	C	T	C	T	-0.123191	-0.1574	0.2734	0.2126
rs6012503	G	A	G	A	0.0877389	0.0093	0.4891	0.4081
rs6032662	T	C	T	C	-0.133831	-0.112	0.7425	0.7281
rs60600003	G	T	G	T	0.133874	0.0488	0.0974	0.1022
rs62013236	T	C	T	C	-0.125222	0.1476	0.1451	0.1486

rs62195662	C	T	C	T	0.48776	-0.1786	0.0457	0.0902
rs62420820	A	G	A	G	0.137237	0.1237	0.2276	0.1894
rs62512839	G	A	G	A	0.0757566	0.0237	0.3837	0.3344
rs6496663	C	A	C	A	0.100594	0.1248	0.3141	0.2383
rs6509314	T	C	T	C	0.100117	-0.1698	0.7276	0.733
rs6564681	T	C	T	C	-0.094421	0.0953	0.6849	0.722
rs6589706	G	A	G	A	-0.084341	-0.0497	0.5467	0.5787
rs6670198	C	T	C	T	-0.145026	0.2656	0.333	0.3679
rs67111717	G	A	G	A	0.0955829	0.0894	0.3479	0.3572
rs6742	C	T	C	T	0.159309	-0.0749	0.7913	0.8041
rs6763437	A	G	A	G	-0.779141	0.2762	0.0417	0.009696
rs6837324	G	A	G	A	0.0863207	0.0475	0.3767	0.3443
rs6916491	C	T	C	T	-0.079273	0.0729	0.3718	0.4471
rs6990534	G	A	G	A	0.10714	-0.024	0.669	0.6706
rs701006	G	A	G	A	0.113864	0.2323	0.6074	0.5663
rs7190580	G	A	G	A	-0.098034	-0.2204	0.7346	0.679
rs7200146	T	G	T	G	-0.170788	-0.2904	0.6292	0.5659
rs72855540	G	A	G	A	0.18382	0.4048	0.0338	0.03198
rs72922276	A	G	A	G	-0.132168	0.0604	0.0905	0.116
rs72928038	A	G	A	G	0.160521	-0.1393	0.1799	0.1145
rs7385730	T	G	T	G	-0.123279	-0.1219	0.1938	0.1516
rs73902837	T	C	T	C	-0.250837	0.0256	0.0278	0.03818
rs743771	A	C	A	C	-0.099393	0.0159	0.4851	0.455
rs74449127	G	A	G	A	-0.196964	-0.0094	0.2654	0.3236
rs750377	T	C	T	C	0.0912385	0.0195	0.1978	0.1832
rs7592560	A	G	A	G	0.10409	0.0982	0.5666	0.5486
rs7698247	A	G	A	G	-0.089378	0.0324	0.6829	0.7212
rs7731626	A	G	A	G	-0.092944	0.0683	0.3777	0.2787
rs7830997	G	A	G	A	-0.084143	0.0847	0.6312	0.6662
rs7855251	C	T	C	T	-0.110109	-0.0403	0.2843	0.2371
rs7975763	T	C	T	C	0.121038	0.0242	0.2157	0.1738
rs8062446	T	C	T	C	0.0848006	-0.094	0.4046	0.3981
rs9277647	T	C	T	C	-0.213812	0.4628	0.1829	0.1834
rs9308424	A	G	A	G	-0.091239	-0.0178	0.3231	0.2141
rs9327104	G	A	G	A	0.310337	0.1021	0.0199	0.03255
rs9370777	G	A	G	A	-0.099158	0.0018	0.2445	0.1142
rs9557185	T	C	T	C	-0.132956	-0.1881	0.1143	0.1325
rs9591325	C	T	C	T	-0.212366	0.2021	0.0547	0.06201
rs9599826	T	C	T	C	-0.076868	0.1857	0.4404	0.5038
rs9610458	T	C	T	C	0.114221	0.1254	0.5417	0.4153
rs9844930	C	A	C	A	-0.080451	-0.1214	0.3738	0.3483
rs9878602	G	T	G	T	-0.083606	0.177	0.5	0.4914
rs9955954	G	A	G	A	-0.110038	0.274	0.2207	0.2134
rs9977672	A	G	A	G	-0.092215	-0.0698	0.2515	0.237
rs9992763	T	G	T	G	-0.090034	0.0916	0.5577	0.5013

remove	palindromic	ambiguous	id.outcome	chr	pos	se.outcome	samplesize.(	pval.outcom
FALSE	FALSE	FALSE	finn-b-I9_HY	5	35877505	0.1317		0.8376
FALSE	FALSE	FALSE	finn-b-I9_HY	3	159691112	0.129		0.7565
FALSE	FALSE	FALSE	finn-b-I9_HY	17	40529835	0.1304		0.083029
FALSE	FALSE	FALSE	finn-b-I9_HY	2	49034729	0.1972		0.9542
FALSE	FALSE	FALSE	finn-b-I9_HY	19	6668972	0.1364		0.1221
FALSE	FALSE	FALSE	finn-b-I9_HY	1	117090493	0.1659		0.1129
FALSE	FALSE	FALSE	finn-b-I9_HY	10	31395761	0.1274		0.5439
FALSE	FALSE	FALSE	finn-b-I9_HY	11	84303787	0.1307		0.0252802
FALSE	FALSE	FALSE	finn-b-I9_HY	1	32715641	0.1711		0.9831
FALSE	FALSE	FALSE	finn-b-I9_HY	7	3139417	0.1285		0.632
FALSE	FALSE	FALSE	finn-b-I9_HY	10	94479107	0.1284		0.1101
FALSE	FALSE	FALSE	finn-b-I9_HY	18	56213438	0.1299		0.6004
FALSE	FALSE	FALSE	finn-b-I9_HY	10	101547276	0.1344		0.0914008
FALSE	FALSE	FALSE	finn-b-I9_HY	10	6117322	0.1274		0.8638
FALSE	FALSE	FALSE	finn-b-I9_HY	3	9250645	0.1275		0.9924
FALSE	FALSE	FALSE	finn-b-I9_HY	6	32241452	0.6481		0.784099
FALSE	FALSE	FALSE	finn-b-I9_HY	1	179731272	0.1458		0.281
FALSE	FALSE	FALSE	finn-b-I9_HY	15	89261248	0.2452		0.3051
FALSE	FALSE	FALSE	finn-b-I9_HY	19	10590684	0.1339		0.9316
FALSE	FALSE	FALSE	finn-b-I9_HY	3	169524016	0.1432		0.749799
FALSE	FALSE	FALSE	finn-b-I9_HY	5	40396425	0.1601		0.709999
FALSE	FALSE	FALSE	finn-b-I9_HY	7	150373728	0.4562		0.0693697
FALSE	FALSE	FALSE	finn-b-I9_HY	2	61242410	0.1461		0.696
FALSE	FALSE	FALSE	finn-b-I9_HY	1	93152635	0.1481		0.2339
FALSE	FALSE	FALSE	finn-b-I9_HY	14	52306091	0.1637		0.8456
FALSE	FALSE	FALSE	finn-b-I9_HY	11	65702776	0.1294		0.8273
FALSE	FALSE	FALSE	finn-b-I9_HY	14	103265844	0.1311		0.257
FALSE	FALSE	FALSE	finn-b-I9_HY	6	7118990	0.1287		0.752801
FALSE	FALSE	FALSE	finn-b-I9_HY	11	118743286	0.1753		0.0387097
FALSE	FALSE	FALSE	finn-b-I9_HY	10	81059335	0.1293		0.8426
FALSE	FALSE	FALSE	finn-b-I9_HY	2	112488876	0.1714		0.3621
FALSE	FALSE	FALSE	finn-b-I9_HY	16	79111297	0.127		0.4465
FALSE	FALSE	FALSE	finn-b-I9_HY	10	6072427	0.1303		0.4788
FALSE	FALSE	FALSE	finn-b-I9_HY	3	27783015	0.133		0.5946
FALSE	FALSE	FALSE	finn-b-I9_HY	2	25492467	0.1375		0.626599
FALSE	FALSE	FALSE	finn-b-I9_HY	22	28730175	0.164		0.1233
FALSE	FALSE	FALSE	finn-b-I9_HY	11	36438075	0.2122		0.6498
FALSE	FALSE	FALSE	finn-b-I9_HY	22	50971266	0.137		0.0155801
FALSE	FALSE	FALSE	finn-b-I9_HY	19	49837246	0.1708		0.1068
FALSE	FALSE	FALSE	finn-b-I9_HY	6	32407537	0.2697		0.3897
FALSE	FALSE	FALSE	finn-b-I9_HY	4	122119449	0.1613		0.136
FALSE	FALSE	FALSE	finn-b-I9_HY	14	88546009	0.2573		0.948
FALSE	FALSE	FALSE	finn-b-I9_HY	1	160717741	0.1454		0.0802103
FALSE	FALSE	FALSE	finn-b-I9_HY	6	159465977	0.1283		0.398
FALSE	FALSE	FALSE	finn-b-I9_HY	16	79350204	0.2774		0.8155
FALSE	FALSE	FALSE	finn-b-I9_HY	11	75212507	0.1661		0.9751

FALSE	FALSE	FALSE	finn-b-I9_HY	12	6446777	0.1279	0.2168
FALSE	FALSE	FALSE	finn-b-I9_HY	9	4979730	0.1459	0.4673
FALSE	FALSE	FALSE	finn-b-I9_HY	3	16928092	0.2616	0.1387
FALSE	FALSE	FALSE	finn-b-I9_HY	6	26217728	0.1782	0.9928
FALSE	FALSE	FALSE	finn-b-I9_HY	4	106131210	0.1273	0.007614
FALSE	FALSE	FALSE	finn-b-I9_HY	5	133891282	0.1274	0.9571
FALSE	FALSE	FALSE	finn-b-I9_HY	17	57859210	0.127	0.754401
FALSE	FALSE	FALSE	finn-b-I9_HY	7	56151489	0.1323	0.8349
FALSE	FALSE	FALSE	finn-b-I9_HY	20	52792202	0.1356	0.1902
FALSE	FALSE	FALSE	finn-b-I9_HY	11	47360412	0.1328	0.625299
FALSE	FALSE	FALSE	finn-b-I9_HY	3	105455955	0.1318	0.692901
FALSE	FALSE	FALSE	finn-b-I9_HY	1	157686337	0.1294	0.0761904
FALSE	FALSE	FALSE	finn-b-I9_HY	5	158759900	0.1283	0.1058
FALSE	FALSE	FALSE	finn-b-I9_HY	3	121769522	0.1269	0.6034
FALSE	FALSE	FALSE	finn-b-I9_HY	6	31572481	0.1958	0.04735
FALSE	FALSE	FALSE	finn-b-I9_HY	8	79417222	0.1445	0.0672496
FALSE	FALSE	FALSE	finn-b-I9_HY	9	93554953	0.1477	0.7123
FALSE	FALSE	FALSE	finn-b-I9_HY	10	64423416	0.3482	0.3128
FALSE	FALSE	FALSE	finn-b-I9_HY	16	31274875	0.213	0.8973
FALSE	FALSE	FALSE	finn-b-I9_HY	5	6712834	0.1311	0.743599
FALSE	FALSE	FALSE	finn-b-I9_HY	14	76014298	0.1603	0.9242
FALSE	FALSE	FALSE	finn-b-I9_HY	7	149289464	0.1591	0.2987
FALSE	FALSE	FALSE	finn-b-I9_HY	1	85744577	0.2473	0.4152
FALSE	FALSE	FALSE	finn-b-I9_HY	2	231121829	0.1686	0.0304299
FALSE	FALSE	FALSE	finn-b-I9_HY	16	86021505	0.1702	0.583201
FALSE	FALSE	FALSE	finn-b-I9_HY	1	160389984	0.1278	0.557799
FALSE	FALSE	FALSE	finn-b-I9_HY	13	42039288	0.128	0.6692
FALSE	FALSE	FALSE	finn-b-I9_HY	20	39769975	0.139	0.2716
FALSE	FALSE	FALSE	finn-b-I9_HY	16	30103160	0.1269	0.4516
FALSE	FALSE	FALSE	finn-b-I9_HY	16	1066917	0.1857	0.6487
FALSE	FALSE	FALSE	finn-b-I9_HY	3	101749022	0.1324	0.740699
FALSE	FALSE	FALSE	finn-b-I9_HY	3	28072086	0.1285	0.784
FALSE	FALSE	FALSE	finn-b-I9_HY	6	88406656	0.1417	0.1725
FALSE	FALSE	FALSE	finn-b-I9_HY	1	120255126	0.1346	0.4637
FALSE	FALSE	FALSE	finn-b-I9_HY	17	34842521	0.1276	0.3726
FALSE	FALSE	FALSE	finn-b-I9_HY	11	72527180	0.1546	0.137
FALSE	FALSE	FALSE	finn-b-I9_HY	6	32207483	0.2973	0.3907
FALSE	FALSE	FALSE	finn-b-I9_HY	7	2441337	0.1322	0.2498
FALSE	FALSE	FALSE	finn-b-I9_HY	11	323649	0.1277	0.652001
FALSE	FALSE	FALSE	finn-b-I9_HY	6	135532832	0.325	0.5418
FALSE	FALSE	FALSE	finn-b-I9_HY	7	20488882	0.1322	0.1248
FALSE	FALSE	FALSE	finn-b-I9_HY	6	32496886	0.6814	0.5898
FALSE	FALSE	FALSE	finn-b-I9_HY	1	200875897	0.1552	0.3105
FALSE	FALSE	FALSE	finn-b-I9_HY	20	47251852	0.1292	0.9426
FALSE	FALSE	FALSE	finn-b-I9_HY	20	44734310	0.1424	0.4316
FALSE	FALSE	FALSE	finn-b-I9_HY	7	37382465	0.2115	0.8174
FALSE	FALSE	FALSE	finn-b-I9_HY	15	79247482	0.1776	0.406

FALSE	FALSE	FALSE	finn-b-I9_HY	2	209561980	0.2197	0.4164
FALSE	FALSE	FALSE	finn-b-I9_HY	6	137438057	0.162	0.4449
FALSE	FALSE	FALSE	finn-b-I9_HY	8	129009401	0.1342	0.8601
FALSE	FALSE	FALSE	finn-b-I9_HY	15	90889323	0.1495	0.4036
FALSE	FALSE	FALSE	finn-b-I9_HY	19	47696626	0.1435	0.2367
FALSE	FALSE	FALSE	finn-b-I9_HY	16	79652720	0.141	0.4992
FALSE	FALSE	FALSE	finn-b-I9_HY	11	118747813	0.1294	0.7007
FALSE	FALSE	FALSE	finn-b-I9_HY	1	2520527	0.1313	0.0431201
FALSE	FALSE	FALSE	finn-b-I9_HY	5	176790162	0.1323	0.499401
FALSE	FALSE	FALSE	finn-b-I9_HY	20	62374441	0.16	0.639599
FALSE	FALSE	FALSE	finn-b-I9_HY	3	119145390	0.6579	0.674599
FALSE	FALSE	FALSE	finn-b-I9_HY	4	48127262	0.1333	0.721499
FALSE	FALSE	FALSE	finn-b-I9_HY	6	119247734	0.1275	0.5675
FALSE	FALSE	FALSE	finn-b-I9_HY	8	128814091	0.1345	0.8581
FALSE	FALSE	FALSE	finn-b-I9_HY	12	58106836	0.1284	0.0703299
FALSE	FALSE	FALSE	finn-b-I9_HY	16	11403470	0.1358	0.1044
FALSE	FALSE	FALSE	finn-b-I9_HY	16	11213449	0.1285	0.02377
FALSE	FALSE	FALSE	finn-b-I9_HY	2	144990548	0.3634	0.2652
FALSE	FALSE	FALSE	finn-b-I9_HY	1	65429319	0.1994	0.7618
FALSE	FALSE	FALSE	finn-b-I9_HY	6	90976768	0.1997	0.4854
FALSE	FALSE	FALSE	finn-b-I9_HY	7	50318938	0.1778	0.493
FALSE	FALSE	FALSE	finn-b-I9_HY	21	36464229	0.3301	0.9382
FALSE	FALSE	FALSE	finn-b-I9_HY	6	32976909	0.1344	0.906
FALSE	FALSE	FALSE	finn-b-I9_HY	1	101290432	0.1361	0.9452
FALSE	FALSE	FALSE	finn-b-I9_HY	6	114860722	0.1635	0.9053
FALSE	FALSE	FALSE	finn-b-I9_HY	2	68647001	0.1271	0.4398
FALSE	FALSE	FALSE	finn-b-I9_HY	4	40192049	0.1424	0.8201
FALSE	FALSE	FALSE	finn-b-I9_HY	5	55444683	0.1414	0.629001
FALSE	FALSE	FALSE	finn-b-I9_HY	8	71442114	0.1342	0.5279
FALSE	FALSE	FALSE	finn-b-I9_HY	9	100868189	0.1497	0.7877
FALSE	FALSE	FALSE	finn-b-I9_HY	12	123604053	0.1679	0.8854
FALSE	FALSE	FALSE	finn-b-I9_HY	16	57077094	0.13	0.4696
FALSE	FALSE	FALSE	finn-b-I9_HY	6	33083750	0.1662	0.0053521
FALSE	FALSE	FALSE	finn-b-I9_HY	1	212877776	0.1561	0.9094
FALSE	FALSE	FALSE	finn-b-I9_HY	5	118883648	0.3554	0.7739
FALSE	FALSE	FALSE	finn-b-I9_HY	6	14725236	0.2087	0.9933
FALSE	FALSE	FALSE	finn-b-I9_HY	13	99927356	0.1868	0.314
FALSE	FALSE	FALSE	finn-b-I9_HY	13	50811220	0.2649	0.4454
FALSE	FALSE	FALSE	finn-b-I9_HY	13	71894040	0.1269	0.1435
FALSE	FALSE	FALSE	finn-b-I9_HY	22	22214746	0.1294	0.3326
FALSE	FALSE	FALSE	finn-b-I9_HY	3	56953174	0.1334	0.3629
FALSE	FALSE	FALSE	finn-b-I9_HY	3	71535338	0.127	0.1633
FALSE	FALSE	FALSE	finn-b-I9_HY	18	56348044	0.1543	0.0757792
FALSE	FALSE	FALSE	finn-b-I9_HY	21	40463283	0.1508	0.643501
FALSE	FALSE	FALSE	finn-b-I9_HY	4	109058718	0.1271	0.4711

outcome	originalnam	outcome.de	mr_keep.ou	data_source	proxy.outco	target_snp.c	proxy_snp.o	target_a1.ou
Hypertensio	Hypertensio	Hypertensio	TRUE	igd				
Hypertensio	Hypertensio	Hypertensio	TRUE	igd				
Hypertensio	Hypertensio	Hypertensio	TRUE	igd				
Hypertensio	Hypertensio	Hypertensio	TRUE	igd				
Hypertensio	Hypertensio	Hypertensio	TRUE	igd				
Hypertensio	Hypertensio	Hypertensio	TRUE	igd				
Hypertensio	Hypertensio	Hypertensio	TRUE	igd				
Hypertensio	Hypertensio	Hypertensio	TRUE	igd				
Hypertensio	Hypertensio	Hypertensio	TRUE	igd				
Hypertensio	Hypertensio	Hypertensio	TRUE	igd				
Hypertensio	Hypertensio	Hypertensio	TRUE	igd				
Hypertensio	Hypertensio	Hypertensio	TRUE	igd				
Hypertensio	Hypertensio	Hypertensio	TRUE	igd	TRUE	rs11190283	rs35683214	T
Hypertensio	Hypertensio	Hypertensio	TRUE	igd				
Hypertensio	Hypertensio	Hypertensio	TRUE	igd				
Hypertensio	Hypertensio	Hypertensio	TRUE	igd				
Hypertensio	Hypertensio	Hypertensio	TRUE	igd				
Hypertensio	Hypertensio	Hypertensio	TRUE	igd				
Hypertensio	Hypertensio	Hypertensio	TRUE	igd				
Hypertensio	Hypertensio	Hypertensio	TRUE	igd				
Hypertensio	Hypertensio	Hypertensio	TRUE	igd				
Hypertensio	Hypertensio	Hypertensio	TRUE	igd				
Hypertensio	Hypertensio	Hypertensio	TRUE	igd				
Hypertensio	Hypertensio	Hypertensio	TRUE	igd				
Hypertensio	Hypertensio	Hypertensio	TRUE	igd				
Hypertensio	Hypertensio	Hypertensio	TRUE	igd				
Hypertensio	Hypertensio	Hypertensio	TRUE	igd				
Hypertensio	Hypertensio	Hypertensio	TRUE	igd	TRUE	rs12211604	rs11755724	G
Hypertensio	Hypertensio	Hypertensio	TRUE	igd				
Hypertensio	Hypertensio	Hypertensio	TRUE	igd				
Hypertensio	Hypertensio	Hypertensio	TRUE	igd				
Hypertensio	Hypertensio	Hypertensio	TRUE	igd				
Hypertensio	Hypertensio	Hypertensio	TRUE	igd				
Hypertensio	Hypertensio	Hypertensio	TRUE	igd				
Hypertensio	Hypertensio	Hypertensio	TRUE	igd				
Hypertensio	Hypertensio	Hypertensio	TRUE	igd				
Hypertensio	Hypertensio	Hypertensio	TRUE	igd				
Hypertensio	Hypertensio	Hypertensio	TRUE	igd				
Hypertensio	Hypertensio	Hypertensio	TRUE	igd				
Hypertensio	Hypertensio	Hypertensio	TRUE	igd				
Hypertensio	Hypertensio	Hypertensio	TRUE	igd				
Hypertensio	Hypertensio	Hypertensio	TRUE	igd				
Hypertensio	Hypertensio	Hypertensio	TRUE	igd				
Hypertensio	Hypertensio	Hypertensio	TRUE	igd				
Hypertensio	Hypertensio	Hypertensio	TRUE	igd				
Hypertensio	Hypertensio	Hypertensio	TRUE	igd				

Hypertensio	Hypertensio	Hypertensio	TRUE	igd				
Hypertensio	Hypertensio	Hypertensio	TRUE	igd				
Hypertensio	Hypertensio	Hypertensio	TRUE	igd				
Hypertensio	Hypertensio	Hypertensio	TRUE	igd				
Hypertensio	Hypertensio	Hypertensio	TRUE	igd				
Hypertensio	Hypertensio	Hypertensio	TRUE	igd				
Hypertensio	Hypertensio	Hypertensio	TRUE	igd				
Hypertensio	Hypertensio	Hypertensio	TRUE	igd				
Hypertensio	Hypertensio	Hypertensio	TRUE	igd				
Hypertensio	Hypertensio	Hypertensio	TRUE	igd				
Hypertensio	Hypertensio	Hypertensio	TRUE	igd				
Hypertensio	Hypertensio	Hypertensio	TRUE	igd				
Hypertensio	Hypertensio	Hypertensio	TRUE	igd				
Hypertensio	Hypertensio	Hypertensio	TRUE	igd				
Hypertensio	Hypertensio	Hypertensio	TRUE	igd				
Hypertensio	Hypertensio	Hypertensio	TRUE	igd				
Hypertensio	Hypertensio	Hypertensio	TRUE	igd				
Hypertensio	Hypertensio	Hypertensio	TRUE	igd				
Hypertensio	Hypertensio	Hypertensio	TRUE	igd				
Hypertensio	Hypertensio	Hypertensio	TRUE	igd				
Hypertensio	Hypertensio	Hypertensio	TRUE	igd				
Hypertensio	Hypertensio	Hypertensio	TRUE	igd				
Hypertensio	Hypertensio	Hypertensio	TRUE	igd	TRUE	rs35486093	rs9887812	G
Hypertensio	Hypertensio	Hypertensio	TRUE	igd				
Hypertensio	Hypertensio	Hypertensio	TRUE	igd				
Hypertensio	Hypertensio	Hypertensio	TRUE	igd				
Hypertensio	Hypertensio	Hypertensio	TRUE	igd				
Hypertensio	Hypertensio	Hypertensio	TRUE	igd				
Hypertensio	Hypertensio	Hypertensio	TRUE	igd				
Hypertensio	Hypertensio	Hypertensio	TRUE	igd				
Hypertensio	Hypertensio	Hypertensio	TRUE	igd				
Hypertensio	Hypertensio	Hypertensio	TRUE	igd				
Hypertensio	Hypertensio	Hypertensio	TRUE	igd				
Hypertensio	Hypertensio	Hypertensio	TRUE	igd				
Hypertensio	Hypertensio	Hypertensio	TRUE	igd				
Hypertensio	Hypertensio	Hypertensio	TRUE	igd				
Hypertensio	Hypertensio	Hypertensio	TRUE	igd				
Hypertensio	Hypertensio	Hypertensio	TRUE	igd				
Hypertensio	Hypertensio	Hypertensio	TRUE	igd	TRUE	rs56232455	rs7944394	A
Hypertensio	Hypertensio	Hypertensio	TRUE	igd				
Hypertensio	Hypertensio	Hypertensio	TRUE	igd				
Hypertensio	Hypertensio	Hypertensio	TRUE	igd	TRUE	rs58546351	rs15096812	G
Hypertensio	Hypertensio	Hypertensio	TRUE	igd				
Hypertensio	Hypertensio	Hypertensio	TRUE	igd				
Hypertensio	Hypertensio	Hypertensio	TRUE	igd				
Hypertensio	Hypertensio	Hypertensio	TRUE	igd				
Hypertensio	Hypertensio	Hypertensio	TRUE	igd				

Hypertensio	Hypertensio	Hypertensio	TRUE	igd				
Hypertensio	Hypertensio	Hypertensio	TRUE	igd				
Hypertensio	Hypertensio	Hypertensio	TRUE	igd				
Hypertensio	Hypertensio	Hypertensio	TRUE	igd	TRUE	rs6496663	rs7162687	C
Hypertensio	Hypertensio	Hypertensio	TRUE	igd				
Hypertensio	Hypertensio	Hypertensio	TRUE	igd				
Hypertensio	Hypertensio	Hypertensio	TRUE	igd				
Hypertensio	Hypertensio	Hypertensio	TRUE	igd				
Hypertensio	Hypertensio	Hypertensio	TRUE	igd				
Hypertensio	Hypertensio	Hypertensio	TRUE	igd				
Hypertensio	Hypertensio	Hypertensio	TRUE	igd				
Hypertensio	Hypertensio	Hypertensio	TRUE	igd				
Hypertensio	Hypertensio	Hypertensio	TRUE	igd				
Hypertensio	Hypertensio	Hypertensio	TRUE	igd				
Hypertensio	Hypertensio	Hypertensio	TRUE	igd				
Hypertensio	Hypertensio	Hypertensio	TRUE	igd				
Hypertensio	Hypertensio	Hypertensio	TRUE	igd				
Hypertensio	Hypertensio	Hypertensio	TRUE	igd				
Hypertensio	Hypertensio	Hypertensio	TRUE	igd				
Hypertensio	Hypertensio	Hypertensio	TRUE	igd				
Hypertensio	Hypertensio	Hypertensio	TRUE	igd				
Hypertensio	Hypertensio	Hypertensio	TRUE	igd				
Hypertensio	Hypertensio	Hypertensio	TRUE	igd				
Hypertensio	Hypertensio	Hypertensio	TRUE	igd				
Hypertensio	Hypertensio	Hypertensio	TRUE	igd				
Hypertensio	Hypertensio	Hypertensio	TRUE	igd				
Hypertensio	Hypertensio	Hypertensio	TRUE	igd				
Hypertensio	Hypertensio	Hypertensio	TRUE	igd				
Hypertensio	Hypertensio	Hypertensio	TRUE	igd				
Hypertensio	Hypertensio	Hypertensio	TRUE	igd				
Hypertensio	Hypertensio	Hypertensio	TRUE	igd				
Hypertensio	Hypertensio	Hypertensio	TRUE	igd				
Hypertensio	Hypertensio	Hypertensio	TRUE	igd				
Hypertensio	Hypertensio	Hypertensio	TRUE	igd				
Hypertensio	Hypertensio	Hypertensio	TRUE	igd				
Hypertensio	Hypertensio	Hypertensio	TRUE	igd				
Hypertensio	Hypertensio	Hypertensio	TRUE	igd	TRUE	rs9557185	rs9517669	T
Hypertensio	Hypertensio	Hypertensio	TRUE	igd				
Hypertensio	Hypertensio	Hypertensio	TRUE	igd				
Hypertensio	Hypertensio	Hypertensio	TRUE	igd	TRUE	rs9610458	rs4821402	C
Hypertensio	Hypertensio	Hypertensio	TRUE	igd				
Hypertensio	Hypertensio	Hypertensio	TRUE	igd				
Hypertensio	Hypertensio	Hypertensio	TRUE	igd				
Hypertensio	Hypertensio	Hypertensio	TRUE	igd				
Hypertensio	Hypertensio	Hypertensio	TRUE	igd				

target_a2.o	proxy_a1.o	proxy_a2.o	chr.exposur	pos.exposur	se.exposure	pval.exposu	samplesize.e	id.exposure
			5	35877505	0.0162642	1.13E-09	115803	ieu-b-18
			3	159691112	0.016368	1.364E-10	115803	ieu-b-18
			17	40529835	0.0174305	1.02E-13	115803	ieu-b-18
			2	49034729	0.0216668	7.97E-06	115803	ieu-b-18
			19	6668972	0.021225	8.373E-13	115803	ieu-b-18
			1	117090493	0.0263631	3.537E-16	115803	ieu-b-18
			10	31395761	0.0166266	3.117E-06	115803	ieu-b-18
			11	84303787	0.0212932	6.878E-06	115803	ieu-b-18
			1	32715641	0.0265673	5.352E-07	115803	ieu-b-18
			7	3139417	0.0165201	1.996E-06	115803	ieu-b-18
			10	94479107	0.0166876	2.463E-10	115803	ieu-b-18
			18	56213438	0.0180118	3.814E-06	115803	ieu-b-18
C	A	G	10	101539887	0.0186665	8.609E-06	115803	ieu-b-18
			10	6117322	0.0173516	6.783E-27	115803	ieu-b-18
			3	9250645	0.01735	2.261E-06	115803	ieu-b-18
			6	32241452	0.255159	7.721E-22	115803	ieu-b-18
			1	179731272	0.0193603	5.72E-06	115803	ieu-b-18
			15	89261248	0.0708008	5.071E-07	115803	ieu-b-18
			19	10590684	0.0180385	1.195E-08	115803	ieu-b-18
			3	169524016	0.0192017	5.628E-08	115803	ieu-b-18
			5	40396425	0.0233464	3.54E-17	115803	ieu-b-18
			7	150373728	0.10502	4.93E-06	115803	ieu-b-18
			2	61242410	0.0186591	8.567E-09	115803	ieu-b-18
			1	93152635	0.0183508	3.505E-15	115803	ieu-b-18
			14	52306091	0.0206692	3.022E-06	115803	ieu-b-18
			11	65702776	0.0166359	1.242E-07	115803	ieu-b-18
			14	103265844	0.0169228	4.294E-09	115803	ieu-b-18
A	A	G	6	7100029	0.0178614	1.863E-07	115803	ieu-b-18
			11	118743286	0.0228493	3.146E-10	115803	ieu-b-18
			10	81059335	0.0173687	2.662E-11	115803	ieu-b-18
			2	112488876	0.036063	4.255E-09	115803	ieu-b-18
			16	79111297	0.0170812	3.073E-08	115803	ieu-b-18
			10	6072427	0.0167201	4.512E-06	115803	ieu-b-18
			3	27783015	0.0171219	1.727E-11	115803	ieu-b-18
			2	25492467	0.0176194	3.187E-06	115803	ieu-b-18
			22	28730175	0.0265396	6.691E-07	115803	ieu-b-18
			11	36438075	0.0263673	5.764E-06	115803	ieu-b-18
			22	50971266	0.017536	2.848E-10	115803	ieu-b-18
			19	49837246	0.0187656	3.479E-11	115803	ieu-b-18
			6	32407537	0.0559016	1.599E-07	115803	ieu-b-18
			4	122119449	0.0188222	4.953E-07	115803	ieu-b-18
			14	88546009	0.0316224	7.081E-12	115803	ieu-b-18
			1	160717741	0.0181119	2.637E-06	115803	ieu-b-18
			6	159465977	0.0167056	9.908E-12	115803	ieu-b-18
			16	79350204	0.0382407	5.296E-08	115803	ieu-b-18
			11	75212507	0.0245726	9.113E-06	115803	ieu-b-18

			12	6446777	0.0170311	7.788E-12	115803	ieu-b-18
			9	4979730	0.0187594	5.625E-06	115803	ieu-b-18
			3	16928092	0.0321775	2.364E-06	115803	ieu-b-18
			6	26217728	0.0240739	9.06E-07	115803	ieu-b-18
			4	106131210	0.0169262	1.553E-06	115803	ieu-b-18
			5	133891282	0.0163184	1.153E-06	115803	ieu-b-18
			17	57859210	0.0164769	3.289E-10	115803	ieu-b-18
			7	56151489	0.0173251	2.939E-07	115803	ieu-b-18
			20	52792202	0.0174165	5.326E-10	115803	ieu-b-18
			11	47360412	0.0171606	5.236E-07	115803	ieu-b-18
			3	105455955	0.0176915	1.479E-06	115803	ieu-b-18
			1	157686337	0.0167449	1.902E-09	115803	ieu-b-18
			5	158759900	0.0164181	1.039E-12	115803	ieu-b-18
			3	121769522	0.0165779	2.707E-13	115803	ieu-b-18
			6	31572481	0.0237495	1E-200	115803	ieu-b-18
			8	79417222	0.0214335	4.507E-10	115803	ieu-b-18
			9	93554953	0.0213942	1.218E-06	115803	ieu-b-18
			10	64423416	0.0546393	2.426E-06	115803	ieu-b-18
			16	31274875	0.0280354	6.506E-07	115803	ieu-b-18
			5	6712834	0.0176122	5.277E-06	115803	ieu-b-18
			14	76014298	0.0197905	3.165E-08	115803	ieu-b-18
			7	149289464	0.0189431	1.205E-08	115803	ieu-b-18
A	T	C	1	85729820	0.0280638	1.599E-10	115803	ieu-b-18
			2	231121829	0.0193517	2.879E-12	115803	ieu-b-18
			16	86021505	0.02874	1.938E-09	115803	ieu-b-18
			1	160389984	0.0165588	1.4E-07	115803	ieu-b-18
			13	42039288	0.0164904	4.344E-07	115803	ieu-b-18
			20	39769975	0.017836	9.696E-06	115803	ieu-b-18
			16	30103160	0.0175395	3.246E-08	115803	ieu-b-18
			16	1066917	0.0217558	4.765E-08	115803	ieu-b-18
			3	101749022	0.0168311	3.682E-09	115803	ieu-b-18
			3	28072086	0.0166057	9.434E-17	115803	ieu-b-18
			6	88406656	0.0189779	4.054E-06	115803	ieu-b-18
			1	120255126	0.0179039	4.296E-09	115803	ieu-b-18
			17	34842521	0.0163132	1.623E-07	115803	ieu-b-18
			11	72527180	0.0190259	1.167E-06	115803	ieu-b-18
			6	32207483	0.0555038	2.543E-23	115803	ieu-b-18
			7	2441337	0.017899	2.05E-08	115803	ieu-b-18
G	T	C	11	321235	0.0281246	1.777E-08	115803	ieu-b-18
			6	135532832	0.0913996	1.63E-07	115803	ieu-b-18
			7	20488882	0.0172896	1.33E-06	115803	ieu-b-18
A	A	G	6	32483611	0.0531758	1E-200	115803	ieu-b-18
			1	200875897	0.0186277	3.758E-11	115803	ieu-b-18
			20	47251852	0.01675	1.622E-07	115803	ieu-b-18
			20	44734310	0.0183292	2.845E-13	115803	ieu-b-18
			7	37382465	0.0264589	4.199E-07	115803	ieu-b-18
			15	79247482	0.0263811	2.068E-06	115803	ieu-b-18

			2	209561980	0.0945293	2.471E-07	115803	ieu-b-18
			6	137438057	0.0187505	2.496E-13	115803	ieu-b-18
			8	129009401	0.0170471	8.832E-06	115803	ieu-b-18
A	G	A	15	90887584	0.0181091	2.778E-08	115803	ieu-b-18
			19	47696626	0.0199765	5.394E-07	115803	ieu-b-18
			16	79652720	0.018091	1.797E-07	115803	ieu-b-18
			11	118747813	0.0168845	5.878E-07	115803	ieu-b-18
			1	2520527	0.0176423	2.029E-16	115803	ieu-b-18
			5	176790162	0.0190107	4.96E-07	115803	ieu-b-18
			20	62374441	0.0301763	1.297E-07	115803	ieu-b-18
			3	119145390	0.132145	3.722E-09	115803	ieu-b-18
			4	48127262	0.0168809	3.162E-07	115803	ieu-b-18
			6	119247734	0.0178462	8.912E-06	115803	ieu-b-18
			8	128814091	0.018154	3.597E-09	115803	ieu-b-18
			12	58106836	0.0168357	1.349E-11	115803	ieu-b-18
			16	11403470	0.0179402	4.643E-08	115803	ieu-b-18
			16	11213449	0.0169488	7.002E-24	115803	ieu-b-18
			2	144990548	0.0404016	5.369E-06	115803	ieu-b-18
			1	65429319	0.0293887	6.884E-06	115803	ieu-b-18
			6	90976768	0.0247614	9.009E-11	115803	ieu-b-18
			7	50318938	0.0243183	3.991E-07	115803	ieu-b-18
			21	36464229	0.0539438	3.32E-06	115803	ieu-b-18
			6	32976909	0.0162027	8.552E-10	115803	ieu-b-18
			1	101290432	0.0255798	1.361E-14	115803	ieu-b-18
			6	114860722	0.020299	6.966E-06	115803	ieu-b-18
			2	68647001	0.016508	2.874E-10	115803	ieu-b-18
			4	40192049	0.0189331	2.35E-06	115803	ieu-b-18
			5	55444683	0.0198535	2.848E-06	115803	ieu-b-18
			8	71442114	0.0171923	9.87E-07	115803	ieu-b-18
			9	100868189	0.0200898	4.233E-08	115803	ieu-b-18
			12	123604053	0.0209676	7.804E-09	115803	ieu-b-18
			16	57077094	0.0172163	8.41E-07	115803	ieu-b-18
			6	33083750	0.0211677	5.474E-24	115803	ieu-b-18
			1	212877776	0.0188748	1.339E-06	115803	ieu-b-18
			5	118883648	0.0614507	4.414E-07	115803	ieu-b-18
			6	14725236	0.022425	9.791E-06	115803	ieu-b-18
C	A	G	13	99895107	0.0266336	5.974E-07	115803	ieu-b-18
			13	50811220	0.0339891	4.156E-10	115803	ieu-b-18
			13	71894040	0.0162672	2.297E-06	115803	ieu-b-18
T	G	A	22	22205353	0.0165102	4.574E-12	115803	ieu-b-18
			3	56953174	0.0173903	3.724E-06	115803	ieu-b-18
			3	71535338	0.0162332	2.601E-07	115803	ieu-b-18
			18	56348044	0.0194511	1.539E-08	115803	ieu-b-18
			21	40463283	0.0191385	1.448E-06	115803	ieu-b-18
			4	109058718	0.0164612	4.514E-08	115803	ieu-b-18

exposure	mr_keep.ex	pval_origin.e	data_source	action	mr_keep	reliability.ex	R2	F
|| id:ieu-b-1	TRUE	reported	igd	2	TRUE	high	0.0003202	37.086128
|| id:ieu-b-1	TRUE	reported	igd	2	TRUE	high	0.0003558	41.21368
|| id:ieu-b-1	TRUE	reported	igd	2	TRUE	high	0.0004775	55.327105
|| id:ieu-b-1	TRUE	reported	igd	2	TRUE	high	0.0001722	19.944717
|| id:ieu-b-1	TRUE	reported	igd	2	TRUE	high	0.0004419	51.191203
|| id:ieu-b-1	TRUE	reported	igd	2	TRUE	high	0.0005737	66.477464
|| id:ieu-b-1	TRUE	reported	igd	2	TRUE	high	0.0001877	21.742837
|| id:ieu-b-1	TRUE	reported	igd	2	TRUE	high	0.0001746	20.226495
|| id:ieu-b-1	TRUE	reported	igd	2	TRUE	high	0.000217	25.132424
|| id:ieu-b-1	TRUE	reported	igd	2	TRUE	high	0.0001951	22.598597
|| id:ieu-b-1	TRUE	reported	igd	2	TRUE	high	0.0003458	40.05872
|| id:ieu-b-1	TRUE	reported	igd	2	TRUE	high	0.0001844	21.355875
|| id:ieu-b-1	TRUE	reported	igd	2	TRUE	high	0.0001709	19.797236
|| id:ieu-b-1	TRUE	reported	igd	2	TRUE	high	0.0009946	115.29339
|| id:ieu-b-1	TRUE	reported	igd	2	TRUE	high	0.000193	22.358994
|| id:ieu-b-1	TRUE	reported	igd	2	TRUE	high	0.0007958	92.227166
|| id:ieu-b-1	TRUE	reported	igd	2	TRUE	high	0.0001777	20.579336
|| id:ieu-b-1	TRUE	reported	igd	2	TRUE	high	0.0002179	25.236135
|| id:ieu-b-1	TRUE	reported	igd	2	TRUE	high	0.0002805	32.49421
|| id:ieu-b-1	TRUE	reported	igd	2	TRUE	high	0.0002546	29.487088
|| id:ieu-b-1	TRUE	reported	igd	2	TRUE	high	0.0006129	71.016631
|| id:ieu-b-1	TRUE	reported	igd	2	TRUE	high	0.0001801	20.864058
|| id:ieu-b-1	TRUE	reported	igd	2	TRUE	high	0.0002861	33.140972
|| id:ieu-b-1	TRUE	reported	igd	2	TRUE	high	0.0005348	61.959166
|| id:ieu-b-1	TRUE	reported	igd	2	TRUE	high	0.0001882	21.802078
|| id:ieu-b-1	TRUE	reported	igd	2	TRUE	high	0.0002413	27.953982
|| id:ieu-b-1	TRUE	reported	igd	2	TRUE	high	0.0002977	34.485145
|| id:ieu-b-1	TRUE	reported	igd	2	TRUE	high	0.0002346	27.169718
|| id:ieu-b-1	TRUE	reported	igd	2	TRUE	high	0.0003417	39.580983
|| id:ieu-b-1	TRUE	reported	igd	2	TRUE	high	0.0003834	44.410418
|| id:ieu-b-1	TRUE	reported	igd	2	TRUE	high	0.0002979	34.502934
|| id:ieu-b-1	TRUE	reported	igd	2	TRUE	high	0.0002647	30.66043
|| id:ieu-b-1	TRUE	reported	igd	2	TRUE	high	0.0001816	21.033678
|| id:ieu-b-1	TRUE	reported	igd	2	TRUE	high	0.0003907	45.257231
|| id:ieu-b-1	TRUE	reported	igd	2	TRUE	high	0.0001874	21.700256
|| id:ieu-b-1	TRUE	reported	igd	2	TRUE	high	0.0002133	24.701667
|| id:ieu-b-1	TRUE	reported	igd	2	TRUE	high	0.0001776	20.564595
|| id:ieu-b-1	TRUE	reported	igd	2	TRUE	high	0.0003434	39.775007
|| id:ieu-b-1	TRUE	reported	igd	2	TRUE	high	0.0003788	43.886266
|| id:ieu-b-1	TRUE	reported	igd	2	TRUE	high	0.0002371	27.465303
|| id:ieu-b-1	TRUE	reported	igd	2	TRUE	high	0.0002183	25.281618
|| id:ieu-b-1	TRUE	reported	igd	2	TRUE	high	0.0004057	47.003849
|| id:ieu-b-1	TRUE	reported	igd	2	TRUE	high	0.0001905	22.063817
|| id:ieu-b-1	TRUE	reported	igd	2	TRUE	high	0.0004001	46.345843
|| id:ieu-b-1	TRUE	reported	igd	2	TRUE	high	0.0002556	29.604923
|| id:ieu-b-1	TRUE	reported	igd	2	TRUE	high	0.00017	19.68857

|| id:ieu-b-:	TRUE	reported	igd	2	TRUE	high	0.0004041	46.817952
|| id:ieu-b-:	TRUE	reported	igd	2	TRUE	high	0.000178	20.611401
|| id:ieu-b-:	TRUE	reported	igd	2	TRUE	high	0.0001923	22.27338
|| id:ieu-b-:	TRUE	reported	igd	2	TRUE	high	0.0002082	24.117536
|| id:ieu-b-:	TRUE	reported	igd	2	TRUE	high	0.0001993	23.080876
|| id:ieu-b-:	TRUE	reported	igd	2	TRUE	high	0.0002042	23.653857
|| id:ieu-b-:	TRUE	reported	igd	2	TRUE	high	0.0003409	39.494199
|| id:ieu-b-:	TRUE	reported	igd	2	TRUE	high	0.000227	26.288878
|| id:ieu-b-:	TRUE	reported	igd	2	TRUE	high	0.0003328	38.55312
|| id:ieu-b-:	TRUE	reported	igd	2	TRUE	high	0.0002173	25.174514
|| id:ieu-b-:	TRUE	reported	igd	2	TRUE	high	0.0002001	23.174805
|| id:ieu-b-:	TRUE	reported	igd	2	TRUE	high	0.0003114	36.070789
|| id:ieu-b-:	TRUE	reported	igd	2	TRUE	high	0.0004382	50.768253
|| id:ieu-b-:	TRUE	reported	igd	2	TRUE	high	0.000461	53.40921
|| id:ieu-b-:	TRUE	reported	igd	2	TRUE	high	0.0089314	1043.5852
|| id:ieu-b-:	TRUE	reported	igd	2	TRUE	high	0.0003356	38.879282
|| id:ieu-b-:	TRUE	reported	igd	2	TRUE	high	0.0002033	23.548003
|| id:ieu-b-:	TRUE	reported	igd	2	TRUE	high	0.0001919	22.223887
|| id:ieu-b-:	TRUE	reported	igd	2	TRUE	high	0.0002137	24.755835
|| id:ieu-b-:	TRUE	reported	igd	2	TRUE	high	0.000179	20.733667
|| id:ieu-b-:	TRUE	reported	igd	2	TRUE	high	0.0002642	30.603029
|| id:ieu-b-:	TRUE	reported	igd	2	TRUE	high	0.0002804	32.478204
|| id:ieu-b-:	TRUE	reported	igd	2	TRUE	high	0.0003531	40.903522
|| id:ieu-b-:	TRUE	reported	igd	2	TRUE	high	0.000421	48.768721
|| id:ieu-b-:	TRUE	reported	igd	2	TRUE	high	0.0003111	36.034459
|| id:ieu-b-:	TRUE	reported	igd	2	TRUE	high	0.0002393	27.722492
|| id:ieu-b-:	TRUE	reported	igd	2	TRUE	high	0.0002205	25.53458
|| id:ieu-b-:	TRUE	reported	igd	2	TRUE	high	0.000169	19.570017
|| id:ieu-b-:	TRUE	reported	igd	2	TRUE	high	0.0002638	30.553935
|| id:ieu-b-:	TRUE	reported	igd	2	TRUE	high	0.0002574	29.809746
|| id:ieu-b-:	TRUE	reported	igd	2	TRUE	high	0.0003003	34.784522
|| id:ieu-b-:	TRUE	reported	igd	2	TRUE	high	0.0005962	69.082566
|| id:ieu-b-:	TRUE	reported	igd	2	TRUE	high	0.0001834	21.238706
|| id:ieu-b-:	TRUE	reported	igd	2	TRUE	high	0.0002977	34.48392
|| id:ieu-b-:	TRUE	reported	igd	2	TRUE	high	0.0002369	27.436465
|| id:ieu-b-:	TRUE	reported	igd	2	TRUE	high	0.000204	23.630497
|| id:ieu-b-:	TRUE	reported	igd	2	TRUE	high	0.000854	98.984367
|| id:ieu-b-:	TRUE	reported	igd	2	TRUE	high	0.0002715	31.446243
|| id:ieu-b-:	TRUE	reported	igd	2	TRUE	high	0.0002739	31.723784
|| id:ieu-b-:	TRUE	reported	igd	2	TRUE	high	0.0002368	27.428136
|| id:ieu-b-:	TRUE	reported	igd	2	TRUE	high	0.0002018	23.378938
|| id:ieu-b-:	TRUE	reported	igd	2	TRUE	high	0.0108415	1269.2153
|| id:ieu-b-:	TRUE	reported	igd	2	TRUE	high	0.0003775	43.735288
|| id:ieu-b-:	TRUE	reported	igd	2	TRUE	high	0.0002369	27.437671
|| id:ieu-b-:	TRUE	reported	igd	2	TRUE	high	0.0004602	53.311257
|| id:ieu-b-:	TRUE	reported	igd	2	TRUE	high	0.000221	25.600085
|| id:ieu-b-:	TRUE	reported	igd	2	TRUE	high	0.0001945	22.530352

|| id:ieu-b-:	TRUE	reported	igd	2	TRUE	high	0.0002299	26.623919
|| id:ieu-b-:	TRUE	reported	igd	2	TRUE	high	0.0004624	53.568468
|| id:ieu-b-:	TRUE	reported	igd	2	TRUE	high	0.0001705	19.748423
|| id:ieu-b-:	TRUE	reported	igd	2	TRUE	high	0.0002664	30.856234
|| id:ieu-b-:	TRUE	reported	igd	2	TRUE	high	0.0002169	25.117092
|| id:ieu-b-:	TRUE	reported	igd	2	TRUE	high	0.0002352	27.239609
|| id:ieu-b-:	TRUE	reported	igd	2	TRUE	high	0.0002154	24.951382
|| id:ieu-b-:	TRUE	reported	igd	2	TRUE	high	0.0005832	67.573098
|| id:ieu-b-:	TRUE	reported	igd	2	TRUE	high	0.0002182	25.278814
|| id:ieu-b-:	TRUE	reported	igd	2	TRUE	high	0.0002406	27.870268
|| id:ieu-b-:	TRUE	reported	igd	2	TRUE	high	0.0003001	34.763472
|| id:ieu-b-:	TRUE	reported	igd	2	TRUE	high	0.0002257	26.147563
|| id:ieu-b-:	TRUE	reported	igd	2	TRUE	high	0.0001704	19.731212
|| id:ieu-b-:	TRUE	reported	igd	2	TRUE	high	0.0003007	34.829811
|| id:ieu-b-:	TRUE	reported	igd	2	TRUE	high	0.0003948	45.740722
|| id:ieu-b-:	TRUE	reported	igd	2	TRUE	high	0.0002578	29.859926
|| id:ieu-b-:	TRUE	reported	igd	2	TRUE	high	0.0008761	101.53816
|| id:ieu-b-:	TRUE	reported	igd	2	TRUE	high	0.0001787	20.700503
|| id:ieu-b-:	TRUE	reported	igd	2	TRUE	high	0.0001746	20.224807
|| id:ieu-b-:	TRUE	reported	igd	2	TRUE	high	0.0003628	42.024816
|| id:ieu-b-:	TRUE	reported	igd	2	TRUE	high	0.0002219	25.698293
|| id:ieu-b-:	TRUE	reported	igd	2	TRUE	high	0.0001867	21.621839
|| id:ieu-b-:	TRUE	reported	igd	2	TRUE	high	0.0003248	37.629348
|| id:ieu-b-:	TRUE	reported	igd	2	TRUE	high	0.0005117	59.288699
|| id:ieu-b-:	TRUE	reported	igd	2	TRUE	high	0.0001744	20.202238
|| id:ieu-b-:	TRUE	reported	igd	2	TRUE	high	0.0003432	39.757731
|| id:ieu-b-:	TRUE	reported	igd	2	TRUE	high	0.0001924	22.284781
|| id:ieu-b-:	TRUE	reported	igd	2	TRUE	high	0.0001892	21.915847
|| id:ieu-b-:	TRUE	reported	igd	2	TRUE	high	0.0002068	23.95292
|| id:ieu-b-:	TRUE	reported	igd	2	TRUE	high	0.0002593	30.0391
|| id:ieu-b-:	TRUE	reported	igd	2	TRUE	high	0.0002877	33.322573
|| id:ieu-b-:	TRUE	reported	igd	2	TRUE	high	0.0002095	24.261112
|| id:ieu-b-:	TRUE	reported	igd	2	TRUE	high	0.0008803	102.02564
|| id:ieu-b-:	TRUE	reported	igd	2	TRUE	high	0.0002017	23.365983
|| id:ieu-b-:	TRUE	reported	igd	2	TRUE	high	0.0002202	25.503862
|| id:ieu-b-:	TRUE	reported	igd	2	TRUE	high	0.0001688	19.551479
|| id:ieu-b-:	TRUE	reported	igd	2	TRUE	high	0.0002152	24.920034
|| id:ieu-b-:	TRUE	reported	igd	2	TRUE	high	0.000337	39.037603
|| id:ieu-b-:	TRUE	reported	igd	2	TRUE	high	0.0001928	22.328655
|| id:ieu-b-:	TRUE	reported	igd	2	TRUE	high	0.0004131	47.860777
|| id:ieu-b-:	TRUE	reported	igd	2	TRUE	high	0.0001848	21.401407
|| id:ieu-b-:	TRUE	reported	igd	2	TRUE	high	0.000229	26.524973
|| id:ieu-b-:	TRUE	reported	igd	2	TRUE	high	0.0002763	32.002915
|| id:ieu-b-:	TRUE	reported	igd	2	TRUE	high	0.0002004	23.215345
|| id:ieu-b-:	TRUE	reported	igd	2	TRUE	high	0.0002583	29.914626

SNP	effect_allele	other_allele	effect_allele	other_allele	beta.exposu	beta.outcon	eaf.exposur	eaf.outcome
rs10048743	T	G	T	G	-0.231112	-0.1024	0.8588	0.818
rs10200680	T	C	T	C	-0.248461	0.183	0.1441	0.1683
rs10252453	A	G	A	G	0.19062	0.2285	0.167	0.2012
rs1078324	A	C	A	C	-0.71335	-0.1967	0.0497	0.04166
rs10844759	A	G	A	G	-0.139762	-0.0531	0.5805	0.6444
rs10849926	G	T	G	T	0.261365	-0.2005	0.9175	0.9165
rs10852562	C	T	C	T	-0.173953	0.145	0.7783	0.8032
rs10912578	G	A	G	A	-0.24686	-0.0281	0.6988	0.6847
rs1143679	A	G	A	G	0.582216	-0.0871	0.1312	0.1166
rs11618747	C	T	C	T	0.438255	0.5688	0.0507	0.03462
rs11699710	T	G	T	G	0.530628	3.037	0.0169	0.002462
rs11739881	T	C	T	C	0.29267	-0.4104	0.0736	0.06247
rs1198325	C	T	C	T	-0.127833	0.2172	0.496	0.4959
rs12094036	C	T	C	T	-0.328504	-0.305	0.0815	0.09162
rs12524498	T	G	T	G	-0.673345	0.2762	0.0109	0.01981
rs12679073	A	G	A	G	-0.150823	-0.0373	0.2803	0.2186
rs12711161	G	T	G	T	0.139762	0.0961	0.3002	0.3573
rs12753920	G	A	G	A	0.131028	0.2249	0.335	0.3965
rs13019891	T	G	T	G	-0.562119	0.0858	0.4513	0.4606
rs13136219	T	C	T	C	-0.174353	-0.2523	0.3797	0.3674
rs13152726	T	C	T	C	-0.157004	0.0568	0.7913	0.7264
rs13332649	G	A	G	A	-0.314711	0.0876	0.1978	0.2777
rs14312312	A	G	A	G	0.470004	0.092	0.0308	0.04284
rs14515608	C	T	C	T	0.500775	0.6006	0.0169	0.005827
rs1464446	T	G	T	G	-0.328504	0.0611	0.1789	0.1766
rs14710076	A	G	A	G	0.350657	0.0039	0.0487	0.08034
rs14798583	A	G	A	G	-0.18633	0.1466	0.2137	0.2129
rs15018063	T	C	T	C	0.928219	-1.0031	0.0169	0.00366
rs15072773	T	C	T	C	0.518794	-0.3179	0.0119	0.0124
rs1544581	T	G	T	G	0.127833	0.0911	0.5527	0.5547
rs17054615	G	A	G	A	-0.385662	-0.3981	0.0427	0.04006
rs1780813	T	C	T	C	-0.634878	-1.0168	0.0169	0.00876
rs17849501	T	C	T	C	0.81093	-0.4102	0.0596	0.03822
rs1833676	T	C	T	C	-0.223144	0.1562	0.1074	0.1144
rs1965998	G	T	G	T	0.162519	-0.0332	0.6978	0.6565
rs1990760	T	C	T	C	0.139262	0.0417	0.6054	0.5846
rs201060	G	A	G	A	0.262364	-0.0449	0.0696	0.03878
rs2288450	T	C	T	C	0.19062	-0.2576	0.1243	0.1524
rs2431697	C	T	C	T	-0.223144	-0.2407	0.4314	0.387
rs2459611	T	C	T	C	0.261365	0.3253	0.8748	0.9468
rs2573219	C	A	C	A	0.587787	0.3909	0.0865	0.08523
rs268124	T	C	T	C	0.18633	0.0634	0.7256	0.6253
rs34703115	C	T	C	T	-0.616186	0.2519	0.0328	0.03358
rs35000415	T	C	T	C	0.587787	0.2207	0.1004	0.1504
rs35251378	A	G	A	G	-0.235722	0.083	0.2694	0.2625
rs353608	G	A	G	A	0.18633	-0.0136	0.5477	0.5333

rs35429841	T	C	T	C	0.262364	0.063	0.0954	0.08284
rs3747093	A	G	A	G	0.262364	0.0308	0.2018	0.3118
rs3753232	T	C	T	C	-0.174353	-0.2736	0.1561	0.1138
rs3810409	A	G	A	G	0.329304	-0.1032	0.0457	0.09089
rs389884	G	A	G	A	0.928219	-0.2876	0.0736	0.0928
rs4082517	A	G	A	G	-0.198451	-0.3609	0.1163	0.0701
rs4274624	T	C	T	C	-0.559616	-0.0018	0.7684	0.7689
rs4282415	A	G	A	G	-0.139262	-0.1188	0.325	0.4177
rs4388254	T	C	T	C	0.378436	0.0619	0.0706	0.113
rs4661543	G	T	G	T	0.274437	-0.0343	0.8728	0.9328
rs4789169	G	A	G	A	0.162519	-0.0919	0.7376	0.8223
rs4916215	T	C	T	C	0.223144	0.1941	0.7455	0.8157
rs4954125	G	T	G	T	0.174353	-0.0635	0.674	0.8001
rs55849330	A	C	A	C	0.14842	0.0008	0.2942	0.354
rs57844307	A	G	A	G	0.277632	-0.2986	0.0815	0.01841
rs58688157	G	A	G	A	-0.223144	0.0636	0.2684	0.2061
rs58721818	T	C	T	C	0.65752	-0.0653	0.0249	0.01797
rs597808	G	A	G	A	-0.162519	-0.3185	0.5338	0.5883
rs62259783	A	G	A	G	-0.162519	-0.1418	0.3767	0.2465
rs6659932	C	A	C	A	-0.182322	0.1575	0.837	0.9133
rs6671847	A	G	A	G	0.198851	0.0121	0.4871	0.4542
rs6679677	A	C	A	C	0.336472	0.5606	0.0915	0.1468
rs6717904	A	G	A	G	0.139762	-0.0963	0.329	0.352
rs6773182	C	A	C	A	-0.127833	0.0971	0.4344	0.444
rs6889239	C	T	C	T	0.277632	-0.1025	0.2575	0.2859
rs6947963	T	G	T	G	-0.223144	0.1437	0.1501	0.1261
rs7068215	G	A	G	A	-0.127833	-0.0284	0.4095	0.3089
rs7097397	A	G	A	G	-0.18633	-0.0094	0.3956	0.3701
rs7099156	T	C	T	C	0.131028	0.0519	0.4334	0.3568
rs71366535	A	G	A	G	0.322084	-0.2648	0.0726	0.04301
rs7200786	G	A	G	A	-0.157004	-0.3003	0.5119	0.4868
rs725772	C	T	C	T	-0.131028	0.0888	0.5199	0.5053
rs73050535	T	C	T	C	-0.71335	-0.1151	0.0298	0.005095
rs73068668	A	G	A	G	-0.314711	-0.4047	0.0954	0.08273
rs73934025	A	C	A	C	-0.235722	0.3325	0.0825	0.0658
rs756505	T	G	T	G	-0.131028	0.0582	0.5189	0.4928
rs7768653	T	C	T	C	-0.207014	0.1402	0.5984	0.5327
rs7823055	T	G	T	G	-0.350657	0.08	0.5765	0.5684
rs7899626	T	C	T	C	0.182322	-0.0631	0.3628	0.3926
rs7941765	C	T	C	T	0.139262	-0.0724	0.498	0.5533
rs80315778	A	G	A	G	0.207014	0.4563	0.1223	0.0808
rs866417	C	T	C	T	-0.162519	0.0602	0.502	0.4947
rs924546	G	A	G	A	0.150823	0.2003	0.7823	0.809
rs9852014	G	A	G	A	0.620577	-0.175	0.0746	0.07968
rs9895436	A	G	A	G	0.139762	0.095	0.4235	0.4563
rs9942952	A	G	A	G	0.139762	-0.2575	0.3857	0.3466

remove	palindromic	ambiguous	id.outcome	chr	pos	se.outcome	samplesize.(	pval.outcom
FALSE	FALSE	FALSE	finn-b-I9_HY	2	213890232	0.1646		0.533801
FALSE	FALSE	FALSE	finn-b-I9_HY	2	223961877	0.1684		0.2772
FALSE	FALSE	FALSE	finn-b-I9_HY	7	92556186	0.158		0.1481
FALSE	FALSE	FALSE	finn-b-I9_HY	5	149202268	0.3251		0.5451
FALSE	FALSE	FALSE	finn-b-I9_HY	12	34129015	0.1324		0.688201
FALSE	FALSE	FALSE	finn-b-I9_HY	12	109638760	0.2304		0.3842
FALSE	FALSE	FALSE	finn-b-I9_HY	16	58329828	0.1588		0.361
FALSE	FALSE	FALSE	finn-b-I9_HY	1	173251856	0.137		0.8377
FALSE	FALSE	FALSE	finn-b-I9_HY	16	31276811	0.198		0.6599
FALSE	FALSE	FALSE	finn-b-I9_HY	13	82772038	0.3491		0.1032
FALSE	FALSE	FALSE	finn-b-I9_HY	20	57987383	1.4903		0.04157
FALSE	FALSE	FALSE	finn-b-I9_HY	13	20946829	0.2594		0.1135
FALSE	FALSE	FALSE	finn-b-I9_HY	13	50383685	0.1271		0.0873595
FALSE	FALSE	FALSE	finn-b-I9_HY	1	183558174	0.2177		0.1612
FALSE	FALSE	FALSE	finn-b-I9_HY	6	31444187	0.4636		0.5513
FALSE	FALSE	FALSE	finn-b-I9_HY	8	130222310	0.1545		0.8091
FALSE	FALSE	FALSE	finn-b-I9_HY	4	81128045	0.1321		0.4671
FALSE	FALSE	FALSE	finn-b-I9_HY	1	92665899	0.1294		0.0822697
FALSE	FALSE	FALSE	finn-b-I9_HY	2	113829869	0.1275		0.501
FALSE	FALSE	FALSE	finn-b-I9_HY	4	102743687	0.1318		0.0556404
FALSE	FALSE	FALSE	finn-b-I9_HY	4	9522682	0.143		0.691301
FALSE	FALSE	FALSE	finn-b-I9_HY	16	85966683	0.1413		0.535
FALSE	FALSE	FALSE	finn-b-I9_HY	17	38009000	0.3204		0.7739
FALSE	FALSE	FALSE	finn-b-I9_HY	3	21369907	0.9031		0.506001
FALSE	FALSE	FALSE	finn-b-I9_HY	3	146601295	0.1664		0.7136
FALSE	FALSE	FALSE	finn-b-I9_HY	8	108607014	0.2351		0.9866
FALSE	FALSE	FALSE	finn-b-I9_HY	5	36916867	0.1555		0.3459
FALSE	FALSE	FALSE	finn-b-I9_HY	6	31010047	1.0438		0.3366
FALSE	FALSE	FALSE	finn-b-I9_HY	7	73605165	0.591		0.5907
FALSE	FALSE	FALSE	finn-b-I9_HY	2	210641048	0.1275		0.4748
FALSE	FALSE	FALSE	finn-b-I9_HY	3	55008091	0.3313		0.2295
FALSE	FALSE	FALSE	finn-b-I9_HY	1	246444082	0.6748		0.1318
FALSE	FALSE	FALSE	finn-b-I9_HY	1	183542323	0.3268		0.2094
FALSE	FALSE	FALSE	finn-b-I9_HY	8	74143975	0.2019		0.439
FALSE	FALSE	FALSE	finn-b-I9_HY	3	159533769	0.1337		0.8037
FALSE	FALSE	FALSE	finn-b-I9_HY	2	163124051	0.1289		0.7462
FALSE	FALSE	FALSE	finn-b-I9_HY	6	6727398	0.3353		0.8935
FALSE	FALSE	FALSE	finn-b-I9_HY	19	41209177	0.1766		0.1447
FALSE	FALSE	FALSE	finn-b-I9_HY	5	159879978	0.1297		0.0634293
FALSE	FALSE	FALSE	finn-b-I9_HY	2	191939187	0.2861		0.2556
FALSE	FALSE	FALSE	finn-b-I9_HY	2	233288667	0.2302		0.0895097
FALSE	FALSE	FALSE	finn-b-I9_HY	2	65654364	0.131		0.6281
FALSE	FALSE	FALSE	finn-b-I9_HY	2	40282854	0.3627		0.4875
FALSE	FALSE	FALSE	finn-b-I9_HY	7	128585616	0.1764		0.2107
FALSE	FALSE	FALSE	finn-b-I9_HY	19	10459969	0.1446		0.5661
FALSE	FALSE	FALSE	finn-b-I9_HY	11	35101738	0.1269		0.9144

FALSE	FALSE	FALSE	finn-b-I9_HY	12	129273505	0.2297	0.7838
FALSE	FALSE	FALSE	finn-b-I9_HY	22	21984379	0.1368	0.822
FALSE	FALSE	FALSE	finn-b-I9_HY	1	24838097	0.2032	0.1782
FALSE	FALSE	FALSE	finn-b-I9_HY	19	2180515	0.2187	0.6368
FALSE	FALSE	FALSE	finn-b-I9_HY	6	31940897	0.2409	0.2326
FALSE	FALSE	FALSE	finn-b-I9_HY	10	71704566	0.2574	0.1609
FALSE	FALSE	FALSE	finn-b-I9_HY	2	191958656	0.151	0.9906
FALSE	FALSE	FALSE	finn-b-I9_HY	6	17006346	0.1286	0.3557
FALSE	FALSE	FALSE	finn-b-I9_HY	5	133428601	0.1998	0.7567
FALSE	FALSE	FALSE	finn-b-I9_HY	1	15229101	0.2582	0.8942
FALSE	FALSE	FALSE	finn-b-I9_HY	17	73324876	0.1675	0.5833
FALSE	FALSE	FALSE	finn-b-I9_HY	1	173314540	0.1648	0.2389
FALSE	FALSE	FALSE	finn-b-I9_HY	2	135046984	0.1596	0.690601
FALSE	FALSE	FALSE	finn-b-I9_HY	5	100200910	0.1317	0.995
FALSE	FALSE	FALSE	finn-b-I9_HY	6	34980945	0.4927	0.5444
FALSE	FALSE	FALSE	finn-b-I9_HY	11	625085	0.1583	0.687999
FALSE	FALSE	FALSE	finn-b-I9_HY	6	138243739	0.4878	0.8935
FALSE	FALSE	FALSE	finn-b-I9_HY	12	111973358	0.1293	0.0137699
FALSE	FALSE	FALSE	finn-b-I9_HY	3	58429135	0.1473	0.3356
FALSE	FALSE	FALSE	finn-b-I9_HY	1	67802371	0.229	0.491501
FALSE	FALSE	FALSE	finn-b-I9_HY	1	161478810	0.1277	0.9243
FALSE	FALSE	FALSE	finn-b-I9_HY	1	114303808	0.1797	0.001809
FALSE	FALSE	FALSE	finn-b-I9_HY	2	155643609	0.1346	0.4745
FALSE	FALSE	FALSE	finn-b-I9_HY	3	29378090	0.1273	0.4459
FALSE	FALSE	FALSE	finn-b-I9_HY	5	150457771	0.1409	0.4672
FALSE	FALSE	FALSE	finn-b-I9_HY	7	147918464	0.1908	0.4513
FALSE	FALSE	FALSE	finn-b-I9_HY	10	101859870	0.1377	0.8369
FALSE	FALSE	FALSE	finn-b-I9_HY	10	50025396	0.1317	0.943
FALSE	FALSE	FALSE	finn-b-I9_HY	10	61575320	0.1327	0.6959
FALSE	FALSE	FALSE	finn-b-I9_HY	18	77277478	0.3262	0.4169
FALSE	FALSE	FALSE	finn-b-I9_HY	16	11177801	0.1268	0.0178999
FALSE	FALSE	FALSE	finn-b-I9_HY	13	48403372	0.1272	0.4851
FALSE	FALSE	FALSE	finn-b-I9_HY	12	5012503	0.95	0.9036
FALSE	FALSE	FALSE	finn-b-I9_HY	19	55763262	0.2293	0.07758
FALSE	FALSE	FALSE	finn-b-I9_HY	19	50153686	0.2586	0.1985
FALSE	FALSE	FALSE	finn-b-I9_HY	22	27030951	0.1272	0.6475
FALSE	FALSE	FALSE	finn-b-I9_HY	6	106574794	0.1281	0.2737
FALSE	FALSE	FALSE	finn-b-I9_HY	8	55511676	0.1276	0.5305
FALSE	FALSE	FALSE	finn-b-I9_HY	10	63825561	0.1303	0.6284
FALSE	FALSE	FALSE	finn-b-I9_HY	11	128499000	0.1282	0.5723
FALSE	FALSE	FALSE	finn-b-I9_HY	11	11767584	0.2392	0.0564196
FALSE	FALSE	FALSE	finn-b-I9_HY	7	42121585	0.1273	0.636099
FALSE	FALSE	FALSE	finn-b-I9_HY	16	13046720	0.1621	0.2167
FALSE	FALSE	FALSE	finn-b-I9_HY	3	129084581	0.2321	0.4508
FALSE	FALSE	FALSE	finn-b-I9_HY	17	43457886	0.1269	0.4543
FALSE	FALSE	FALSE	finn-b-I9_HY	9	8993618	0.1346	0.0557494

outcome	originalnam	outcome.de	mr_keep.ou	data_source	proxy.outco	target_snp.c	proxy_snp.o	target_a1.ou
Hypertensio	Hypertensio	Hypertensio	TRUE	igd				
Hypertensio	Hypertensio	Hypertensio	TRUE	igd				
Hypertensio	Hypertensio	Hypertensio	TRUE	igd				
Hypertensio	Hypertensio	Hypertensio	TRUE	igd				
Hypertensio	Hypertensio	Hypertensio	TRUE	igd				
Hypertensio	Hypertensio	Hypertensio	TRUE	igd				
Hypertensio	Hypertensio	Hypertensio	TRUE	igd				
Hypertensio	Hypertensio	Hypertensio	TRUE	igd				
Hypertensio	Hypertensio	Hypertensio	TRUE	igd				
Hypertensio	Hypertensio	Hypertensio	TRUE	igd				
Hypertensio	Hypertensio	Hypertensio	TRUE	igd				
Hypertensio	Hypertensio	Hypertensio	TRUE	igd				
Hypertensio	Hypertensio	Hypertensio	TRUE	igd				
Hypertensio	Hypertensio	Hypertensio	TRUE	igd				
Hypertensio	Hypertensio	Hypertensio	TRUE	igd				
Hypertensio	Hypertensio	Hypertensio	TRUE	igd				
Hypertensio	Hypertensio	Hypertensio	TRUE	igd				
Hypertensio	Hypertensio	Hypertensio	TRUE	igd				
Hypertensio	Hypertensio	Hypertensio	TRUE	igd				
Hypertensio	Hypertensio	Hypertensio	TRUE	igd				
Hypertensio	Hypertensio	Hypertensio	TRUE	igd	TRUE	rs13152726	rs9715593	C
Hypertensio	Hypertensio	Hypertensio	TRUE	igd				
Hypertensio	Hypertensio	Hypertensio	TRUE	igd	TRUE	rs14312312	rs8068894	A
Hypertensio	Hypertensio	Hypertensio	TRUE	igd				
Hypertensio	Hypertensio	Hypertensio	TRUE	igd				
Hypertensio	Hypertensio	Hypertensio	TRUE	igd				
Hypertensio	Hypertensio	Hypertensio	TRUE	igd				
Hypertensio	Hypertensio	Hypertensio	TRUE	igd				
Hypertensio	Hypertensio	Hypertensio	TRUE	igd				
Hypertensio	Hypertensio	Hypertensio	TRUE	igd				
Hypertensio	Hypertensio	Hypertensio	TRUE	igd				
Hypertensio	Hypertensio	Hypertensio	TRUE	igd				
Hypertensio	Hypertensio	Hypertensio	TRUE	igd				
Hypertensio	Hypertensio	Hypertensio	TRUE	igd				
Hypertensio	Hypertensio	Hypertensio	TRUE	igd				
Hypertensio	Hypertensio	Hypertensio	TRUE	igd				
Hypertensio	Hypertensio	Hypertensio	TRUE	igd				
Hypertensio	Hypertensio	Hypertensio	TRUE	igd				
Hypertensio	Hypertensio	Hypertensio	TRUE	igd				
Hypertensio	Hypertensio	Hypertensio	TRUE	igd				
Hypertensio	Hypertensio	Hypertensio	TRUE	igd				
Hypertensio	Hypertensio	Hypertensio	TRUE	igd				
Hypertensio	Hypertensio	Hypertensio	TRUE	igd				
Hypertensio	Hypertensio	Hypertensio	TRUE	igd				
Hypertensio	Hypertensio	Hypertensio	TRUE	igd				
Hypertensio	Hypertensio	Hypertensio	TRUE	igd				

Hypertensio	Hypertensio	Hypertensio	TRUE	igd					
Hypertensio	Hypertensio	Hypertensio	TRUE	igd					
Hypertensio	Hypertensio	Hypertensio	TRUE	igd					
Hypertensio	Hypertensio	Hypertensio	TRUE	igd					
Hypertensio	Hypertensio	Hypertensio	TRUE	igd					
Hypertensio	Hypertensio	Hypertensio	TRUE	igd					
Hypertensio	Hypertensio	Hypertensio	TRUE	igd					
Hypertensio	Hypertensio	Hypertensio	TRUE	igd					
Hypertensio	Hypertensio	Hypertensio	TRUE	igd					
Hypertensio	Hypertensio	Hypertensio	TRUE	igd					
Hypertensio	Hypertensio	Hypertensio	TRUE	igd					
Hypertensio	Hypertensio	Hypertensio	TRUE	igd					
Hypertensio	Hypertensio	Hypertensio	TRUE	igd					
Hypertensio	Hypertensio	Hypertensio	TRUE	igd		TRUE	rs55849330	rs2059846	A
Hypertensio	Hypertensio	Hypertensio	TRUE	igd					
Hypertensio	Hypertensio	Hypertensio	TRUE	igd					
Hypertensio	Hypertensio	Hypertensio	TRUE	igd					
Hypertensio	Hypertensio	Hypertensio	TRUE	igd					
Hypertensio	Hypertensio	Hypertensio	TRUE	igd					
Hypertensio	Hypertensio	Hypertensio	TRUE	igd					
Hypertensio	Hypertensio	Hypertensio	TRUE	igd					
Hypertensio	Hypertensio	Hypertensio	TRUE	igd					
Hypertensio	Hypertensio	Hypertensio	TRUE	igd					
Hypertensio	Hypertensio	Hypertensio	TRUE	igd					
Hypertensio	Hypertensio	Hypertensio	TRUE	igd					
Hypertensio	Hypertensio	Hypertensio	TRUE	igd					
Hypertensio	Hypertensio	Hypertensio	TRUE	igd					
Hypertensio	Hypertensio	Hypertensio	TRUE	igd					
Hypertensio	Hypertensio	Hypertensio	TRUE	igd					
Hypertensio	Hypertensio	Hypertensio	TRUE	igd					
Hypertensio	Hypertensio	Hypertensio	TRUE	igd					
Hypertensio	Hypertensio	Hypertensio	TRUE	igd					
Hypertensio	Hypertensio	Hypertensio	TRUE	igd					
Hypertensio	Hypertensio	Hypertensio	TRUE	igd					
Hypertensio	Hypertensio	Hypertensio	TRUE	igd					
Hypertensio	Hypertensio	Hypertensio	TRUE	igd					
Hypertensio	Hypertensio	Hypertensio	TRUE	igd					
Hypertensio	Hypertensio	Hypertensio	TRUE	igd					
Hypertensio	Hypertensio	Hypertensio	TRUE	igd					
Hypertensio	Hypertensio	Hypertensio	TRUE	igd					
Hypertensio	Hypertensio	Hypertensio	TRUE	igd					
Hypertensio	Hypertensio	Hypertensio	TRUE	igd					
Hypertensio	Hypertensio	Hypertensio	TRUE	igd					
Hypertensio	Hypertensio	Hypertensio	TRUE	igd					
Hypertensio	Hypertensio	Hypertensio	TRUE	igd					
Hypertensio	Hypertensio	Hypertensio	TRUE	igd					

target_a2.ou	proxy_a1.ou	proxy_a2.ou	pos.exposur	chr.exposur	samplesize.e	pval.exposu	se.exposure	id.exposure
			213890232	2	14267	2.038E-08	0.0412056	ebi-a-GCST0
			223961877	2	14267	4.963E-09	0.0424835	ebi-a-GCST0
			92556186	7	14267	3.007E-06	0.0408151	ebi-a-GCST0
			149202268	5	14267	7.106E-20	0.0781665	ebi-a-GCST0
			34129015	12	14267	7.648E-06	0.0312331	ebi-a-GCST0
			109638760	12	14267	1.655E-06	0.0545472	ebi-a-GCST0
			58329828	16	14267	1.992E-07	0.0334521	ebi-a-GCST0
			173251856	1	14267	1.648E-15	0.0309918	ebi-a-GCST0
			31276811	16	14267	5.027E-48	0.0399866	ebi-a-GCST0
			82772038	13	14267	1.466E-06	0.0910039	ebi-a-GCST0
			57987383	20	14267	6.716E-06	0.117852	ebi-a-GCST0
			20946829	13	14267	1.604E-06	0.0610008	ebi-a-GCST0
			50383685	13	14267	2.478E-06	0.0271413	ebi-a-GCST0
			183558174	1	14267	1.366E-08	0.0578595	ebi-a-GCST0
			31444187	6	14267	2.484E-08	0.120793	ebi-a-GCST0
			130222310	8	14267	3.169E-06	0.0323687	ebi-a-GCST0
			81128045	4	14267	9.009E-06	0.0314802	ebi-a-GCST0
			92665899	1	14267	9.35E-06	0.0295662	ebi-a-GCST0
			113829869	2	14267	1.647E-83	0.0290336	ebi-a-GCST0
			102743687	4	14267	3.504E-10	0.027787	ebi-a-GCST0
T	T	G	9520869	4	14267	6.849E-06	0.0349028	ebi-a-GCST0
			85966683	16	14267	5.428E-17	0.0375683	ebi-a-GCST0
G	G	A	38007190	17	14267	2.232E-08	0.0840342	ebi-a-GCST0
			21369907	3	14267	1.567E-06	0.104274	ebi-a-GCST0
			146601295	3	14267	2.793E-16	0.0401497	ebi-a-GCST0
			108607014	8	14267	1.212E-06	0.0722454	ebi-a-GCST0
			36916867	5	14267	1.907E-06	0.0391203	ebi-a-GCST0
			31010047	6	14267	2.661E-41	0.0689573	ebi-a-GCST0
			73605165	7	14267	1.067E-06	0.106336	ebi-a-GCST0
			210641048	2	14267	6.348E-06	0.0283165	ebi-a-GCST0
			55008091	3	14267	5.824E-06	0.0850851	ebi-a-GCST0
			246444082	1	14267	8.357E-07	0.128861	ebi-a-GCST0
			183542323	1	14267	1.814E-59	0.0498642	ebi-a-GCST0
			74143975	8	14267	2.354E-06	0.0472722	ebi-a-GCST0
			159533769	3	14267	1.605E-07	0.0310145	ebi-a-GCST0
			163124051	2	14267	2.476E-06	0.0295668	ebi-a-GCST0
			6727398	6	14267	9.022E-06	0.0590993	ebi-a-GCST0
			41209177	19	14267	4.428E-06	0.0415275	ebi-a-GCST0
			159879978	5	14267	2.601E-14	0.0292964	ebi-a-GCST0
			191939187	2	14267	7.62E-09	0.045245	ebi-a-GCST0
			233288667	2	14267	1.133E-42	0.0429292	ebi-a-GCST0
			65654364	2	14267	8.603E-09	0.0323703	ebi-a-GCST0
			40282854	2	14267	4.081E-09	0.104778	ebi-a-GCST0
			128585616	7	14267	1.861E-45	0.041539	ebi-a-GCST0
			10459969	19	14267	3.61E-13	0.0324266	ebi-a-GCST0
			35101738	11	14267	2.932E-11	0.0280198	ebi-a-GCST0

			129273505	12	14267	6.476E-08	0.0485398	ebi-a-GCST0
			21984379	22	14267	2.881E-14	0.0345055	ebi-a-GCST0
			24838097	1	14267	6.922E-06	0.0387789	ebi-a-GCST0
			2180515	19	14267	3.791E-06	0.0712387	ebi-a-GCST0
			31940897	6	14267	2.92E-102	0.0432319	ebi-a-GCST0
			71704566	10	14267	2.162E-06	0.0418879	ebi-a-GCST0
			191958656	2	14267	9.732E-66	0.0326791	ebi-a-GCST0
			17006346	6	14267	4.344E-06	0.0303125	ebi-a-GCST0
			133428601	5	14267	3.71E-10	0.0603977	ebi-a-GCST0
			15229101	1	14267	9.399E-11	0.0423755	ebi-a-GCST0
			73324876	17	14267	1.343E-06	0.0336253	ebi-a-GCST0
			173314540	1	14267	5.066E-11	0.0339693	ebi-a-GCST0
			135046984	2	14267	8.804E-08	0.0325905	ebi-a-GCST0
C	C	T	100184647	5	14267	8.409E-07	0.0301322	ebi-a-GCST0
			34980945	6	14267	4.974E-08	0.0509208	ebi-a-GCST0
			625085	11	14267	2.968E-11	0.0335647	ebi-a-GCST0
			138243739	6	14267	3.377E-18	0.0755941	ebi-a-GCST0
			111973358	12	14267	3.507E-08	0.0294736	ebi-a-GCST0
			58429135	3	14267	8.281E-08	0.0303156	ebi-a-GCST0
			67802371	1	14267	1.509E-06	0.0379045	ebi-a-GCST0
			161478810	1	14267	6.64E-12	0.0289651	ebi-a-GCST0
			114303808	1	14267	4.546E-13	0.0464854	ebi-a-GCST0
			155643609	2	14267	8.438E-06	0.0313806	ebi-a-GCST0
			29378090	3	14267	6.874E-06	0.0284228	ebi-a-GCST0
			150457771	5	14267	2.19E-18	0.03174	ebi-a-GCST0
			147918464	7	14267	1.421E-07	0.0424026	ebi-a-GCST0
			101859870	10	14267	9.631E-06	0.0288869	ebi-a-GCST0
			50025396	10	14267	8.604E-11	0.0287118	ebi-a-GCST0
			61575320	10	14267	9.208E-06	0.0295442	ebi-a-GCST0
			77277478	18	14267	6.082E-06	0.0712021	ebi-a-GCST0
			11177801	16	14267	7.206E-08	0.0291506	ebi-a-GCST0
			48403372	13	14267	6.698E-06	0.0290975	ebi-a-GCST0
			5012503	12	14267	9.105E-09	0.124134	ebi-a-GCST0
			55763262	19	14267	4.396E-08	0.0574903	ebi-a-GCST0
			50153686	19	14267	5.391E-06	0.0518188	ebi-a-GCST0
			27030951	22	14267	1.404E-06	0.027159	ebi-a-GCST0
			106574794	6	14267	3.108E-12	0.0296891	ebi-a-GCST0
			55511676	8	14267	1.643E-34	0.0286208	ebi-a-GCST0
			63825561	10	14267	4.186E-08	0.0332532	ebi-a-GCST0
			128499000	11	14267	1.136E-06	0.028617	ebi-a-GCST0
			11767584	11	14267	8.138E-07	0.0419735	ebi-a-GCST0
			42121585	7	14267	5.754E-08	0.0299504	ebi-a-GCST0
			13046720	16	14267	6.122E-06	0.0333522	ebi-a-GCST0
			129084581	3	14267	2.257E-36	0.0492727	ebi-a-GCST0
			43457886	17	14267	1.223E-06	0.0288053	ebi-a-GCST0
			8993618	9	14267	8.332E-06	0.0313616	ebi-a-GCST0

<table>
<tr><th>exposure</th><th>mr_keep.ex</th><th>pval_origin.</th><th>data_source</th><th>action</th><th>mr_keep</th><th>reliability.ex</th><th>R2</th><th>F</th></tr>
<tr><td>|| id:ebi-a-(</td><td>TRUE</td><td>reported</td><td>igd</td><td>2</td><td>TRUE</td><td>high</td><td>0.0022001</td><td>31.453691</td></tr>
<tr><td>|| id:ebi-a-(</td><td>TRUE</td><td>reported</td><td>igd</td><td>2</td><td>TRUE</td><td>high</td><td>0.0023917</td><td>34.199125</td></tr>
<tr><td>|| id:ebi-a-(</td><td>TRUE</td><td>reported</td><td>igd</td><td>2</td><td>TRUE</td><td>high</td><td>0.0015265</td><td>21.808928</td></tr>
<tr><td>|| id:ebi-a-(</td><td>TRUE</td><td>reported</td><td>igd</td><td>2</td><td>TRUE</td><td>high</td><td>0.0058037</td><td>83.27279</td></tr>
<tr><td>|| id:ebi-a-(</td><td>TRUE</td><td>reported</td><td>igd</td><td>2</td><td>TRUE</td><td>high</td><td>0.0014015</td><td>20.021064</td></tr>
<tr><td>|| id:ebi-a-(</td><td>TRUE</td><td>reported</td><td>igd</td><td>2</td><td>TRUE</td><td>high</td><td>0.0016066</td><td>22.955621</td></tr>
<tr><td>|| id:ebi-a-(</td><td>TRUE</td><td>reported</td><td>igd</td><td>2</td><td>TRUE</td><td>high</td><td>0.0018917</td><td>27.036856</td></tr>
<tr><td>|| id:ebi-a-(</td><td>TRUE</td><td>reported</td><td>igd</td><td>2</td><td>TRUE</td><td>high</td><td>0.0044274</td><td>63.437632</td></tr>
<tr><td>|| id:ebi-a-(</td><td>TRUE</td><td>reported</td><td>igd</td><td>2</td><td>TRUE</td><td>high</td><td>0.014642</td><td>211.97197</td></tr>
<tr><td>|| id:ebi-a-(</td><td>TRUE</td><td>reported</td><td>igd</td><td>2</td><td>TRUE</td><td>high</td><td>0.0016229</td><td>23.188511</td></tr>
<tr><td>|| id:ebi-a-(</td><td>TRUE</td><td>reported</td><td>igd</td><td>2</td><td>TRUE</td><td>high</td><td>0.0014189</td><td>20.269616</td></tr>
<tr><td>|| id:ebi-a-(</td><td>TRUE</td><td>reported</td><td>igd</td><td>2</td><td>TRUE</td><td>high</td><td>0.0016108</td><td>23.015715</td></tr>
<tr><td>|| id:ebi-a-(</td><td>TRUE</td><td>reported</td><td>igd</td><td>2</td><td>TRUE</td><td>high</td><td>0.0015524</td><td>22.180115</td></tr>
<tr><td>|| id:ebi-a-(</td><td>TRUE</td><td>reported</td><td>igd</td><td>2</td><td>TRUE</td><td>high</td><td>0.0022543</td><td>32.2308</td></tr>
<tr><td>|| id:ebi-a-(</td><td>TRUE</td><td>reported</td><td>igd</td><td>2</td><td>TRUE</td><td>high</td><td>0.0021733</td><td>31.069256</td></tr>
<tr><td>|| id:ebi-a-(</td><td>TRUE</td><td>reported</td><td>igd</td><td>2</td><td>TRUE</td><td>high</td><td>0.0015195</td><td>21.708197</td></tr>
<tr><td>|| id:ebi-a-(</td><td>TRUE</td><td>reported</td><td>igd</td><td>2</td><td>TRUE</td><td>high</td><td>0.0013797</td><td>19.707991</td></tr>
<tr><td>|| id:ebi-a-(</td><td>TRUE</td><td>reported</td><td>igd</td><td>2</td><td>TRUE</td><td>high</td><td>0.0013747</td><td>19.637053</td></tr>
<tr><td>|| id:ebi-a-(</td><td>TRUE</td><td>reported</td><td>igd</td><td>2</td><td>TRUE</td><td>high</td><td>0.0256011</td><td>374.79507</td></tr>
<tr><td>|| id:ebi-a-(</td><td>TRUE</td><td>reported</td><td>igd</td><td>2</td><td>TRUE</td><td>high</td><td>0.002752</td><td>39.365398</td></tr>
<tr><td>|| id:ebi-a-(</td><td>TRUE</td><td>reported</td><td>igd</td><td>2</td><td>TRUE</td><td>high</td><td>0.0014163</td><td>20.232056</td></tr>
<tr><td>|| id:ebi-a-(</td><td>TRUE</td><td>reported</td><td>igd</td><td>2</td><td>TRUE</td><td>high</td><td>0.0048946</td><td>70.164894</td></tr>
<tr><td>|| id:ebi-a-(</td><td>TRUE</td><td>reported</td><td>igd</td><td>2</td><td>TRUE</td><td>high</td><td>0.0021878</td><td>31.27736</td></tr>
<tr><td>|| id:ebi-a-(</td><td>TRUE</td><td>reported</td><td>igd</td><td>2</td><td>TRUE</td><td>high</td><td>0.001614</td><td>23.060692</td></tr>
<tr><td>|| id:ebi-a-(</td><td>TRUE</td><td>reported</td><td>igd</td><td>2</td><td>TRUE</td><td>high</td><td>0.0046704</td><td>66.935395</td></tr>
<tr><td>|| id:ebi-a-(</td><td>TRUE</td><td>reported</td><td>igd</td><td>2</td><td>TRUE</td><td>high</td><td>0.0016485</td><td>23.555034</td></tr>
<tr><td>|| id:ebi-a-(</td><td>TRUE</td><td>reported</td><td>igd</td><td>2</td><td>TRUE</td><td>high</td><td>0.0015876</td><td>22.682991</td></tr>
<tr><td>|| id:ebi-a-(</td><td>TRUE</td><td>reported</td><td>igd</td><td>2</td><td>TRUE</td><td>high</td><td>0.0125408</td><td>181.16718</td></tr>
<tr><td>|| id:ebi-a-(</td><td>TRUE</td><td>reported</td><td>igd</td><td>2</td><td>TRUE</td><td>high</td><td>0.0016656</td><td>23.799529</td></tr>
<tr><td>|| id:ebi-a-(</td><td>TRUE</td><td>reported</td><td>igd</td><td>2</td><td>TRUE</td><td>high</td><td>0.0014264</td><td>20.377267</td></tr>
<tr><td>|| id:ebi-a-(</td><td>TRUE</td><td>reported</td><td>igd</td><td>2</td><td>TRUE</td><td>high</td><td>0.001438</td><td>20.542145</td></tr>
<tr><td>|| id:ebi-a-(</td><td>TRUE</td><td>reported</td><td>igd</td><td>2</td><td>TRUE</td><td>high</td><td>0.0016985</td><td>24.270386</td></tr>
<tr><td>|| id:ebi-a-(</td><td>TRUE</td><td>reported</td><td>igd</td><td>2</td><td>TRUE</td><td>high</td><td>0.0182003</td><td>264.4406</td></tr>
<tr><td>|| id:ebi-a-(</td><td>TRUE</td><td>reported</td><td>igd</td><td>2</td><td>TRUE</td><td>high</td><td>0.0015594</td><td>22.279114</td></tr>
<tr><td>|| id:ebi-a-(</td><td>TRUE</td><td>reported</td><td>igd</td><td>2</td><td>TRUE</td><td>high</td><td>0.0019209</td><td>27.454771</td></tr>
<tr><td>|| id:ebi-a-(</td><td>TRUE</td><td>reported</td><td>igd</td><td>2</td><td>TRUE</td><td>high</td><td>0.0015526</td><td>22.181746</td></tr>
<tr><td>|| id:ebi-a-(</td><td>TRUE</td><td>reported</td><td>igd</td><td>2</td><td>TRUE</td><td>high</td><td>0.0013795</td><td>19.705294</td></tr>
<tr><td>|| id:ebi-a-(</td><td>TRUE</td><td>reported</td><td>igd</td><td>2</td><td>TRUE</td><td>high</td><td>0.0014747</td><td>21.067086</td></tr>
<tr><td>|| id:ebi-a-(</td><td>TRUE</td><td>reported</td><td>igd</td><td>2</td><td>TRUE</td><td>high</td><td>0.0040499</td><td>58.007083</td></tr>
<tr><td>|| id:ebi-a-(</td><td>TRUE</td><td>reported</td><td>igd</td><td>2</td><td>TRUE</td><td>high</td><td>0.0023335</td><td>33.365128</td></tr>
<tr><td>|| id:ebi-a-(</td><td>TRUE</td><td>reported</td><td>igd</td><td>2</td><td>TRUE</td><td>high</td><td>0.0129698</td><td>187.44483</td></tr>
<tr><td>|| id:ebi-a-(</td><td>TRUE</td><td>reported</td><td>igd</td><td>2</td><td>TRUE</td><td>high</td><td>0.002317</td><td>33.129222</td></tr>
<tr><td>|| id:ebi-a-(</td><td>TRUE</td><td>reported</td><td>igd</td><td>2</td><td>TRUE</td><td>high</td><td>0.0024182</td><td>34.579807</td></tr>
<tr><td>|| id:ebi-a-(</td><td>TRUE</td><td>reported</td><td>igd</td><td>2</td><td>TRUE</td><td>high</td><td>0.0138402</td><td>200.20135</td></tr>
<tr><td>|| id:ebi-a-(</td><td>TRUE</td><td>reported</td><td>igd</td><td>2</td><td>TRUE</td><td>high</td><td>0.0036903</td><td>52.836802</td></tr>
<tr><td>|| id:ebi-a-(</td><td>TRUE</td><td>reported</td><td>igd</td><td>2</td><td>TRUE</td><td>high</td><td>0.00309</td><td>44.215508</td></tr>
</table>

<table>
<tr><td>| | id:ebi-a-(</td><td>TRUE</td><td>reported</td><td>igd</td><td></td><td>2</td><td>TRUE</td><td>high</td><td>0.0020436</td><td>29.211351</td></tr>
<tr><td>| | id:ebi-a-(</td><td>TRUE</td><td>reported</td><td>igd</td><td></td><td>2</td><td>TRUE</td><td>high</td><td>0.0040359</td><td>57.805738</td></tr>
<tr><td>| | id:ebi-a-(</td><td>TRUE</td><td>reported</td><td>igd</td><td></td><td>2</td><td>TRUE</td><td>high</td><td>0.0014149</td><td>20.211893</td></tr>
<tr><td>| | id:ebi-a-(</td><td>TRUE</td><td>reported</td><td>igd</td><td></td><td>2</td><td>TRUE</td><td>high</td><td>0.0014955</td><td>21.364914</td></tr>
<tr><td>| | id:ebi-a-(</td><td>TRUE</td><td>reported</td><td>igd</td><td></td><td>2</td><td>TRUE</td><td>high</td><td>0.0313003</td><td>460.92618</td></tr>
<tr><td>| | id:ebi-a-(</td><td>TRUE</td><td>reported</td><td>igd</td><td></td><td>2</td><td>TRUE</td><td>high</td><td>0.0015708</td><td>22.44236</td></tr>
<tr><td>| | id:ebi-a-(</td><td>TRUE</td><td>reported</td><td>igd</td><td></td><td>2</td><td>TRUE</td><td>high</td><td>0.0201405</td><td>293.21028</td></tr>
<tr><td>| | id:ebi-a-(</td><td>TRUE</td><td>reported</td><td>igd</td><td></td><td>2</td><td>TRUE</td><td>high</td><td>0.0014772</td><td>21.10381</td></tr>
<tr><td>| | id:ebi-a-(</td><td>TRUE</td><td>reported</td><td>igd</td><td></td><td>2</td><td>TRUE</td><td>high</td><td>0.0027442</td><td>39.253935</td></tr>
<tr><td>| | id:ebi-a-(</td><td>TRUE</td><td>reported</td><td>igd</td><td></td><td>2</td><td>TRUE</td><td>high</td><td>0.0029312</td><td>41.93675</td></tr>
<tr><td>| | id:ebi-a-(</td><td>TRUE</td><td>reported</td><td>igd</td><td></td><td>2</td><td>TRUE</td><td>high</td><td>0.0016347</td><td>23.356893</td></tr>
<tr><td>| | id:ebi-a-(</td><td>TRUE</td><td>reported</td><td>igd</td><td></td><td>2</td><td>TRUE</td><td>high</td><td>0.0030155</td><td>43.145584</td></tr>
<tr><td>| | id:ebi-a-(</td><td>TRUE</td><td>reported</td><td>igd</td><td></td><td>2</td><td>TRUE</td><td>high</td><td>0.002002</td><td>28.616461</td></tr>
<tr><td>| | id:ebi-a-(</td><td>TRUE</td><td>reported</td><td>igd</td><td></td><td>2</td><td>TRUE</td><td>high</td><td>0.0016977</td><td>24.258407</td></tr>
<tr><td>| | id:ebi-a-(</td><td>TRUE</td><td>reported</td><td>igd</td><td></td><td>2</td><td>TRUE</td><td>high</td><td>0.0020793</td><td>29.722663</td></tr>
<tr><td>| | id:ebi-a-(</td><td>TRUE</td><td>reported</td><td>igd</td><td></td><td>2</td><td>TRUE</td><td>high</td><td>0.0030884</td><td>44.192035</td></tr>
<tr><td>| | id:ebi-a-(</td><td>TRUE</td><td>reported</td><td>igd</td><td></td><td>2</td><td>TRUE</td><td>high</td><td>0.0052749</td><td>75.645178</td></tr>
<tr><td>| | id:ebi-a-(</td><td>TRUE</td><td>reported</td><td>igd</td><td></td><td>2</td><td>TRUE</td><td>high</td><td>0.0021266</td><td>30.400521</td></tr>
<tr><td>| | id:ebi-a-(</td><td>TRUE</td><td>reported</td><td>igd</td><td></td><td>2</td><td>TRUE</td><td>high</td><td>0.0020103</td><td>28.735255</td></tr>
<tr><td>| | id:ebi-a-(</td><td>TRUE</td><td>reported</td><td>igd</td><td></td><td>2</td><td>TRUE</td><td>high</td><td>0.001619</td><td>23.133201</td></tr>
<tr><td>| | id:ebi-a-(</td><td>TRUE</td><td>reported</td><td>igd</td><td></td><td>2</td><td>TRUE</td><td>high</td><td>0.0032926</td><td>47.124267</td></tr>
<tr><td>| | id:ebi-a-(</td><td>TRUE</td><td>reported</td><td>igd</td><td></td><td>2</td><td>TRUE</td><td>high</td><td>0.0036588</td><td>52.384624</td></tr>
<tr><td>| | id:ebi-a-(</td><td>TRUE</td><td>reported</td><td>igd</td><td></td><td>2</td><td>TRUE</td><td>high</td><td>0.0013884</td><td>19.833294</td></tr>
<tr><td>| | id:ebi-a-(</td><td>TRUE</td><td>reported</td><td>igd</td><td></td><td>2</td><td>TRUE</td><td>high</td><td>0.0014158</td><td>20.225132</td></tr>
<tr><td>| | id:ebi-a-(</td><td>TRUE</td><td>reported</td><td>igd</td><td></td><td>2</td><td>TRUE</td><td>high</td><td>0.0053342</td><td>76.500507</td></tr>
<tr><td>| | id:ebi-a-(</td><td>TRUE</td><td>reported</td><td>igd</td><td></td><td>2</td><td>TRUE</td><td>high</td><td>0.0019374</td><td>27.690103</td></tr>
<tr><td>| | id:ebi-a-(</td><td>TRUE</td><td>reported</td><td>igd</td><td></td><td>2</td><td>TRUE</td><td>high</td><td>0.0013707</td><td>19.580474</td></tr>
<tr><td>| | id:ebi-a-(</td><td>TRUE</td><td>reported</td><td>igd</td><td></td><td>2</td><td>TRUE</td><td>high</td><td>0.0029433</td><td>42.109864</td></tr>
<tr><td>| | id:ebi-a-(</td><td>TRUE</td><td>reported</td><td>igd</td><td></td><td>2</td><td>TRUE</td><td>high</td><td>0.0013767</td><td>19.666309</td></tr>
<tr><td>| | id:ebi-a-(</td><td>TRUE</td><td>reported</td><td>igd</td><td></td><td>2</td><td>TRUE</td><td>high</td><td>0.0014322</td><td>20.459349</td></tr>
<tr><td>| | id:ebi-a-(</td><td>TRUE</td><td>reported</td><td>igd</td><td></td><td>2</td><td>TRUE</td><td>high</td><td>0.0020291</td><td>29.004511</td></tr>
<tr><td>| | id:ebi-a-(</td><td>TRUE</td><td>reported</td><td>igd</td><td></td><td>2</td><td>TRUE</td><td>high</td><td>0.0014193</td><td>20.274772</td></tr>
<tr><td>| | id:ebi-a-(</td><td>TRUE</td><td>reported</td><td>igd</td><td></td><td>2</td><td>TRUE</td><td>high</td><td>0.0023093</td><td>33.018926</td></tr>
<tr><td>| | id:ebi-a-(</td><td>TRUE</td><td>reported</td><td>igd</td><td></td><td>2</td><td>TRUE</td><td>high</td><td>0.002096</td><td>29.962208</td></tr>
<tr><td>| | id:ebi-a-(</td><td>TRUE</td><td>reported</td><td>igd</td><td></td><td>2</td><td>TRUE</td><td>high</td><td>0.0014483</td><td>20.690198</td></tr>
<tr><td>| | id:ebi-a-(</td><td>TRUE</td><td>reported</td><td>igd</td><td></td><td>2</td><td>TRUE</td><td>high</td><td>0.0016288</td><td>23.272326</td></tr>
<tr><td>| | id:ebi-a-(</td><td>TRUE</td><td>reported</td><td>igd</td><td></td><td>2</td><td>TRUE</td><td>high</td><td>0.0033962</td><td>48.612111</td></tr>
<tr><td>| | id:ebi-a-(</td><td>TRUE</td><td>reported</td><td>igd</td><td></td><td>2</td><td>TRUE</td><td>high</td><td>0.0104117</td><td>150.08615</td></tr>
<tr><td>| | id:ebi-a-(</td><td>TRUE</td><td>reported</td><td>igd</td><td></td><td>2</td><td>TRUE</td><td>high</td><td>0.0021026</td><td>30.057329</td></tr>
<tr><td>| | id:ebi-a-(</td><td>TRUE</td><td>reported</td><td>igd</td><td></td><td>2</td><td>TRUE</td><td>high</td><td>0.0016572</td><td>23.678608</td></tr>
<tr><td>| | id:ebi-a-(</td><td>TRUE</td><td>reported</td><td>igd</td><td></td><td>2</td><td>TRUE</td><td>high</td><td>0.0017021</td><td>24.321378</td></tr>
<tr><td>| | id:ebi-a-(</td><td>TRUE</td><td>reported</td><td>igd</td><td></td><td>2</td><td>TRUE</td><td>high</td><td>0.0020596</td><td>29.440294</td></tr>
<tr><td>| | id:ebi-a-(</td><td>TRUE</td><td>reported</td><td>igd</td><td></td><td>2</td><td>TRUE</td><td>high</td><td>0.0014313</td><td>20.446797</td></tr>
<tr><td>| | id:ebi-a-(</td><td>TRUE</td><td>reported</td><td>igd</td><td></td><td>2</td><td>TRUE</td><td>high</td><td>0.0109962</td><td>158.60532</td></tr>
<tr><td>| | id:ebi-a-(</td><td>TRUE</td><td>reported</td><td>igd</td><td></td><td>2</td><td>TRUE</td><td>high</td><td>0.0016473</td><td>23.538161</td></tr>
<tr><td>| | id:ebi-a-(</td><td>TRUE</td><td>reported</td><td>igd</td><td></td><td>2</td><td>TRUE</td><td>high</td><td>0.0013901</td><td>19.857332</td></tr>
</table>

SNP	effect_allele	other_allele	effect_allele	other_allele	beta.exposu	beta.outcon	eaf.exposure	eaf.outcome
rs10012242	G	A	G	A	0.1877	-0.1338	0.1223	0.1221
rs10091521	G	A	G	A	0.4538	0.3158	0.0159	0.03903
rs10128558	G	C	G	C	0.1639	0.195	0.1044	0.08927
rs10183097	C	T	C	T	0.2053	0.12	0.1451	0.1517
rs1027769	T	G	T	G	-0.9962	0.1916	0.9871	0.9765
rs1049371	G	A	G	A	0.1583	0.3464	0.159	0.1931
rs10760335	G	A	G	A	0.1357	-0.0738	0.332	0.3096
rs10774624	A	G	A	G	-0.2556	-0.321	0.5298	0.5996
rs10830227	A	G	A	G	0.1582	0.1046	0.5835	0.509
rs10852506	C	T	C	T	0.1194	0.0106	0.4155	0.4283
rs10865468	C	G	C	G	-0.1624	0.0577	0.2594	0.3103
rs10911399	G	A	G	A	-0.3707	-0.1339	0.0318	0.02724
rs11079322	T	C	T	C	-0.1269	0.0615	0.7753	0.7003
rs11125873	T	A	T	A	0.5692	-0.1092	0.0139	0.03002
rs11139960	G	T	G	T	0.4369	0.1164	0.0288	0.07606
rs11203203	A	G	A	G	0.116	0.0448	0.3519	0.2804
rs11216968	A	G	A	G	0.4873	-0.7146	0.0209	0.0127
rs11230837	C	G	C	G	0.3754	-0.7245	0.0219	0.01701
rs11259536	G	A	G	A	-0.1375	-0.1058	0.2097	0.2683
rs11278923	G	A	G	A	0.3728	-0.0157	0.0239	0.02026
rs11340327	T	A	T	A	0.4406	0.4274	0.0109	0.0142
rs11415735	G	A	G	A	0.413	-1.0266	0.0109	0.006091
rs11555655	T	G	T	G	0.2532	-0.2341	0.0765	0.02687
rs11570370	T	C	T	C	0.3293	0.491	0.0288	0.06782
rs11571297	C	T	C	T	-0.1964	0.0018	0.499	0.3719
rs1159619	A	C	A	C	0.1087	0.0654	0.4543	0.4406
rs11596750	C	T	C	T	-0.1208	0.0988	0.7117	0.6985
rs11704567	G	C	G	C	-0.3523	0.22	0.0268	0.01702
rs11717237	T	C	T	C	-0.1174	-0.1152	0.3608	0.3297
rs11720579	T	C	T	C	-0.8577	-0.0562	0.0139	0.008704
rs11726764	T	C	T	C	0.5569	1.0765	0.0139	0.002121
rs11727369	A	G	A	G	0.1153	0.0572	0.3767	0.4674
rs11761417	G	T	G	T	0.434	-0.3546	0.0189	0.006851
rs11774861	T	C	T	C	0.3926	-0.0972	0.0189	0.02165
rs11901096	A	G	A	G	0.2047	-0.0775	0.1113	0.08204
rs11930486	T	A	T	A	0.1377	0.0072	0.3151	0.305
rs11989229	C	T	C	T	-0.1216	-0.1779	0.3996	0.3027
rs12082589	A	C	A	C	0.3005	-0.3707	0.0358	0.031
rs12123737	G	A	G	A	0.1082	0.0904	0.4274	0.5361
rs12352863	C	G	C	G	-0.1461	-0.0342	0.174	0.153
rs12542501	A	G	A	G	-0.1818	0.1053	0.1491	0.143
rs12697021	A	G	A	G	0.1167	-0.2059	0.3469	0.356
rs12712117	T	C	T	C	-0.1168	-0.1795	0.3529	0.1781
rs12722495	C	T	C	T	-0.3145	0.2396	0.0775	0.0646
rs12804463	T	C	T	C	0.2089	0.1381	0.1113	0.1217
rs12907749	G	T	G	T	0.1555	-0.0434	0.162	0.2043

rs13182737	A	G	A	G	0.1465	0.1576	0.2694	0.2928
rs13790142	A	T	A	T	0.5917	0.5104	0.0139	0.002702
rs13902590	T	C	T	C	0.2338	-0.343	0.0388	0.07047
rs14020686	T	C	T	C	0.2876	0.2478	0.0268	0.0106
rs14083741	A	G	A	G	0.3928	-0.8201	0.0169	0.03485
rs14205031	C	T	C	T	0.6265	0.6194	0.0159	0.01071
rs14235974	T	C	T	C	-0.3606	-0.0548	0.0229	0.01892
rs14387133	A	C	A	C	0.6513	-0.1154	0.0169	0.004854
rs14409171	T	C	T	C	0.1949	0.2105	0.0596	0.04122
rs14487293	T	C	T	C	0.4388	0.0803	0.0189	0.008147
rs14543580	A	G	A	G	0.8568	-0.2776	0.0109	0.05685
rs14550861	T	C	T	C	0.3384	0.1249	0.0199	0.03216
rs14662492	C	A	C	A	0.3365	-0.2274	0.0348	0.03037
rs14670133	A	G	A	G	1.2339	0.4771	0.0149	0.0383
rs14677509	G	A	G	A	0.5764	-0.2628	0.0109	0.02048
rs14712871	A	G	A	G	0.2786	-0.0128	0.0557	0.02502
rs14770389	C	G	C	G	-0.338	-0.3475	0.0249	0.0291
rs14834869	G	A	G	A	0.9175	1.7685	0.0109	0.001544
rs14882439	T	C	T	C	0.4987	-1.0178	0.0119	0.005948
rs14968727	A	G	A	G	0.4542	0.1427	0.0159	0.007371
rs15064946	C	G	C	G	-0.5142	-0.4598	0.0129	0.02747
rs1510889	C	T	C	T	0.1339	-0.213	0.672	0.7289
rs1543148	T	C	T	C	0.2643	-0.5468	0.0447	0.03059
rs1544218	G	A	G	A	-0.1349	0.0371	0.7724	0.7552
rs1670122	G	T	G	T	-0.8207	-0.1535	0.9712	0.92202
rs17125653	A	T	A	T	0.2355	-0.1117	0.0785	0.131
rs17206070	C	T	C	T	-0.6011	-0.2909	0.1968	0.1751
rs17392686	G	A	G	A	-0.5416	-1.0548	0.0169	0.002063
rs17396905	A	G	A	G	0.1933	0.0048	0.2177	0.2231
rs17600642	A	C	A	C	-0.1362	-0.3059	0.2048	0.1244
rs18479403	G	A	G	A	0.386	-0.3703	0.0169	0.01963
rs1869449	A	G	A	G	0.1769	0.0571	0.2962	0.2721
rs18962457	G	A	G	A	0.3936	-0.5806	0.0258	0.0177
rs19139817	A	G	A	G	1.0554	-0.3053	0.0119	0.02306
rs19232474	G	T	G	T	0.562	-0.2254	0.0139	0.01509
rs194749	C	T	C	T	-0.1638	-0.1214	0.2326	0.2782
rs202520	G	A	G	A	-0.1573	-0.1779	0.7147	0.6625
rs206763	A	G	A	G	0.6792	0.8363	0.0298	0.01553
rs2111485	G	A	G	A	0.1577	0.036	0.6024	0.5819
rs211510	G	A	G	A	0.1083	-0.0453	0.494	0.4919
rs2269247	T	C	T	C	0.1709	-0.1345	0.2058	0.3071
rs2297764	A	G	A	G	-0.2374	-0.3952	0.0547	0.05219
rs231971	G	A	G	A	0.2411	0.0222	0.0825	0.03878
rs2414899	C	T	C	T	0.191	-0.0096	0.0885	0.08746
rs2666237	A	G	A	G	0.1103	0.1097	0.4205	0.3753
rs2847293	T	A	T	A	-0.1569	0.0134	0.8608	0.8477
rs2872812	A	G	A	G	0.1274	0.1054	0.5964	0.6239

rs28799272	G	T	G	T	-0.1539	-0.0849	0.1252	0.1269
rs2949577	T	C	T	C	-0.1063	0.094	0.4453	0.4763
rs2963801	C	T	C	T	-0.1344	0.0813	0.6958	0.6773
rs3111414	G	C	G	C	-0.132	-0.2488	0.7903	0.7955
rs34296259	A	T	A	T	0.6637	0.1997	0.0268	0.05197
rs34536443	C	G	C	G	-0.4139	-0.1077	0.0288	0.03067
rs34664800	G	A	G	A	0.4329	0.1323	0.0308	0.02292
rs35013225	T	C	T	C	0.1247	-0.0411	0.3101	0.3396
rs362719	A	C	A	C	-0.1448	-0.2743	0.1839	0.212
rs372702	A	G	A	G	-0.112	-0.1267	0.5527	0.4881
rs39311	G	T	G	T	0.1271	-0.0806	0.3141	0.3221
rs4149965	A	G	A	G	-0.1416	0.2093	0.2505	0.2494
rs4514654	A	G	A	G	0.4303	0.5501	0.0268	0.01402
rs454854	C	G	C	G	0.1149	-0.0272	0.6869	0.676
rs4558785	T	A	T	A	0.1223	-0.1629	0.7197	0.7558
rs4566101	C	T	C	T	0.1755	-0.1444	0.2495	0.2809
rs4762523	A	G	A	G	-0.1293	-0.1375	0.7316	0.6941
rs5001561	T	C	T	C	-0.1718	-0.0792	0.8797	0.9332
rs506770	C	G	C	G	1.0048	-0.0341	0.8012	0.787
rs55861274	G	C	G	C	0.4135	-0.3848	0.0179	0.03185
rs55916920	G	T	G	T	-0.1984	-0.0408	0.1044	0.05896
rs55996894	C	G	C	G	-0.1785	-0.0734	0.164	0.1509
rs56391297	A	G	A	G	0.1656	0.0158	0.1352	0.1237
rs59680223	T	C	T	C	0.6421	-0.0714	0.0129	0.01619
rs60038159	A	T	A	T	0.192	0.1945	0.1064	0.07556
rs60888743	G	A	G	A	-0.1371	0.1473	0.2475	0.2311
rs61868788	G	A	G	A	-0.192	0.4169	0.0815	0.06485
rs61944737	G	A	G	A	0.1819	0.279	0.2127	0.1817
rs62123870	C	T	C	T	0.2176	-0.4137	0.0845	0.07587
rs62212655	T	C	T	C	0.2131	-0.3369	0.0855	0.04047
rs62267224	A	C	A	C	-0.3179	-0.1699	0.0398	0.04446
rs62410259	A	G	A	G	-0.3796	0.2879	0.0616	0.07955
rs6434441	A	G	A	G	-0.1091	0.1496	0.6471	0.6952
rs6503616	C	G	C	G	0.5501	2.6376	0.0109	0.001384
rs6544107	T	C	T	C	-0.1741	0.2166	0.1382	0.1366
rs66535411	G	A	G	A	0.4561	0.3652	0.0159	0.008654
rs6679677	A	C	A	C	0.6527	0.5606	0.0915	0.1468
rs6719660	G	A	G	A	0.2918	0.1054	0.9423	0.9602
rs6738028	C	G	C	G	-0.1081	0.013	0.5905	0.6053
rs689	T	A	T	A	0.7004	-0.0091	0.7256	0.791
rs71635549	T	C	T	C	0.3118	0.076	0.0477	0.02814
rs7246136	C	G	C	G	0.4003	0.7725	0.0229	0.01757
rs727251	A	G	A	G	0.1255	-0.1144	0.2545	0.3285
rs72828663	T	A	T	A	0.237	-0.0028	0.0676	0.1149
rs72842019	A	G	A	G	0.2228	-0.2225	0.0716	0.08137
rs72877865	T	A	T	A	-0.2638	0.1414	0.0567	0.04986
rs72940100	A	G	A	G	-0.1354	0.0188	0.2396	0.1623

rs72958430	A	C	A	C	0.2106	-0.0795	0.0825	0.1251
rs73136665	A	C	A	C	-0.4042	-0.0149	0.0199	0.01973
rs73261904	A	G	A	G	0.1897	0.129	0.1113	0.2017
rs73826337	A	G	A	G	0.1637	0.1376	0.1571	0.1338
rs741172	T	C	T	C	-0.2034	-0.2068	0.3221	0.2903
rs74388302	A	G	A	G	0.2584	0.3762	0.0775	0.05374
rs75107428	G	A	G	A	1.242	0.0529	0.0109	0.03706
rs75354435	A	G	A	G	0.5031	0.5308	0.0159	0.04742
rs75575119	A	C	A	C	0.6861	0.2725	0.0129	0.003479
rs76342469	A	G	A	G	0.6293	-0.2646	0.0199	0.01547
rs76495645	C	T	C	T	0.6948	0.0574	0.0109	0.01859
rs7670944	C	A	C	A	-0.8931	-0.3837	0.9851	0.966
rs7687596	G	A	G	A	0.1091	0.138	0.4592	0.5383
rs77064152	A	G	A	G	0.6225	0.0697	0.0129	0.03234
rs77089863	A	G	A	G	0.1435	-0.0466	0.1958	0.2534
rs77181159	A	G	A	G	-0.1589	-0.0083	0.1143	0.153
rs77308817	T	C	T	C	0.1345	0.0139	0.2386	0.22
rs7780389	C	T	C	T	0.2883	0.3419	0.9722	0.9752
rs78387855	T	C	T	C	0.3747	-0.0347	0.0368	0.08049
rs79315483	A	C	A	C	-0.2857	0.3521	0.0318	0.02708
rs79758851	C	G	C	G	0.34	-0.5053	0.0278	0.04924
rs80004547	A	G	A	G	0.351	-0.3407	0.0189	0.01811
rs80058612	A	G	A	G	-0.6066	-1.0818	0.0149	0.002488
rs80318013	T	A	T	A	-0.45	-0.5177	0.0119	0.01652
rs80348093	A	G	A	G	0.242	0.1205	0.0676	0.06242
rs8041124	G	A	G	A	0.1541	-0.0191	0.2684	0.3069
rs8056814	A	G	A	G	0.2641	-0.4022	0.0885	0.08281
rs8108092	A	G	A	G	0.1442	0.0197	0.3429	0.3003
rs8133961	A	G	A	G	0.5915	0.3403	0.0109	0.01573
rs860291	C	T	C	T	0.1642	0.0776	0.8946	0.9333
rs9273363	A	C	A	C	1.2786	-0.0905	0.2624	0.2799
rs9382199	C	T	C	T	-0.1357	-0.1834	0.2634	0.2387
rs9405098	A	G	A	G	-0.7798	-0.319	0.0239	0.02752
rs9501109	G	A	G	A	-0.243	-0.4314	0.2366	0.233
rs9522823	T	A	T	A	0.1212	-0.0683	0.3579	0.3275

remove	palindromic	ambiguous	id.outcome	chr	pos	se.outcome	samplesize.(	pval.outcom
FALSE	FALSE	FALSE	finn-b-I9_HY	4	156687170	0.1946		0.4917
FALSE	FALSE	FALSE	finn-b-I9_HY	8	12717076	0.3306		0.3395
FALSE	TRUE	FALSE	finn-b-I9_HY	11	16348430	0.2236		0.3832
FALSE	FALSE	FALSE	finn-b-I9_HY	2	74914779	0.1767		0.4971
FALSE	FALSE	FALSE	finn-b-I9_HY	3	131984573	0.4118		0.6418
FALSE	FALSE	FALSE	finn-b-I9_HY	14	51442670	0.1602		0.0306203
FALSE	FALSE	FALSE	finn-b-I9_HY	9	126981215	0.137		0.590101
FALSE	FALSE	FALSE	finn-b-I9_HY	12	111833788	0.1305		0.0139101
FALSE	FALSE	FALSE	finn-b-I9_HY	11	88890822	0.1264		0.4078
FALSE	FALSE	FALSE	finn-b-I9_HY	16	71969945	0.1276		0.9339
FALSE	TRUE	FALSE	finn-b-I9_HY	2	84040260	0.1371		0.673701
FALSE	FALSE	FALSE	finn-b-I9_HY	1	183646903	0.3865		0.729001
FALSE	FALSE	FALSE	finn-b-I9_HY	17	55751187	0.1393		0.6591
FALSE	TRUE	FALSE	finn-b-I9_HY	1	61490030	0.3646		0.764499
FALSE	FALSE	FALSE	finn-b-I9_HY	9	85817003	0.2353		0.6209
FALSE	FALSE	FALSE	finn-b-I9_HY	21	43836186	0.1418		0.752199
FALSE	FALSE	FALSE	finn-b-I9_HY	7	70641982	0.5642		0.2054
FALSE	TRUE	FALSE	finn-b-I9_HY	4	188786731	0.5149		0.1594
FALSE	FALSE	FALSE	finn-b-I9_HY	10	15231633	0.1423		0.457
FALSE	FALSE	FALSE	finn-b-I9_HY	5	168228401	0.4612		0.9729
FALSE	TRUE	FALSE	finn-b-I9_HY	12	41525726	0.5408		0.4294
FALSE	FALSE	FALSE	finn-b-I9_HY	1	38240331	0.8239		0.2127
FALSE	FALSE	FALSE	finn-b-I9_HY	1	228363750	0.4021		0.5604
FALSE	FALSE	FALSE	finn-b-I9_HY	4	37157098	0.2565		0.0556506
FALSE	FALSE	FALSE	finn-b-I9_HY	2	204745003	0.1311		0.9891
FALSE	FALSE	FALSE	finn-b-I9_HY	6	126801144	0.1275		0.6078
FALSE	FALSE	FALSE	finn-b-I9_HY	10	6471816	0.1381		0.4743
FALSE	TRUE	FALSE	finn-b-I9_HY	7	95642869	0.502		0.6612
FALSE	FALSE	FALSE	finn-b-I9_HY	3	55592454	0.135		0.3933
FALSE	FALSE	FALSE	finn-b-I9_HY	20	60166646	0.69		0.9351
FALSE	FALSE	FALSE	finn-b-I9_HY	7	148213731	1.4771		0.4662
FALSE	FALSE	FALSE	finn-b-I9_HY	4	123286227	0.1271		0.6528
FALSE	FALSE	FALSE	finn-b-I9_HY	8	69347954	0.8409		0.6732
FALSE	FALSE	FALSE	finn-b-I9_HY	11	9933451	0.4302		0.8213
FALSE	FALSE	FALSE	finn-b-I9_HY	2	62528070	0.2316		0.7381
FALSE	TRUE	FALSE	finn-b-I9_HY	4	94787162	0.141		0.9594
FALSE	FALSE	FALSE	finn-b-I9_HY	8	131870571	0.1389		0.2002
FALSE	FALSE	FALSE	finn-b-I9_HY	1	206978742	0.3644		0.3089
FALSE	FALSE	FALSE	finn-b-I9_HY	1	92205471	0.128		0.4801
FALSE	TRUE	FALSE	finn-b-I9_HY	9	33116941	0.1741		0.8441
FALSE	FALSE	FALSE	finn-b-I9_HY	8	111457175	0.1843		0.5679
FALSE	FALSE	FALSE	finn-b-I9_HY	5	63940262	0.1323		0.1196
FALSE	FALSE	FALSE	finn-b-I9_HY	2	98389086	0.1685		0.2867
FALSE	FALSE	FALSE	finn-b-I9_HY	10	6097283	0.2558		0.349
FALSE	FALSE	FALSE	finn-b-I9_HY	11	45943129	0.1956		0.4802
FALSE	FALSE	FALSE	finn-b-I9_HY	15	55496313	0.1577		0.7831

FALSE	FALSE	FALSE	finn-b-I9_HY	5	37642210	0.139	0.2569
FALSE	TRUE	FALSE	finn-b-I9_HY	9	127251457	1.3064	0.696
FALSE	FALSE	FALSE	finn-b-I9_HY	6	105462351	0.2524	0.1742
FALSE	FALSE	FALSE	finn-b-I9_HY	1	40712781	0.656	0.705601
FALSE	FALSE	FALSE	finn-b-I9_HY	8	139476488	0.3434	0.0169399
FALSE	FALSE	FALSE	finn-b-I9_HY	8	66334395	0.6177	0.316
FALSE	FALSE	FALSE	finn-b-I9_HY	16	1932695	0.4832	0.9098
FALSE	FALSE	FALSE	finn-b-I9_HY	16	84438965	0.9343	0.9017
FALSE	FALSE	FALSE	finn-b-I9_HY	2	118890378	0.3312	0.5251
FALSE	FALSE	FALSE	finn-b-I9_HY	5	125078575	0.7513	0.9149
FALSE	FALSE	FALSE	finn-b-I9_HY	8	84345182	0.2701	0.3039
FALSE	FALSE	FALSE	finn-b-I9_HY	18	65890173	0.3596	0.7284
FALSE	FALSE	FALSE	finn-b-I9_HY	14	41360460	0.3675	0.536
FALSE	FALSE	FALSE	finn-b-I9_HY	1	25986785	0.3294	0.1476
FALSE	FALSE	FALSE	finn-b-I9_HY	13	80268150	0.4394	0.5498
FALSE	FALSE	FALSE	finn-b-I9_HY	22	50194998	0.4202	0.9757
FALSE	TRUE	FALSE	finn-b-I9_HY	1	177729018	0.3754	0.3546
FALSE	FALSE	FALSE	finn-b-I9_HY	13	109139580	1.686	0.2942
FALSE	FALSE	FALSE	finn-b-I9_HY	19	58087868	0.8428	0.2272
FALSE	FALSE	FALSE	finn-b-I9_HY	8	143292929	0.7572	0.8505
FALSE	TRUE	FALSE	finn-b-I9_HY	1	92925654	0.3813	0.2278
FALSE	FALSE	FALSE	finn-b-I9_HY	12	78771331	0.1426	0.1354
FALSE	FALSE	FALSE	finn-b-I9_HY	3	2750328	0.3876	0.1583
FALSE	FALSE	FALSE	finn-b-I9_HY	1	151551078	0.1475	0.8014
FALSE	FALSE	FALSE	finn-b-I9_HY	6	79109432	0.2331	0.5101
FALSE	TRUE	FALSE	finn-b-I9_HY	14	89771348	0.1863	0.5488
FALSE	FALSE	FALSE	finn-b-I9_HY	6	32652361	0.1676	0.0827294
FALSE	FALSE	FALSE	finn-b-I9_HY	5	95757293	1.5442	0.4946
FALSE	FALSE	FALSE	finn-b-I9_HY	13	51242379	0.1523	0.9751
FALSE	FALSE	FALSE	finn-b-I9_HY	10	72462994	0.193	0.113
FALSE	FALSE	FALSE	finn-b-I9_HY	1	222249550	0.4724	0.4331
FALSE	FALSE	FALSE	finn-b-I9_HY	2	33613232	0.1431	0.6896
FALSE	FALSE	FALSE	finn-b-I9_HY	15	33774725	0.4788	0.2254
FALSE	FALSE	FALSE	finn-b-I9_HY	4	184913123	0.4277	0.4754
FALSE	FALSE	FALSE	finn-b-I9_HY	2	142300935	0.5136	0.6607
FALSE	FALSE	FALSE	finn-b-I9_HY	14	69273905	0.1409	0.3889
FALSE	FALSE	FALSE	finn-b-I9_HY	20	1675292	0.1334	0.1824
FALSE	FALSE	FALSE	finn-b-I9_HY	6	32970197	0.5167	0.1055
FALSE	FALSE	FALSE	finn-b-I9_HY	2	163110536	0.1287	0.7798
FALSE	FALSE	FALSE	finn-b-I9_HY	6	11593985	0.1265	0.7206
FALSE	FALSE	FALSE	finn-b-I9_HY	1	64107284	0.1371	0.3267
FALSE	FALSE	FALSE	finn-b-I9_HY	1	165622211	0.2905	0.1736
FALSE	FALSE	FALSE	finn-b-I9_HY	16	28536519	0.3311	0.9466
FALSE	FALSE	FALSE	finn-b-I9_HY	15	66564446	0.2231	0.9657
FALSE	FALSE	FALSE	finn-b-I9_HY	10	33418612	0.131	0.4023
FALSE	TRUE	FALSE	finn-b-I9_HY	18	12782448	0.1772	0.9396
FALSE	FALSE	FALSE	finn-b-I9_HY	17	38758650	0.1305	0.4194

FALSE	FALSE	FALSE	finn-b-I9_HY	3	131464109	0.1933	0.6603
FALSE	FALSE	FALSE	finn-b-I9_HY	15	31262808	0.1269	0.4589
FALSE	FALSE	FALSE	finn-b-I9_HY	5	3787719	0.1359	0.5495
FALSE	TRUE	FALSE	finn-b-I9_HY	2	8443859	0.1573	0.1137
FALSE	TRUE	FALSE	finn-b-I9_HY	9	12903066	0.2812	0.4776
FALSE	TRUE	FALSE	finn-b-I9_HY	19	10463118	0.3648	0.7678
FALSE	FALSE	FALSE	finn-b-I9_HY	15	39947202	0.4316	0.7591
FALSE	FALSE	FALSE	finn-b-I9_HY	13	22558477	0.1346	0.759899
FALSE	FALSE	FALSE	finn-b-I9_HY	7	103185877	0.1562	0.0790205
FALSE	FALSE	FALSE	finn-b-I9_HY	5	114862608	0.1267	0.3174
FALSE	FALSE	FALSE	finn-b-I9_HY	7	116954117	0.1362	0.5538
FALSE	FALSE	FALSE	finn-b-I9_HY	1	242035438	0.1459	0.1512
FALSE	FALSE	FALSE	finn-b-I9_HY	15	91341067	0.5549	0.3215
FALSE	TRUE	FALSE	finn-b-I9_HY	18	77253866	0.1358	0.8414
FALSE	TRUE	FALSE	finn-b-I9_HY	3	182836957	0.1478	0.2703
FALSE	FALSE	FALSE	finn-b-I9_HY	15	49219631	0.1409	0.3053
FALSE	FALSE	FALSE	finn-b-I9_HY	12	99294499	0.1375	0.3173
FALSE	FALSE	FALSE	finn-b-I9_HY	11	20101425	0.2616	0.762
FALSE	TRUE	FALSE	finn-b-I9_HY	6	31785228	0.1593	0.8304
FALSE	TRUE	FALSE	finn-b-I9_HY	9	89182001	0.3591	0.2839
FALSE	FALSE	FALSE	finn-b-I9_HY	6	170376383	0.2693	0.8796
FALSE	TRUE	FALSE	finn-b-I9_HY	14	101302463	0.1789	0.681601
FALSE	FALSE	FALSE	finn-b-I9_HY	9	139521363	0.1942	0.9352
FALSE	FALSE	FALSE	finn-b-I9_HY	12	74547684	0.4891	0.8839
FALSE	TRUE	FALSE	finn-b-I9_HY	9	135851375	0.2435	0.4245
FALSE	FALSE	FALSE	finn-b-I9_HY	10	90051317	0.15	0.326
FALSE	FALSE	FALSE	finn-b-I9_HY	10	78771091	0.2631	0.1131
FALSE	FALSE	FALSE	finn-b-I9_HY	12	118935005	0.1651	0.0911192
FALSE	FALSE	FALSE	finn-b-I9_HY	19	18618565	0.2393	0.0838494
FALSE	FALSE	FALSE	finn-b-I9_HY	20	4197678	0.3352	0.3148
FALSE	FALSE	FALSE	finn-b-I9_HY	3	97965107	0.3129	0.5872
FALSE	FALSE	FALSE	finn-b-I9_HY	4	41713671	0.2351	0.2208
FALSE	FALSE	FALSE	finn-b-I9_HY	2	192000082	0.1382	0.2791
FALSE	TRUE	FALSE	finn-b-I9_HY	17	36591190	1.9451	0.1751
FALSE	FALSE	FALSE	finn-b-I9_HY	2	38009648	0.186	0.244
FALSE	FALSE	FALSE	finn-b-I9_HY	2	89098162	0.6981	0.6009
FALSE	FALSE	FALSE	finn-b-I9_HY	1	114303808	0.1797	0.001809
FALSE	FALSE	FALSE	finn-b-I9_HY	2	130096389	0.3249	0.7456
FALSE	TRUE	FALSE	finn-b-I9_HY	2	111949327	0.1298	0.9203
FALSE	TRUE	FALSE	finn-b-I9_HY	11	2182224	0.1579	0.9543
FALSE	FALSE	FALSE	finn-b-I9_HY	1	201528637	0.395	0.8474
FALSE	TRUE	FALSE	finn-b-I9_HY	19	55016364	0.5023	0.1241
FALSE	FALSE	FALSE	finn-b-I9_HY	4	82333127	0.1368	0.4029
FALSE	TRUE	FALSE	finn-b-I9_HY	5	169529091	0.1993	0.9889
FALSE	FALSE	FALSE	finn-b-I9_HY	10	102845714	0.2337	0.3411
FALSE	TRUE	FALSE	finn-b-I9_HY	6	65754261	0.3024	0.6402
FALSE	FALSE	FALSE	finn-b-I9_HY	11	81999343	0.1736	0.9136

FALSE	FALSE	FALSE	finn-b-I9_HY	6	122034125	0.1893	0.674401
FALSE	FALSE	FALSE	finn-b-I9_HY	3	99600236	0.4714	0.9748
FALSE	FALSE	FALSE	finn-b-I9_HY	17	13648344	0.1597	0.4193
FALSE	FALSE	FALSE	finn-b-I9_HY	3	33049177	0.186	0.459501
FALSE	FALSE	FALSE	finn-b-I9_HY	16	11200798	0.1388	0.1362
FALSE	FALSE	FALSE	finn-b-I9_HY	7	149781689	0.2957	0.2033
FALSE	FALSE	FALSE	finn-b-I9_HY	2	132137277	0.3347	0.8745
FALSE	FALSE	FALSE	finn-b-I9_HY	7	144108878	0.2974	0.0743002
FALSE	FALSE	FALSE	finn-b-I9_HY	3	56691795	1.1385	0.8108
FALSE	FALSE	FALSE	finn-b-I9_HY	6	29345680	0.5022	0.598199
FALSE	FALSE	FALSE	finn-b-I9_HY	7	83492582	0.4648	0.9017
FALSE	FALSE	FALSE	finn-b-I9_HY	4	188272285	0.3446	0.2655
FALSE	FALSE	FALSE	finn-b-I9_HY	4	5472092	0.1276	0.2794
FALSE	FALSE	FALSE	finn-b-I9_HY	3	96887901	0.352	0.843
FALSE	FALSE	FALSE	finn-b-I9_HY	4	27078208	0.1459	0.749501
FALSE	FALSE	FALSE	finn-b-I9_HY	7	28172124	0.1751	0.9623
FALSE	FALSE	FALSE	finn-b-I9_HY	2	174883122	0.1546	0.9284
FALSE	FALSE	FALSE	finn-b-I9_HY	7	51015193	0.4151	0.4101
FALSE	FALSE	FALSE	finn-b-I9_HY	7	41014604	0.2334	0.8817
FALSE	FALSE	FALSE	finn-b-I9_HY	5	141111541	0.3949	0.3726
FALSE	TRUE	FALSE	finn-b-I9_HY	18	27189013	0.2943	0.0860597
FALSE	FALSE	FALSE	finn-b-I9_HY	7	53255329	0.4758	0.474
FALSE	FALSE	FALSE	finn-b-I9_HY	19	47189331	1.3226	0.4134
FALSE	TRUE	FALSE	finn-b-I9_HY	4	48381637	0.5052	0.3055
FALSE	FALSE	FALSE	finn-b-I9_HY	3	149080396	0.2685	0.653699
FALSE	FALSE	FALSE	finn-b-I9_HY	15	50434908	0.1378	0.8895
FALSE	FALSE	FALSE	finn-b-I9_HY	16	75252327	0.231	0.0816902
FALSE	FALSE	FALSE	finn-b-I9_HY	19	3138091	0.139	0.8875
FALSE	FALSE	FALSE	finn-b-I9_HY	21	45133493	0.5278	0.5191
FALSE	FALSE	FALSE	finn-b-I9_HY	3	186557957	0.2577	0.763199
FALSE	FALSE	FALSE	finn-b-I9_HY	6	32626272	0.1511	0.5495
FALSE	FALSE	FALSE	finn-b-I9_HY	6	53209120	0.1505	0.2229
FALSE	FALSE	FALSE	finn-b-I9_HY	6	32379736	0.378	0.3987
FALSE	FALSE	FALSE	finn-b-I9_HY	6	31392118	0.1813	0.0173301
FALSE	TRUE	FALSE	finn-b-I9_HY	13	90729353	0.1352	0.6136

outcome	originalnam	outcome.de	mr_keep.ou	data_source	proxy.outco	target_snp.c	proxy_snp.o	target_a1.ou
Hypertensio	Hypertensio	Hypertensio	TRUE	igd				
Hypertensio	Hypertensio	Hypertensio	TRUE	igd				
Hypertensio	Hypertensio	Hypertensio	TRUE	igd				
Hypertensio	Hypertensio	Hypertensio	TRUE	igd				
Hypertensio	Hypertensio	Hypertensio	TRUE	igd				
Hypertensio	Hypertensio	Hypertensio	TRUE	igd				
Hypertensio	Hypertensio	Hypertensio	TRUE	igd				
Hypertensio	Hypertensio	Hypertensio	TRUE	igd				
Hypertensio	Hypertensio	Hypertensio	TRUE	igd				
Hypertensio	Hypertensio	Hypertensio	TRUE	igd				
Hypertensio	Hypertensio	Hypertensio	TRUE	igd				
Hypertensio	Hypertensio	Hypertensio	TRUE	igd				
Hypertensio	Hypertensio	Hypertensio	TRUE	igd				
Hypertensio	Hypertensio	Hypertensio	TRUE	igd				
Hypertensio	Hypertensio	Hypertensio	TRUE	igd				
Hypertensio	Hypertensio	Hypertensio	TRUE	igd				
Hypertensio	Hypertensio	Hypertensio	TRUE	igd				
Hypertensio	Hypertensio	Hypertensio	TRUE	igd				
Hypertensio	Hypertensio	Hypertensio	TRUE	igd				
Hypertensio	Hypertensio	Hypertensio	TRUE	igd				
Hypertensio	Hypertensio	Hypertensio	TRUE	igd				
Hypertensio	Hypertensio	Hypertensio	TRUE	igd				
Hypertensio	Hypertensio	Hypertensio	TRUE	igd				
Hypertensio	Hypertensio	Hypertensio	TRUE	igd				
Hypertensio	Hypertensio	Hypertensio	TRUE	igd				
Hypertensio	Hypertensio	Hypertensio	TRUE	igd				
Hypertensio	Hypertensio	Hypertensio	TRUE	igd				
Hypertensio	Hypertensio	Hypertensio	TRUE	igd				
Hypertensio	Hypertensio	Hypertensio	TRUE	igd	TRUE	rs11717237	rs56115292	T
Hypertensio	Hypertensio	Hypertensio	TRUE	igd				
Hypertensio	Hypertensio	Hypertensio	TRUE	igd				
Hypertensio	Hypertensio	Hypertensio	TRUE	igd				
Hypertensio	Hypertensio	Hypertensio	TRUE	igd				
Hypertensio	Hypertensio	Hypertensio	TRUE	igd				
Hypertensio	Hypertensio	Hypertensio	TRUE	igd				
Hypertensio	Hypertensio	Hypertensio	TRUE	igd				
Hypertensio	Hypertensio	Hypertensio	TRUE	igd				
Hypertensio	Hypertensio	Hypertensio	TRUE	igd				
Hypertensio	Hypertensio	Hypertensio	TRUE	igd				
Hypertensio	Hypertensio	Hypertensio	TRUE	igd				
Hypertensio	Hypertensio	Hypertensio	TRUE	igd				
Hypertensio	Hypertensio	Hypertensio	TRUE	igd				
Hypertensio	Hypertensio	Hypertensio	TRUE	igd				
Hypertensio	Hypertensio	Hypertensio	TRUE	igd				
Hypertensio	Hypertensio	Hypertensio	TRUE	igd				
Hypertensio	Hypertensio	Hypertensio	TRUE	igd				

Hypertensio	Hypertensio	Hypertensio	TRUE	igd				
Hypertensio	Hypertensio	Hypertensio	TRUE	igd				
Hypertensio	Hypertensio	Hypertensio	TRUE	igd				
Hypertensio	Hypertensio	Hypertensio	TRUE	igd				
Hypertensio	Hypertensio	Hypertensio	TRUE	igd				
Hypertensio	Hypertensio	Hypertensio	TRUE	igd				
Hypertensio	Hypertensio	Hypertensio	TRUE	igd				
Hypertensio	Hypertensio	Hypertensio	TRUE	igd				
Hypertensio	Hypertensio	Hypertensio	TRUE	igd				
Hypertensio	Hypertensio	Hypertensio	TRUE	igd				
Hypertensio	Hypertensio	Hypertensio	TRUE	igd				
Hypertensio	Hypertensio	Hypertensio	TRUE	igd				
Hypertensio	Hypertensio	Hypertensio	TRUE	igd				
Hypertensio	Hypertensio	Hypertensio	TRUE	igd				
Hypertensio	Hypertensio	Hypertensio	TRUE	igd				
Hypertensio	Hypertensio	Hypertensio	TRUE	igd				
Hypertensio	Hypertensio	Hypertensio	TRUE	igd				
Hypertensio	Hypertensio	Hypertensio	TRUE	igd				
Hypertensio	Hypertensio	Hypertensio	TRUE	igd				
Hypertensio	Hypertensio	Hypertensio	TRUE	igd				
Hypertensio	Hypertensio	Hypertensio	TRUE	igd				
Hypertensio	Hypertensio	Hypertensio	TRUE	igd				
Hypertensio	Hypertensio	Hypertensio	TRUE	igd				
Hypertensio	Hypertensio	Hypertensio	TRUE	igd				
Hypertensio	Hypertensio	Hypertensio	TRUE	igd	TRUE	rs1670122	rs73762626	T
Hypertensio	Hypertensio	Hypertensio	TRUE	igd				
Hypertensio	Hypertensio	Hypertensio	TRUE	igd				
Hypertensio	Hypertensio	Hypertensio	TRUE	igd				
Hypertensio	Hypertensio	Hypertensio	TRUE	igd				
Hypertensio	Hypertensio	Hypertensio	TRUE	igd				
Hypertensio	Hypertensio	Hypertensio	TRUE	igd				
Hypertensio	Hypertensio	Hypertensio	TRUE	igd				
Hypertensio	Hypertensio	Hypertensio	TRUE	igd				
Hypertensio	Hypertensio	Hypertensio	TRUE	igd				
Hypertensio	Hypertensio	Hypertensio	TRUE	igd				
Hypertensio	Hypertensio	Hypertensio	TRUE	igd				
Hypertensio	Hypertensio	Hypertensio	TRUE	igd				
Hypertensio	Hypertensio	Hypertensio	TRUE	igd				
Hypertensio	Hypertensio	Hypertensio	TRUE	igd				
Hypertensio	Hypertensio	Hypertensio	TRUE	igd				
Hypertensio	Hypertensio	Hypertensio	TRUE	igd				
Hypertensio	Hypertensio	Hypertensio	TRUE	igd				
Hypertensio	Hypertensio	Hypertensio	TRUE	igd				
Hypertensio	Hypertensio	Hypertensio	TRUE	igd				
Hypertensio	Hypertensio	Hypertensio	TRUE	igd				
Hypertensio	Hypertensio	Hypertensio	TRUE	igd				
Hypertensio	Hypertensio	Hypertensio	TRUE	igd				

Hypertensio	Hypertensio	Hypertensio	TRUE	igd					
Hypertensio	Hypertensio	Hypertensio	TRUE	igd					
Hypertensio	Hypertensio	Hypertensio	TRUE	igd					
Hypertensio	Hypertensio	Hypertensio	TRUE	igd					
Hypertensio	Hypertensio	Hypertensio	TRUE	igd					
Hypertensio	Hypertensio	Hypertensio	TRUE	igd					
Hypertensio	Hypertensio	Hypertensio	TRUE	igd					
Hypertensio	Hypertensio	Hypertensio	TRUE	igd					
Hypertensio	Hypertensio	Hypertensio	TRUE	igd					
Hypertensio	Hypertensio	Hypertensio	TRUE	igd					
Hypertensio	Hypertensio	Hypertensio	TRUE	igd					
Hypertensio	Hypertensio	Hypertensio	TRUE	igd					
Hypertensio	Hypertensio	Hypertensio	TRUE	igd					
Hypertensio	Hypertensio	Hypertensio	TRUE	igd					
Hypertensio	Hypertensio	Hypertensio	TRUE	igd					
Hypertensio	Hypertensio	Hypertensio	TRUE	igd					
Hypertensio	Hypertensio	Hypertensio	TRUE	igd					
Hypertensio	Hypertensio	Hypertensio	TRUE	igd					
Hypertensio	Hypertensio	Hypertensio	TRUE	igd					
Hypertensio	Hypertensio	Hypertensio	TRUE	igd					
Hypertensio	Hypertensio	Hypertensio	TRUE	igd					
Hypertensio	Hypertensio	Hypertensio	TRUE	igd					
Hypertensio	Hypertensio	Hypertensio	TRUE	igd					
Hypertensio	Hypertensio	Hypertensio	TRUE	igd					
Hypertensio	Hypertensio	Hypertensio	TRUE	igd					
Hypertensio	Hypertensio	Hypertensio	TRUE	igd					
Hypertensio	Hypertensio	Hypertensio	TRUE	igd					
Hypertensio	Hypertensio	Hypertensio	TRUE	igd					
Hypertensio	Hypertensio	Hypertensio	TRUE	igd					
Hypertensio	Hypertensio	Hypertensio	TRUE	igd					
Hypertensio	Hypertensio	Hypertensio	TRUE	igd		TRUE	rs62267224	rs11345811	A
Hypertensio	Hypertensio	Hypertensio	TRUE	igd					
Hypertensio	Hypertensio	Hypertensio	TRUE	igd					
Hypertensio	Hypertensio	Hypertensio	TRUE	igd					
Hypertensio	Hypertensio	Hypertensio	TRUE	igd					
Hypertensio	Hypertensio	Hypertensio	TRUE	igd					
Hypertensio	Hypertensio	Hypertensio	TRUE	igd					
Hypertensio	Hypertensio	Hypertensio	TRUE	igd					
Hypertensio	Hypertensio	Hypertensio	TRUE	igd					
Hypertensio	Hypertensio	Hypertensio	TRUE	igd					
Hypertensio	Hypertensio	Hypertensio	TRUE	igd					
Hypertensio	Hypertensio	Hypertensio	TRUE	igd					
Hypertensio	Hypertensio	Hypertensio	TRUE	igd					
Hypertensio	Hypertensio	Hypertensio	TRUE	igd					
Hypertensio	Hypertensio	Hypertensio	TRUE	igd					
Hypertensio	Hypertensio	Hypertensio	TRUE	igd					
Hypertensio	Hypertensio	Hypertensio	TRUE	igd					

Hypertensio	Hypertensio	Hypertensio	TRUE	igd					
Hypertensio	Hypertensio	Hypertensio	TRUE	igd					
Hypertensio	Hypertensio	Hypertensio	TRUE	igd					
Hypertensio	Hypertensio	Hypertensio	TRUE	igd					
Hypertensio	Hypertensio	Hypertensio	TRUE	igd					
Hypertensio	Hypertensio	Hypertensio	TRUE	igd					
Hypertensio	Hypertensio	Hypertensio	TRUE	igd		TRUE	rs75107428	rs11794076	G
Hypertensio	Hypertensio	Hypertensio	TRUE	igd					
Hypertensio	Hypertensio	Hypertensio	TRUE	igd					
Hypertensio	Hypertensio	Hypertensio	TRUE	igd					
Hypertensio	Hypertensio	Hypertensio	TRUE	igd					
Hypertensio	Hypertensio	Hypertensio	TRUE	igd					
Hypertensio	Hypertensio	Hypertensio	TRUE	igd					
Hypertensio	Hypertensio	Hypertensio	TRUE	igd					
Hypertensio	Hypertensio	Hypertensio	TRUE	igd					
Hypertensio	Hypertensio	Hypertensio	TRUE	igd					
Hypertensio	Hypertensio	Hypertensio	TRUE	igd					
Hypertensio	Hypertensio	Hypertensio	TRUE	igd					
Hypertensio	Hypertensio	Hypertensio	TRUE	igd					
Hypertensio	Hypertensio	Hypertensio	TRUE	igd					
Hypertensio	Hypertensio	Hypertensio	TRUE	igd					
Hypertensio	Hypertensio	Hypertensio	TRUE	igd					
Hypertensio	Hypertensio	Hypertensio	TRUE	igd					
Hypertensio	Hypertensio	Hypertensio	TRUE	igd					
Hypertensio	Hypertensio	Hypertensio	TRUE	igd					
Hypertensio	Hypertensio	Hypertensio	TRUE	igd					
Hypertensio	Hypertensio	Hypertensio	TRUE	igd					
Hypertensio	Hypertensio	Hypertensio	TRUE	igd					
Hypertensio	Hypertensio	Hypertensio	TRUE	igd					
Hypertensio	Hypertensio	Hypertensio	TRUE	igd					
Hypertensio	Hypertensio	Hypertensio	TRUE	igd					
Hypertensio	Hypertensio	Hypertensio	TRUE	igd					
Hypertensio	Hypertensio	Hypertensio	TRUE	igd					
Hypertensio	Hypertensio	Hypertensio	TRUE	igd					
Hypertensio	Hypertensio	Hypertensio	TRUE	igd		TRUE	rs9522823	rs9522841	T

target_a2.ou	proxy_a1.ou	proxy_a2.ou	chr.exposure	pos.exposur	se.exposure	pval.exposu	samplesize.e	id.exposure
			4	156687170	0.0382	9.147E-07	24840	ebi-a-GCST0
			8	12717076	0.0983	3.897E-06	24840	ebi-a-GCST0
			11	16348430	0.037	9.499E-06	24840	ebi-a-GCST0
			2	74914779	0.0322	1.816E-10	24840	ebi-a-GCST0
			3	131984573	0.1588	3.52E-10	24840	ebi-a-GCST0
			14	51442670	0.0311	3.692E-07	24840	ebi-a-GCST0
			9	126981215	0.0243	2.427E-08	24840	ebi-a-GCST0
			12	111833788	0.0244	1.343E-25	24840	ebi-a-GCST0
			11	88890822	0.0233	1.024E-11	24840	ebi-a-GCST0
			16	71969945	0.0239	5.804E-07	24840	ebi-a-GCST0
			2	84040260	0.0277	4.664E-09	24840	ebi-a-GCST0
			1	183646903	0.064	6.747E-09	24840	ebi-a-GCST0
			17	55751187	0.0268	2.092E-06	24840	ebi-a-GCST0
			1	61490030	0.1214	2.735E-06	24840	ebi-a-GCST0
			9	85817003	0.0932	2.744E-06	24840	ebi-a-GCST0
			21	43836186	0.0242	1.558E-06	24840	ebi-a-GCST0
			7	70641982	0.0931	1.674E-07	24840	ebi-a-GCST0
			4	188786731	0.0805	3.138E-06	24840	ebi-a-GCST0
			10	15231633	0.0291	2.339E-06	24840	ebi-a-GCST0
			5	168228401	0.0812	4.445E-06	24840	ebi-a-GCST0
			12	41525726	0.0951	3.592E-06	24840	ebi-a-GCST0
			1	38240331	0.093	8.984E-06	24840	ebi-a-GCST0
			1	228363750	0.0528	1.659E-06	24840	ebi-a-GCST0
			4	37157098	0.0735	7.498E-06	24840	ebi-a-GCST0
			2	204745003	0.0237	1.114E-16	24840	ebi-a-GCST0
			6	126801144	0.0232	2.856E-06	24840	ebi-a-GCST0
			10	6471816	0.0256	2.458E-06	24840	ebi-a-GCST0
			7	95642869	0.0698	4.449E-07	24840	ebi-a-GCST0
C	T	C	3	55587016	0.0246	1.801E-06	24840	ebi-a-GCST0
			20	60166646	0.1764	1.156E-06	24840	ebi-a-GCST0
			7	148213731	0.1238	6.865E-06	24840	ebi-a-GCST0
			4	123286227	0.0242	1.88E-06	24840	ebi-a-GCST0
			8	69347954	0.0879	7.949E-07	24840	ebi-a-GCST0
			11	9933451	0.0794	7.673E-07	24840	ebi-a-GCST0
			2	62528070	0.0402	3.636E-07	24840	ebi-a-GCST0
			4	94787162	0.029	2.123E-06	24840	ebi-a-GCST0
			8	131870571	0.0239	3.746E-07	24840	ebi-a-GCST0
			1	206978742	0.065	3.772E-06	24840	ebi-a-GCST0
			1	92205471	0.0244	9.296E-06	24840	ebi-a-GCST0
			9	33116941	0.031	2.542E-06	24840	ebi-a-GCST0
			8	111457175	0.0335	5.951E-08	24840	ebi-a-GCST0
			5	63940262	0.0243	1.586E-06	24840	ebi-a-GCST0
			2	98389086	0.0243	1.584E-06	24840	ebi-a-GCST0
			10	6097283	0.0408	1.269E-14	24840	ebi-a-GCST0
			11	45943129	0.042	6.575E-07	24840	ebi-a-GCST0
			15	55496313	0.035	8.818E-06	24840	ebi-a-GCST0

			5	37642210	0.0259	1.486E-08	24840	ebi-a-GCST0
			9	127251457	0.1189	6.462E-07	24840	ebi-a-GCST0
			6	105462351	0.0507	3.982E-06	24840	ebi-a-GCST0
			1	40712781	0.0632	5.291E-06	24840	ebi-a-GCST0
			8	139476488	0.0878	7.729E-06	24840	ebi-a-GCST0
			8	66334395	0.1328	2.394E-06	24840	ebi-a-GCST0
			16	1932695	0.08	6.612E-06	24840	ebi-a-GCST0
			16	84438965	0.1353	1.471E-06	24840	ebi-a-GCST0
			2	118890378	0.0439	8.965E-06	24840	ebi-a-GCST0
			5	125078575	0.0822	9.344E-08	24840	ebi-a-GCST0
			8	84345182	0.1933	9.261E-06	24840	ebi-a-GCST0
			18	65890173	0.075	6.352E-06	24840	ebi-a-GCST0
			14	41360460	0.0749	6.961E-06	24840	ebi-a-GCST0
			1	25986785	0.2425	3.627E-07	24840	ebi-a-GCST0
			13	80268150	0.1176	9.527E-07	24840	ebi-a-GCST0
			22	50194998	0.0615	5.832E-06	24840	ebi-a-GCST0
			1	177729018	0.0705	1.628E-06	24840	ebi-a-GCST0
			13	109139580	0.2059	8.39E-06	24840	ebi-a-GCST0
			19	58087868	0.1124	9.161E-06	24840	ebi-a-GCST0
			8	143292929	0.0984	3.89E-06	24840	ebi-a-GCST0
			1	92925654	0.1119	4.343E-06	24840	ebi-a-GCST0
			12	78771331	0.0259	2.31E-07	24840	ebi-a-GCST0
			3	2750328	0.0552	1.677E-06	24840	ebi-a-GCST0
			1	151551078	0.0293	4.137E-06	24840	ebi-a-GCST0
G	C	T	6	79035739	0.1626	4.493E-07	24840	ebi-a-GCST0
			14	89771348	0.0402	4.754E-09	24840	ebi-a-GCST0
			6	32652361	0.0513	9.337E-32	24840	ebi-a-GCST0
			5	95757293	0.109	6.775E-07	24840	ebi-a-GCST0
			13	51242379	0.0357	6.013E-08	24840	ebi-a-GCST0
			10	72462994	0.0305	8.244E-06	24840	ebi-a-GCST0
			1	222249550	0.078	7.512E-07	24840	ebi-a-GCST0
			2	33613232	0.0269	4.552E-11	24840	ebi-a-GCST0
			15	33774725	0.0863	5.065E-06	24840	ebi-a-GCST0
			4	184913123	0.2335	6.196E-06	24840	ebi-a-GCST0
			2	142300935	0.0875	1.365E-10	24840	ebi-a-GCST0
			14	69273905	0.0281	5.369E-09	24840	ebi-a-GCST0
			20	1675292	0.0256	7.971E-10	24840	ebi-a-GCST0
			6	32970197	0.0779	2.927E-18	24840	ebi-a-GCST0
			2	163110536	0.0248	1.892E-10	24840	ebi-a-GCST0
			6	11593985	0.0236	4.485E-06	24840	ebi-a-GCST0
			1	64107284	0.0295	7.279E-09	24840	ebi-a-GCST0
			1	165622211	0.0497	1.747E-06	24840	ebi-a-GCST0
			16	28536519	0.0399	1.552E-09	24840	ebi-a-GCST0
			15	66564446	0.0396	1.442E-06	24840	ebi-a-GCST0
			10	33418612	0.0237	3.199E-06	24840	ebi-a-GCST0
			18	12782448	0.0307	3.341E-07	24840	ebi-a-GCST0
			17	38758650	0.0247	2.532E-07	24840	ebi-a-GCST0

			3	131464109	0.0338	5.425E-06	24840	ebi-a-GCST0
			15	31262808	0.023	3.701E-06	24840	ebi-a-GCST0
			5	3787719	0.0272	7.863E-07	24840	ebi-a-GCST0
			2	8443859	0.0289	4.798E-06	24840	ebi-a-GCST0
			9	12903066	0.1171	1.427E-08	24840	ebi-a-GCST0
			19	10463118	0.0665	4.843E-10	24840	ebi-a-GCST0
			15	39947202	0.0951	5.352E-06	24840	ebi-a-GCST0
			13	22558477	0.0281	8.723E-06	24840	ebi-a-GCST0
			7	103185877	0.0319	5.701E-06	24840	ebi-a-GCST0
			5	114862608	0.0228	9.213E-07	24840	ebi-a-GCST0
			7	116954117	0.0258	8.473E-07	24840	ebi-a-GCST0
			1	242035438	0.0294	1.452E-06	24840	ebi-a-GCST0
			15	91341067	0.0875	8.755E-07	24840	ebi-a-GCST0
			18	77253866	0.0258	8.599E-06	24840	ebi-a-GCST0
			3	182836957	0.0269	5.493E-06	24840	ebi-a-GCST0
			15	49219631	0.0255	6.229E-12	24840	ebi-a-GCST0
			12	99294499	0.0244	1.198E-07	24840	ebi-a-GCST0
			11	20101425	0.0334	2.671E-07	24840	ebi-a-GCST0
			6	31785228	0.0426	3.33E-123	24840	ebi-a-GCST0
			9	89182001	0.0768	7.234E-08	24840	ebi-a-GCST0
			6	170376383	0.0395	5.277E-07	24840	ebi-a-GCST0
			14	101302463	0.0323	3.128E-08	24840	ebi-a-GCST0
			9	139521363	0.037	7.803E-06	24840	ebi-a-GCST0
			12	74547684	0.1032	4.998E-10	24840	ebi-a-GCST0
			9	135851375	0.0384	5.585E-07	24840	ebi-a-GCST0
			10	90051317	0.0274	5.465E-07	24840	ebi-a-GCST0
			10	78771091	0.0405	2.18E-06	24840	ebi-a-GCST0
			12	118935005	0.0297	8.847E-10	24840	ebi-a-GCST0
			19	18618565	0.0449	1.282E-06	24840	ebi-a-GCST0
			20	4197678	0.0395	7.025E-08	24840	ebi-a-GCST0
C	A	G	3	97962864	0.0665	1.744E-06	24840	ebi-a-GCST0
			4	41713671	0.0533	1.018E-12	24840	ebi-a-GCST0
			2	192000082	0.0243	6.802E-06	24840	ebi-a-GCST0
			17	36591190	0.118	3.155E-06	24840	ebi-a-GCST0
			2	38009648	0.0346	4.761E-07	24840	ebi-a-GCST0
			2	89098162	0.0877	1.957E-07	24840	ebi-a-GCST0
			1	114303808	0.0346	3.418E-79	24840	ebi-a-GCST0
			2	130096389	0.0524	2.517E-08	24840	ebi-a-GCST0
			2	111949327	0.0244	9.477E-06	24840	ebi-a-GCST0
			11	2182224	0.0354	2.303E-87	24840	ebi-a-GCST0
			1	201528637	0.0682	4.839E-06	24840	ebi-a-GCST0
			19	55016364	0.0757	1.26E-07	24840	ebi-a-GCST0
			4	82333127	0.0284	9.539E-06	24840	ebi-a-GCST0
			5	169529091	0.047	4.643E-07	24840	ebi-a-GCST0
			10	102845714	0.0483	4.045E-06	24840	ebi-a-GCST0
			6	65754261	0.0513	2.709E-07	24840	ebi-a-GCST0
			11	81999343	0.0276	9.641E-07	24840	ebi-a-GCST0

			6	122034125	0.0411	2.931E-07	24840	ebi-a-GCST0
			3	99600236	0.0897	6.621E-06	24840	ebi-a-GCST0
			17	13648344	0.0412	4.09E-06	24840	ebi-a-GCST0
			3	33049177	0.0346	2.238E-06	24840	ebi-a-GCST0
			16	11200798	0.0258	3.114E-15	24840	ebi-a-GCST0
			7	149781689	0.0566	4.953E-06	24840	ebi-a-GCST0
A	A	G	2	132125129	0.2729	5.321E-06	24840	ebi-a-GCST0
			7	144108878	0.1104	5.137E-06	24840	ebi-a-GCST0
			3	56691795	0.1367	5.218E-07	24840	ebi-a-GCST0
			6	29345680	0.0898	2.472E-12	24840	ebi-a-GCST0
			7	83492582	0.1483	2.807E-06	24840	ebi-a-GCST0
			4	188272285	0.1885	2.148E-06	24840	ebi-a-GCST0
			4	5472092	0.0238	4.537E-06	24840	ebi-a-GCST0
			3	96887901	0.1275	1.052E-06	24840	ebi-a-GCST0
			4	27078208	0.0322	8.393E-06	24840	ebi-a-GCST0
			7	28172124	0.0348	4.986E-06	24840	ebi-a-GCST0
			2	174883122	0.0285	2.445E-06	24840	ebi-a-GCST0
			7	51015193	0.0594	1.195E-06	24840	ebi-a-GCST0
			7	41014604	0.0723	2.187E-07	24840	ebi-a-GCST0
			5	141111541	0.0577	7.437E-07	24840	ebi-a-GCST0
			18	27189013	0.0769	9.719E-06	24840	ebi-a-GCST0
			7	53255329	0.0787	8.218E-06	24840	ebi-a-GCST0
			19	47189331	0.1189	3.352E-07	24840	ebi-a-GCST0
			4	48381637	0.1017	9.751E-06	24840	ebi-a-GCST0
			3	149080396	0.0534	5.73E-06	24840	ebi-a-GCST0
			15	50434908	0.0293	1.487E-07	24840	ebi-a-GCST0
			16	75252327	0.0415	1.987E-10	24840	ebi-a-GCST0
			19	3138091	0.0308	2.837E-06	24840	ebi-a-GCST0
			21	45133493	0.1151	2.785E-07	24840	ebi-a-GCST0
			3	186557957	0.0354	3.457E-06	24840	ebi-a-GCST0
			6	32626272	0.0334	1E-200	24840	ebi-a-GCST0
			6	53209120	0.0255	1.086E-07	24840	ebi-a-GCST0
			6	32379736	0.1269	8.052E-10	24840	ebi-a-GCST0
			6	31392118	0.0354	6.568E-12	24840	ebi-a-GCST0
A	T	C	13	90682641	0.0239	0.0000004	24840	ebi-a-GCST0

exposure	mr_keep.ex	pval_origin.	data_source	action	mr_keep	reliability.ex	R2	F
|| id:ebi-a-(	TRUE	reported	igd	2	TRUE	high	0.000971	24.141645
|| id:ebi-a-(	TRUE	reported	igd	2	TRUE	high	0.0008572	21.310173
|| id:ebi-a-(	TRUE	reported	igd	2	TRUE	high	0.0007893	19.620926
|| id:ebi-a-(	TRUE	reported	igd	2	TRUE	high	0.0016338	40.647252
|| id:ebi-a-(	TRUE	reported	igd	2	TRUE	high	0.0015818	39.351121
|| id:ebi-a-(	TRUE	reported	igd	2	TRUE	high	0.0010419	25.906341
|| id:ebi-a-(	TRUE	reported	igd	2	TRUE	high	0.0012539	31.18259
|| id:ebi-a-(	TRUE	reported	igd	2	TRUE	high	0.0043982	109.72538
|| id:ebi-a-(	TRUE	reported	igd	2	TRUE	high	0.0018524	46.096309
|| id:ebi-a-(	TRUE	reported	igd	2	TRUE	high	0.0010037	24.956167
|| id:ebi-a-(	TRUE	reported	igd	2	TRUE	high	0.0013818	34.369843
|| id:ebi-a-(	TRUE	reported	igd	2	TRUE	high	0.0013488	33.546735
|| id:ebi-a-(	TRUE	reported	igd	2	TRUE	high	0.0009018	22.419126
|| id:ebi-a-(	TRUE	reported	igd	2	TRUE	high	0.0008842	21.981506
|| id:ebi-a-(	TRUE	reported	igd	2	TRUE	high	0.0008839	21.973402
|| id:ebi-a-(	TRUE	reported	igd	2	TRUE	high	0.0009241	22.974723
|| id:ebi-a-(	TRUE	reported	igd	2	TRUE	high	0.0011017	27.394192
|| id:ebi-a-(	TRUE	reported	igd	2	TRUE	high	0.0008747	21.74512
|| id:ebi-a-(	TRUE	reported	igd	2	TRUE	high	0.000898	22.324639
|| id:ebi-a-(	TRUE	reported	igd	2	TRUE	high	0.0008479	21.076805
|| id:ebi-a-(	TRUE	reported	igd	2	TRUE	high	0.0008634	21.463126
|| id:ebi-a-(	TRUE	reported	igd	2	TRUE	high	0.0007933	19.719652
|| id:ebi-a-(	TRUE	reported	igd	2	TRUE	high	0.0009249	22.994533
|| id:ebi-a-(	TRUE	reported	igd	2	TRUE	high	0.0008074	20.071222
|| id:ebi-a-(	TRUE	reported	igd	2	TRUE	high	0.002757	68.667511
|| id:ebi-a-(	TRUE	reported	igd	2	TRUE	high	0.000883	21.950689
|| id:ebi-a-(	TRUE	reported	igd	2	TRUE	high	0.0008956	22.264809
|| id:ebi-a-(	TRUE	reported	igd	2	TRUE	high	0.0010245	25.472963
|| id:ebi-a-(	TRUE	reported	igd	2	TRUE	high	0.000916	22.773564
|| id:ebi-a-(	TRUE	reported	igd	2	TRUE	high	0.0009508	23.639522
|| id:ebi-a-(	TRUE	reported	igd	2	TRUE	high	0.000814	20.233834
|| id:ebi-a-(	TRUE	reported	igd	2	TRUE	high	0.000913	22.698278
|| id:ebi-a-(	TRUE	reported	igd	2	TRUE	high	0.0009804	24.376241
|| id:ebi-a-(	TRUE	reported	igd	2	TRUE	high	0.0009833	24.446946
|| id:ebi-a-(	TRUE	reported	igd	2	TRUE	high	0.0010427	25.926782
|| id:ebi-a-(	TRUE	reported	igd	2	TRUE	high	0.0009068	22.544308
|| id:ebi-a-(	TRUE	reported	igd	2	TRUE	high	0.001041	25.884297
|| id:ebi-a-(	TRUE	reported	igd	2	TRUE	high	0.0008597	21.371119
|| id:ebi-a-(	TRUE	reported	igd	2	TRUE	high	0.000791	19.662553
|| id:ebi-a-(	TRUE	reported	igd	2	TRUE	high	0.0008934	22.209668
|| id:ebi-a-(	TRUE	reported	igd	2	TRUE	high	0.0011842	29.4485
|| id:ebi-a-(	TRUE	reported	igd	2	TRUE	high	0.0009276	23.061853
|| id:ebi-a-(	TRUE	reported	igd	2	TRUE	high	0.0009292	23.101393
|| id:ebi-a-(	TRUE	reported	igd	2	TRUE	high	0.0023863	59.413619
|| id:ebi-a-(	TRUE	reported	igd	2	TRUE	high	0.0009949	24.736789
|| id:ebi-a-(	TRUE	reported	igd	2	TRUE	high	0.000794	19.73739

|| id:ebi-a-(	TRUE	reported	igd	2	TRUE	high	0.0012864	31.991953
|| id:ebi-a-(	TRUE	reported	igd	2	TRUE	high	0.000996	24.763069
|| id:ebi-a-(	TRUE	reported	igd	2	TRUE	high	0.0008554	21.263665
|| id:ebi-a-(	TRUE	reported	igd	2	TRUE	high	0.000833	20.706593
|| id:ebi-a-(	TRUE	reported	igd	2	TRUE	high	0.0008051	20.013312
|| id:ebi-a-(	TRUE	reported	igd	2	TRUE	high	0.0008952	22.254151
|| id:ebi-a-(	TRUE	reported	igd	2	TRUE	high	0.0008173	20.31592
|| id:ebi-a-(	TRUE	reported	igd	2	TRUE	high	0.000932	23.170297
|| id:ebi-a-(	TRUE	reported	igd	2	TRUE	high	0.0007929	19.708777
|| id:ebi-a-(	TRUE	reported	igd	2	TRUE	high	0.0011459	28.49408
|| id:ebi-a-(	TRUE	reported	igd	2	TRUE	high	0.0007903	19.645371
|| id:ebi-a-(	TRUE	reported	igd	2	TRUE	high	0.0008189	20.356505
|| id:ebi-a-(	TRUE	reported	igd	2	TRUE	high	0.0008119	20.182341
|| id:ebi-a-(	TRUE	reported	igd	2	TRUE	high	0.0010412	25.888177
|| id:ebi-a-(	TRUE	reported	igd	2	TRUE	high	0.0009662	24.021401
|| id:ebi-a-(	TRUE	reported	igd	2	TRUE	high	0.0008255	20.519984
|| id:ebi-a-(	TRUE	reported	igd	2	TRUE	high	0.0009245	22.983713
|| id:ebi-a-(	TRUE	reported	igd	2	TRUE	high	0.0007987	19.854753
|| id:ebi-a-(	TRUE	reported	igd	2	TRUE	high	0.0007919	19.6839
|| id:ebi-a-(	TRUE	reported	igd	2	TRUE	high	0.000857	21.30439
|| id:ebi-a-(	TRUE	reported	igd	2	TRUE	high	0.0008493	21.113926
|| id:ebi-a-(	TRUE	reported	igd	2	TRUE	high	0.0010748	26.72555
|| id:ebi-a-(	TRUE	reported	igd	2	TRUE	high	0.0009221	22.923515
|| id:ebi-a-(	TRUE	reported	igd	2	TRUE	high	0.0008526	21.195989
|| id:ebi-a-(	TRUE	reported	igd	2	TRUE	high	0.0010245	25.473746
|| id:ebi-a-(	TRUE	reported	igd	2	TRUE	high	0.0013797	34.315849
|| id:ebi-a-(	TRUE	reported	igd	2	TRUE	high	0.0054968	137.28521
|| id:ebi-a-(	TRUE	reported	igd	2	TRUE	high	0.0009929	24.687059
|| id:ebi-a-(	TRUE	reported	igd	2	TRUE	high	0.0011789	29.315163
|| id:ebi-a-(	TRUE	reported	igd	2	TRUE	high	0.0008021	19.939744
|| id:ebi-a-(	TRUE	reported	igd	2	TRUE	high	0.0009849	24.487838
|| id:ebi-a-(	TRUE	reported	igd	2	TRUE	high	0.001738	43.243032
|| id:ebi-a-(	TRUE	reported	igd	2	TRUE	high	0.0008367	20.799535
|| id:ebi-a-(	TRUE	reported	igd	2	TRUE	high	0.0008218	20.427981
|| id:ebi-a-(	TRUE	reported	igd	2	TRUE	high	0.001658	41.249772
|| id:ebi-a-(	TRUE	reported	igd	2	TRUE	high	0.0013661	33.976621
|| id:ebi-a-(	TRUE	reported	igd	2	TRUE	high	0.0015176	37.752224
|| id:ebi-a-(	TRUE	reported	igd	2	TRUE	high	0.003051	76.012579
|| id:ebi-a-(	TRUE	reported	igd	2	TRUE	high	0.0016252	40.431984
|| id:ebi-a-(	TRUE	reported	igd	2	TRUE	high	0.0008471	21.05707
|| id:ebi-a-(	TRUE	reported	igd	2	TRUE	high	0.0013493	33.5587
|| id:ebi-a-(	TRUE	reported	igd	2	TRUE	high	0.0009177	22.814643
|| id:ebi-a-(	TRUE	reported	igd	2	TRUE	high	0.0014678	36.510154
|| id:ebi-a-(	TRUE	reported	igd	2	TRUE	high	0.0009357	23.261697
|| id:ebi-a-(	TRUE	reported	igd	2	TRUE	high	0.0008712	21.658051
|| id:ebi-a-(	TRUE	reported	igd	2	TRUE	high	0.0010504	26.117654
|| id:ebi-a-(	TRUE	reported	igd	2	TRUE	high	0.0010699	26.601736

<table>
<tr><td></td><td></td><td></td><td></td><td></td><td></td><td></td><td></td><td></td></tr>
<tr><td>|| id:ebi-a-(</td><td>TRUE</td><td>reported</td><td>igd</td><td>2</td><td>TRUE</td><td>high</td><td>0.0008339</td><td>20.730457</td></tr>
<tr><td>|| id:ebi-a-(</td><td>TRUE</td><td>reported</td><td>igd</td><td>2</td><td>TRUE</td><td>high</td><td>0.0008592</td><td>21.358753</td></tr>
<tr><td>|| id:ebi-a-(</td><td>TRUE</td><td>reported</td><td>igd</td><td>2</td><td>TRUE</td><td>high</td><td>0.0009819</td><td>24.413259</td></tr>
<tr><td>|| id:ebi-a-(</td><td>TRUE</td><td>reported</td><td>igd</td><td>2</td><td>TRUE</td><td>high</td><td>0.0008391</td><td>20.860139</td></tr>
<tr><td>|| id:ebi-a-(</td><td>TRUE</td><td>reported</td><td>igd</td><td>2</td><td>TRUE</td><td>high</td><td>0.0012916</td><td>32.12143</td></tr>
<tr><td>|| id:ebi-a-(</td><td>TRUE</td><td>reported</td><td>igd</td><td>2</td><td>TRUE</td><td>high</td><td>0.0015571</td><td>38.735806</td></tr>
<tr><td>|| id:ebi-a-(</td><td>TRUE</td><td>reported</td><td>igd</td><td>2</td><td>TRUE</td><td>high</td><td>0.0008335</td><td>20.719495</td></tr>
<tr><td>|| id:ebi-a-(</td><td>TRUE</td><td>reported</td><td>igd</td><td>2</td><td>TRUE</td><td>high</td><td>0.0007922</td><td>19.691795</td></tr>
<tr><td>|| id:ebi-a-(</td><td>TRUE</td><td>reported</td><td>igd</td><td>2</td><td>TRUE</td><td>high</td><td>0.0008288</td><td>20.602541</td></tr>
<tr><td>|| id:ebi-a-(</td><td>TRUE</td><td>reported</td><td>igd</td><td>2</td><td>TRUE</td><td>high</td><td>0.0009705</td><td>24.128559</td></tr>
<tr><td>|| id:ebi-a-(</td><td>TRUE</td><td>reported</td><td>igd</td><td>2</td><td>TRUE</td><td>high</td><td>0.0009761</td><td>24.267035</td></tr>
<tr><td>|| id:ebi-a-(</td><td>TRUE</td><td>reported</td><td>igd</td><td>2</td><td>TRUE</td><td>high</td><td>0.000933</td><td>23.195134</td></tr>
<tr><td>|| id:ebi-a-(</td><td>TRUE</td><td>reported</td><td>igd</td><td>2</td><td>TRUE</td><td>high</td><td>0.0009726</td><td>24.181967</td></tr>
<tr><td>|| id:ebi-a-(</td><td>TRUE</td><td>reported</td><td>igd</td><td>2</td><td>TRUE</td><td>high</td><td>0.0007978</td><td>19.831962</td></tr>
<tr><td>|| id:ebi-a-(</td><td>TRUE</td><td>reported</td><td>igd</td><td>2</td><td>TRUE</td><td>high</td><td>0.0008314</td><td>20.668711</td></tr>
<tr><td>|| id:ebi-a-(</td><td>TRUE</td><td>reported</td><td>igd</td><td>2</td><td>TRUE</td><td>high</td><td>0.0019032</td><td>47.362968</td></tr>
<tr><td>|| id:ebi-a-(</td><td>TRUE</td><td>reported</td><td>igd</td><td>2</td><td>TRUE</td><td>high</td><td>0.0011292</td><td>28.079051</td></tr>
<tr><td>|| id:ebi-a-(</td><td>TRUE</td><td>reported</td><td>igd</td><td>2</td><td>TRUE</td><td>high</td><td>0.001064</td><td>26.455649</td></tr>
<tr><td>|| id:ebi-a-(</td><td>TRUE</td><td>reported</td><td>igd</td><td>2</td><td>TRUE</td><td>high</td><td>0.0219063</td><td>556.29491</td></tr>
<tr><td>|| id:ebi-a-(</td><td>TRUE</td><td>reported</td><td>igd</td><td>2</td><td>TRUE</td><td>high</td><td>0.0011657</td><td>28.986356</td></tr>
<tr><td>|| id:ebi-a-(</td><td>TRUE</td><td>reported</td><td>igd</td><td>2</td><td>TRUE</td><td>high</td><td>0.0010146</td><td>25.226336</td></tr>
<tr><td>|| id:ebi-a-(</td><td>TRUE</td><td>reported</td><td>igd</td><td>2</td><td>TRUE</td><td>high</td><td>0.001228</td><td>30.537707</td></tr>
<tr><td>|| id:ebi-a-(</td><td>TRUE</td><td>reported</td><td>igd</td><td>2</td><td>TRUE</td><td>high</td><td>0.0008058</td><td>20.03006</td></tr>
<tr><td>|| id:ebi-a-(</td><td>TRUE</td><td>reported</td><td>igd</td><td>2</td><td>TRUE</td><td>high</td><td>0.001556</td><td>38.708913</td></tr>
<tr><td>|| id:ebi-a-(</td><td>TRUE</td><td>reported</td><td>igd</td><td>2</td><td>TRUE</td><td>high</td><td>0.0010054</td><td>24.997987</td></tr>
<tr><td>|| id:ebi-a-(</td><td>TRUE</td><td>reported</td><td>igd</td><td>2</td><td>TRUE</td><td>high</td><td>0.0010069</td><td>25.034494</td></tr>
<tr><td>|| id:ebi-a-(</td><td>TRUE</td><td>reported</td><td>igd</td><td>2</td><td>TRUE</td><td>high</td><td>0.000904</td><td>22.472813</td></tr>
<tr><td>|| id:ebi-a-(</td><td>TRUE</td><td>reported</td><td>igd</td><td>2</td><td>TRUE</td><td>high</td><td>0.0015078</td><td>37.507449</td></tr>
<tr><td>|| id:ebi-a-(</td><td>TRUE</td><td>reported</td><td>igd</td><td>2</td><td>TRUE</td><td>high</td><td>0.0009446</td><td>23.484977</td></tr>
<tr><td>|| id:ebi-a-(</td><td>TRUE</td><td>reported</td><td>igd</td><td>2</td><td>TRUE</td><td>high</td><td>0.0011703</td><td>29.102999</td></tr>
<tr><td>|| id:ebi-a-(</td><td>TRUE</td><td>reported</td><td>igd</td><td>2</td><td>TRUE</td><td>high</td><td>0.0009192</td><td>22.850873</td></tr>
<tr><td>|| id:ebi-a-(</td><td>TRUE</td><td>reported</td><td>igd</td><td>2</td><td>TRUE</td><td>high</td><td>0.0020378</td><td>50.718105</td></tr>
<tr><td>|| id:ebi-a-(</td><td>TRUE</td><td>reported</td><td>igd</td><td>2</td><td>TRUE</td><td>high</td><td>0.0008108</td><td>20.15589</td></tr>
<tr><td>|| id:ebi-a-(</td><td>TRUE</td><td>reported</td><td>igd</td><td>2</td><td>TRUE</td><td>high</td><td>0.0008742</td><td>21.73123</td></tr>
<tr><td>|| id:ebi-a-(</td><td>TRUE</td><td>reported</td><td>igd</td><td>2</td><td>TRUE</td><td>high</td><td>0.0010182</td><td>25.316891</td></tr>
<tr><td>|| id:ebi-a-(</td><td>TRUE</td><td>reported</td><td>igd</td><td>2</td><td>TRUE</td><td>high</td><td>0.0010877</td><td>27.044938</td></tr>
<tr><td>|| id:ebi-a-(</td><td>TRUE</td><td>reported</td><td>igd</td><td>2</td><td>TRUE</td><td>high</td><td>0.0141236</td><td>355.82795</td></tr>
<tr><td>|| id:ebi-a-(</td><td>TRUE</td><td>reported</td><td>igd</td><td>2</td><td>TRUE</td><td>high</td><td>0.0012469</td><td>31.007948</td></tr>
<tr><td>|| id:ebi-a-(</td><td>TRUE</td><td>reported</td><td>igd</td><td>2</td><td>TRUE</td><td>high</td><td>0.0007895</td><td>19.626225</td></tr>
<tr><td>|| id:ebi-a-(</td><td>TRUE</td><td>reported</td><td>igd</td><td>2</td><td>TRUE</td><td>high</td><td>0.0155147</td><td>391.427</td></tr>
<tr><td>|| id:ebi-a-(</td><td>TRUE</td><td>reported</td><td>igd</td><td>2</td><td>TRUE</td><td>high</td><td>0.0008407</td><td>20.900107</td></tr>
<tr><td>|| id:ebi-a-(</td><td>TRUE</td><td>reported</td><td>igd</td><td>2</td><td>TRUE</td><td>high</td><td>0.0011244</td><td>27.960469</td></tr>
<tr><td>|| id:ebi-a-(</td><td>TRUE</td><td>reported</td><td>igd</td><td>2</td><td>TRUE</td><td>high</td><td>0.0007855</td><td>19.526113</td></tr>
<tr><td>|| id:ebi-a-(</td><td>TRUE</td><td>reported</td><td>igd</td><td>2</td><td>TRUE</td><td>high</td><td>0.0010226</td><td>25.425295</td></tr>
<tr><td>|| id:ebi-a-(</td><td>TRUE</td><td>reported</td><td>igd</td><td>2</td><td>TRUE</td><td>high</td><td>0.0008559</td><td>21.276547</td></tr>
<tr><td>|| id:ebi-a-(</td><td>TRUE</td><td>reported</td><td>igd</td><td>2</td><td>TRUE</td><td>high</td><td>0.0010634</td><td>26.441122</td></tr>
<tr><td>|| id:ebi-a-(</td><td>TRUE</td><td>reported</td><td>igd</td><td>2</td><td>TRUE</td><td>high</td><td>0.0009679</td><td>24.064907</td></tr>
</table>

|| id:ebi-a-(	TRUE	reported	igd	2	TRUE	high	0.0010559	26.25416
|| id:ebi-a-(	TRUE	reported	igd	2	TRUE	high	0.0008168	20.303587
|| id:ebi-a-(	TRUE	reported	igd	2	TRUE	high	0.0008527	21.198506
|| id:ebi-a-(	TRUE	reported	igd	2	TRUE	high	0.0009003	22.382582
|| id:ebi-a-(	TRUE	reported	igd	2	TRUE	high	0.0024959	62.148051
|| id:ebi-a-(	TRUE	reported	igd	2	TRUE	high	0.0008384	20.840934
|| id:ebi-a-(	TRUE	reported	igd	2	TRUE	high	0.0008331	20.711004
|| id:ebi-a-(	TRUE	reported	igd	2	TRUE	high	0.0008353	20.765171
|| id:ebi-a-(	TRUE	reported	igd	2	TRUE	high	0.0010131	25.188531
|| id:ebi-a-(	TRUE	reported	igd	2	TRUE	high	0.0019731	49.105238
|| id:ebi-a-(	TRUE	reported	igd	2	TRUE	high	0.0008829	21.948374
|| id:ebi-a-(	TRUE	reported	igd	2	TRUE	high	0.0009029	22.446183
|| id:ebi-a-(	TRUE	reported	igd	2	TRUE	high	0.0008452	21.011672
|| id:ebi-a-(	TRUE	reported	igd	2	TRUE	high	0.0009587	23.835451
|| id:ebi-a-(	TRUE	reported	igd	2	TRUE	high	0.0007989	19.858987
|| id:ebi-a-(	TRUE	reported	igd	2	TRUE	high	0.0008386	20.847517
|| id:ebi-a-(	TRUE	reported	igd	2	TRUE	high	0.0008958	22.269983
|| id:ebi-a-(	TRUE	reported	igd	2	TRUE	high	0.0009474	23.554909
|| id:ebi-a-(	TRUE	reported	igd	2	TRUE	high	0.0010801	26.856896
|| id:ebi-a-(	TRUE	reported	igd	2	TRUE	high	0.000986	24.515112
|| id:ebi-a-(	TRUE	reported	igd	2	TRUE	high	0.0007863	19.546553
|| id:ebi-a-(	TRUE	reported	igd	2	TRUE	high	0.0008001	19.889772
|| id:ebi-a-(	TRUE	reported	igd	2	TRUE	high	0.0010467	26.025923
|| id:ebi-a-(	TRUE	reported	igd	2	TRUE	high	0.0007876	19.577091
|| id:ebi-a-(	TRUE	reported	igd	2	TRUE	high	0.0008261	20.535877
|| id:ebi-a-(	TRUE	reported	igd	2	TRUE	high	0.0011123	27.65891
|| id:ebi-a-(	TRUE	reported	igd	2	TRUE	high	0.0016277	40.495395
|| id:ebi-a-(	TRUE	reported	igd	2	TRUE	high	0.0008816	21.917657
|| id:ebi-a-(	TRUE	reported	igd	2	TRUE	high	0.0010621	26.407293
|| id:ebi-a-(	TRUE	reported	igd	2	TRUE	high	0.0008654	21.51319
|| id:ebi-a-(	TRUE	reported	igd	2	TRUE	high	0.0557096	1465.3504
|| id:ebi-a-(	TRUE	reported	igd	2	TRUE	high	0.0011388	28.316813
|| id:ebi-a-(	TRUE	reported	igd	2	TRUE	high	0.0015179	37.757936
|| id:ebi-a-(	TRUE	reported	igd	2	TRUE	high	0.0018934	47.116287
|| id:ebi-a-(	TRUE	reported	igd	2	TRUE	high	0.0010342	25.714286

SNP	effect_allel	other_allel	effect_allel	other_allel	beta.expos	beta.outco	eaf.exposu	eaf.outcom
rs1125519(	C	T	C	T	0.7636	0	0.1531	
rs1128226(	T	C	T	C	1.9887	-0.0202	0.0179	
rs1197075:	C	T	C	T	2.6634	0.040822	0.0298	
rs1212586:	T	C	T	C	1.43	0.04879	0.0368	
rs1467516(	T	C	T	C	1.5969	0.029559	0.0318	
rs2214447	T	C	T	C	-0.9206	-0.03046	0.8608	
rs2316776	T	C	T	C	1.1729	0	0.0557	
rs9367584	T	C	T	C	1.6945	0.00995	0.0298	
rs9863260	C	A	C	A	-0.6446	0	0.664	

remove	palindromi	ambiguous	id.outcome	chr	pos	se.outcom	samplesize	pval.outcor
FALSE	FALSE	FALSE	ebi-a-GCST	10	7617096	0.02617	57284	0.93
FALSE	FALSE	FALSE	ebi-a-GCST	11	97949762	0.099059	57284	0.84
FALSE	FALSE	FALSE	ebi-a-GCST	6	139809258	0.068128	57284	0.52
FALSE	FALSE	FALSE	ebi-a-GCST	1	112354879	0.063859	57284	0.450001
FALSE	FALSE	FALSE	ebi-a-GCST	12	21468629	0.065023	57284	0.61
FALSE	FALSE	FALSE	ebi-a-GCST	7	110828789	0.025644	57284	0.22
FALSE	FALSE	FALSE	ebi-a-GCST	17	43996933	0.024893	57284	0.93
FALSE	FALSE	FALSE	ebi-a-GCST	6	54437578	0.029443	57284	0.81
FALSE	FALSE	FALSE	ebi-a-GCST	3	143420702	0.020828	57284	0.86

outcome	originalnan	outcome.d	mr_keep.o	data_sourc	pos.exposu	chr.exposu	se.exposur	pval.exposu
Rheumatoi	Rheumatoi	Rheumatoi	TRUE	igd	7617096	10	0.1653	3.85E-06
Rheumatoi	Rheumatoi	Rheumatoi	TRUE	igd	97949762	11	0.4435	7.31E-06
Rheumatoi	Rheumatoi	Rheumatoi	TRUE	igd	1.4E+08	6	0.5649	2.42E-06
Rheumatoi	Rheumatoi	Rheumatoi	TRUE	igd	1.12E+08	1	0.3108	4.21E-06
Rheumatoi	Rheumatoi	Rheumatoi	TRUE	igd	21468629	12	0.3281	1.13E-06
Rheumatoi	Rheumatoi	Rheumatoi	TRUE	igd	1.11E+08	7	0.2007	4.51E-06
Rheumatoi	Rheumatoi	Rheumatoi	TRUE	igd	43996933	17	0.2624	7.84E-06
Rheumatoi	Rheumatoi	Rheumatoi	TRUE	igd	54437578	6	0.3755	6.4E-06
Rheumatoi	Rheumatoi	Rheumatoi	TRUE	igd	1.43E+08	3	0.1451	8.96E-06

samplesize	id.exposure	exposure	mr_keep.e	pval_origin	data_sourc	action	mr_keep	reliability.e
162964	finn-b-I9_H	|| id:finn-b	TRUE	reported	igd	2	TRUE	high
162964	finn-b-I9_H	|| id:finn-b	TRUE	reported	igd	2	TRUE	high
162964	finn-b-I9_H	|| id:finn-b	TRUE	reported	igd	2	TRUE	high
162964	finn-b-I9_H	|| id:finn-b	TRUE	reported	igd	2	TRUE	high
162964	finn-b-I9_H	|| id:finn-b	TRUE	reported	igd	2	TRUE	high
162964	finn-b-I9_H	|| id:finn-b	TRUE	reported	igd	2	TRUE	high
162964	finn-b-I9_H	|| id:finn-b	TRUE	reported	igd	2	TRUE	high
162964	finn-b-I9_H	|| id:finn-b	TRUE	reported	igd	2	TRUE	high
162964	finn-b-I9_H	|| id:finn-b	TRUE	reported	igd	2	TRUE	high

R2	F
0.000131	21.33933
0.000123	20.10694
0.000136	22.22922
0.00013	21.16921
0.000145	23.6885
0.000129	21.03981
0.000123	19.9797
0.000125	20.36376
0.000121	19.73515

SNP	effect_allel	other_allel	effect_allel	other_allel	beta.expos	beta.outco	eaf.exposu	eaf.outcom
rs1125519	C	T	C	T	0.7636	-0.0499	0.1531	0.2048
rs1128226	T	C	T	C	1.9887	0.0292	0.0179	0.03033
rs1140952	T	A	T	A	1.4095	-0.0911	0.0606	0.05336
rs1197075	C	T	C	T	2.6634	0.2115	0.0298	0.01892
rs1212586	T	C	T	C	1.43	-0.0311	0.0368	0.05697
rs1467516	T	C	T	C	1.5969	0.0158	0.0318	0.05011
rs2214447	T	C	T	C	-0.9206	0.0052	0.8608	0.8689
rs2316776	T	C	T	C	1.1729	0.0076	0.0557	0.07634
rs3490259	A	T	A	T	1.0688	0.0036	0.1243	0.09589
rs5590957	C	T	C	T	1.176	-0.0326	0.0666	0.09875
rs9367584	T	C	T	C	1.6945	-0.0188	0.0298	0.03945
rs9863260	C	A	C	A	-0.6446	0.035	0.664	0.7105

remove	palindromi	ambiguous	id.outcome	chr	pos	se.outcome	samplesize	pval.outcor
FALSE	FALSE	FALSE	finn-b-K11_	10	7617096	0.0303		0.09972
FALSE	FALSE	FALSE	finn-b-K11_	11	97949762	0.0712		0.681601
FALSE	TRUE	FALSE	finn-b-K11_	2	203433118	0.0547		0.096119
FALSE	FALSE	FALSE	finn-b-K11_	6	139809258	0.0891		0.01765
FALSE	FALSE	FALSE	finn-b-K11_	1	112354879	0.0531		0.5582
FALSE	FALSE	FALSE	finn-b-K11_	12	21468629	0.0564		0.778999
FALSE	FALSE	FALSE	finn-b-K11_	7	110828789	0.036		0.8859
FALSE	FALSE	FALSE	finn-b-K11_	17	43996933	0.0459		0.8681
FALSE	TRUE	FALSE	finn-b-K11_	4	48671322	0.0415		0.9303
FALSE	FALSE	FALSE	finn-b-K11_	5	180648732	0.042		0.4377
FALSE	FALSE	FALSE	finn-b-K11_	6	54437578	0.0625		0.763901
FALSE	FALSE	FALSE	finn-b-K11_	3	143420702	0.0273		0.1996

outcome	originalnan	outcome.d	mr_keep.o	data_sourc	pos.exposu	chr.exposu	se.exposur	pval.exposu
Inflammatc	Inflammatc	Inflammatc	TRUE	igd	7617096	10	0.1653	3.85E-06
Inflammatc	Inflammatc	Inflammatc	TRUE	igd	97949762	11	0.4435	7.31E-06
Inflammatc	Inflammatc	Inflammatc	TRUE	igd	2.03E+08	2	0.3181	9.37E-06
Inflammatc	Inflammatc	Inflammatc	TRUE	igd	1.4E+08	6	0.5649	2.42E-06
Inflammatc	Inflammatc	Inflammatc	TRUE	igd	1.12E+08	1	0.3108	4.21E-06
Inflammatc	Inflammatc	Inflammatc	TRUE	igd	21468629	12	0.3281	1.13E-06
Inflammatc	Inflammatc	Inflammatc	TRUE	igd	1.11E+08	7	0.2007	4.51E-06
Inflammatc	Inflammatc	Inflammatc	TRUE	igd	43996933	17	0.2624	7.84E-06
Inflammatc	Inflammatc	Inflammatc	TRUE	igd	48671322	4	0.2362	6.06E-06
Inflammatc	Inflammatc	Inflammatc	TRUE	igd	1.81E+08	5	0.243	1.3E-06
Inflammatc	Inflammatc	Inflammatc	TRUE	igd	54437578	6	0.3755	6.4E-06
Inflammatc	Inflammatc	Inflammatc	TRUE	igd	1.43E+08	3	0.1451	8.96E-06

samplesize	id.exposur	exposure	mr_keep.e	pval_origin	data_sourc	action	mr_keep	reliability.e
162964	finn-b-I9_H	|| id:finn-	TRUE	reported	igd	2	TRUE	high
162964	finn-b-I9_H	|| id:finn-	TRUE	reported	igd	2	TRUE	high
162964	finn-b-I9_H	|| id:finn-	TRUE	reported	igd	2	TRUE	high
162964	finn-b-I9_H	|| id:finn-	TRUE	reported	igd	2	TRUE	high
162964	finn-b-I9_H	|| id:finn-	TRUE	reported	igd	2	TRUE	high
162964	finn-b-I9_H	|| id:finn-	TRUE	reported	igd	2	TRUE	high
162964	finn-b-I9_H	|| id:finn-	TRUE	reported	igd	2	TRUE	high
162964	finn-b-I9_H	|| id:finn-	TRUE	reported	igd	2	TRUE	high
162964	finn-b-I9_H	|| id:finn-	TRUE	reported	igd	2	TRUE	high
162964	finn-b-I9_H	|| id:finn-	TRUE	reported	igd	2	TRUE	high
162964	finn-b-I9_H	|| id:finn-	TRUE	reported	igd	2	TRUE	high
162964	finn-b-I9_H	|| id:finn-	TRUE	reported	igd	2	TRUE	high

R2	F
0.000131	21.33933
0.000123	20.10694
0.00012	19.63349
0.000136	22.22922
0.00013	21.16921
0.000145	23.6885
0.000129	21.03981
0.000123	19.9797
0.000126	20.47518
0.000144	23.42053
0.000125	20.36376
0.000121	19.73515

SNP	effect_allel	other_allel	effect_allel	other_allel	beta.expos	beta.outco	eaf.exposu	eaf.outcom
rs1125519(	C	T	C	T	0.7636	-0.00489	0.1531	
rs1197075:	C	T	C	T	2.6634	-0.01568	0.0298	
rs1212586:	T	C	T	C	1.43	-0.06644	0.0368	
rs2214447	T	C	T	C	-0.9206	0.034981	0.8608	
rs2316776	T	C	T	C	1.1729	0.038429	0.0557	
rs9367584	T	C	T	C	1.6945	0.042485	0.0298	
rs9863260	C	A	C	A	-0.6446	0.012478	0.664	

remove	palindromi	ambiguous	id.outcome	chr	pos	se.outcome	samplesize	pval.outcor
FALSE	FALSE	FALSE	ieu-b-18	10	7617096	0.023154	115803	0.8328
FALSE	FALSE	FALSE	ieu-b-18	6	139809258	0.087617	115803	0.858
FALSE	FALSE	FALSE	ieu-b-18	1	112354879	0.059859	115803	0.267
FALSE	FALSE	FALSE	ieu-b-18	7	110828789	0.023608	115803	0.1384
FALSE	FALSE	FALSE	ieu-b-18	17	43996933	0.035432	115803	0.2781
FALSE	FALSE	FALSE	ieu-b-18	6	54437578	0.041494	115803	0.3059
FALSE	FALSE	FALSE	ieu-b-18	3	143420702	0.017682	115803	0.4804

outcome	originalnan	outcome.d	mr_keep.o	data_sourc	pos.exposu	chr.exposu	se.exposur	pval.exposu
multiple sc	multiple sc	multiple sc	TRUE	igd	7617096	10	0.1653	3.85E-06
multiple sc	multiple sc	multiple sc	TRUE	igd	1.4E+08	6	0.5649	2.42E-06
multiple sc	multiple sc	multiple sc	TRUE	igd	1.12E+08	1	0.3108	4.21E-06
multiple sc	multiple sc	multiple sc	TRUE	igd	1.11E+08	7	0.2007	4.51E-06
multiple sc	multiple sc	multiple sc	TRUE	igd	43996933	17	0.2624	7.84E-06
multiple sc	multiple sc	multiple sc	TRUE	igd	54437578	6	0.3755	6.4E-06
multiple sc	multiple sc	multiple sc	TRUE	igd	1.43E+08	3	0.1451	8.96E-06

<table>
<tr><th>samplesize</th><th>id.exposur</th><th>exposure</th><th>mr_keep.e</th><th>pval_origin</th><th>data_sourc</th><th>action</th><th>mr_keep</th><th>reliability.e</th></tr>
<tr><td>162964</td><td>finn-b-I9_H</td><td>|| id:finn-</td><td>TRUE</td><td>reported</td><td>igd</td><td>2</td><td>TRUE</td><td>high</td></tr>
<tr><td>162964</td><td>finn-b-I9_H</td><td>|| id:finn-</td><td>TRUE</td><td>reported</td><td>igd</td><td>2</td><td>TRUE</td><td>high</td></tr>
<tr><td>162964</td><td>finn-b-I9_H</td><td>|| id:finn-</td><td>TRUE</td><td>reported</td><td>igd</td><td>2</td><td>TRUE</td><td>high</td></tr>
<tr><td>162964</td><td>finn-b-I9_H</td><td>|| id:finn-</td><td>TRUE</td><td>reported</td><td>igd</td><td>2</td><td>TRUE</td><td>high</td></tr>
<tr><td>162964</td><td>finn-b-I9_H</td><td>|| id:finn-</td><td>TRUE</td><td>reported</td><td>igd</td><td>2</td><td>TRUE</td><td>high</td></tr>
<tr><td>162964</td><td>finn-b-I9_H</td><td>|| id:finn-</td><td>TRUE</td><td>reported</td><td>igd</td><td>2</td><td>TRUE</td><td>high</td></tr>
<tr><td>162964</td><td>finn-b-I9_H</td><td>|| id:finn-</td><td>TRUE</td><td>reported</td><td>igd</td><td>2</td><td>TRUE</td><td>high</td></tr>
</table>

R2	F
0.000131	21.33933
0.000136	22.22922
0.00013	21.16921
0.000129	21.03981
0.000123	19.9797
0.000125	20.36376
0.000121	19.73515

SNP	effect_allel	other_allel	effect_allel	other_allel	beta.expos	beta.outco	eaf.exposu	eaf.outcom
rs11255196	C	T	C	T	0.7636	0.019803	0.1531	
rs11282268	T	C	T	C	1.9887	-0.17435	0.0179	
rs11970753	C	T	C	T	2.6634	0.086178	0.0298	
rs12125862	T	C	T	C	1.43	-0.12783	0.0368	
rs14675166	T	C	T	C	1.5969	-0.08338	0.0318	
rs2214447	T	C	T	C	-0.9206	-0.03922	0.8608	
rs2316776	T	C	T	C	1.1729	0.019803	0.0557	
rs9367584	T	C	T	C	1.6945	-0.08338	0.0298	
rs9863260	C	A	C	A	-0.6446	-0.06766	0.664	

remove	palindromi	ambiguous	id.outcome	chr	pos	se.outcome	samplesize	pval.outcor
FALSE	FALSE	FALSE	ebi-a-GCST	10	7617096	0.046946	14267	0.673161
FALSE	FALSE	FALSE	ebi-a-GCST	11	97949762	0.161477	14267	0.280257
FALSE	FALSE	FALSE	ebi-a-GCST	6	139809258	0.075092	14267	0.251121
FALSE	FALSE	FALSE	ebi-a-GCST	1	112354879	0.087881	14267	0.145775
FALSE	FALSE	FALSE	ebi-a-GCST	12	21468629	0.088743	14267	0.347431
FALSE	FALSE	FALSE	ebi-a-GCST	7	110828789	0.034902	14267	0.261126
FALSE	FALSE	FALSE	ebi-a-GCST	17	43996933	0.062772	14267	0.752404
FALSE	FALSE	FALSE	ebi-a-GCST	6	54437578	0.079288	14267	0.292967
FALSE	FALSE	FALSE	ebi-a-GCST	3	143420702	0.032527	14267	0.03752

outcome	originalnan	outcome.d	mr_keep.o	data_sourc	pos.exposu	chr.exposu	se.exposur	pval.exposu
Systemic lu	Systemic lu	Systemic lu	TRUE	igd	7617096	10	0.1653	3.85E-06
Systemic lu	Systemic lu	Systemic lu	TRUE	igd	97949762	11	0.4435	7.31E-06
Systemic lu	Systemic lu	Systemic lu	TRUE	igd	1.4E+08	6	0.5649	2.42E-06
Systemic lu	Systemic lu	Systemic lu	TRUE	igd	1.12E+08	1	0.3108	4.21E-06
Systemic lu	Systemic lu	Systemic lu	TRUE	igd	21468629	12	0.3281	1.13E-06
Systemic lu	Systemic lu	Systemic lu	TRUE	igd	1.11E+08	7	0.2007	4.51E-06
Systemic lu	Systemic lu	Systemic lu	TRUE	igd	43996933	17	0.2624	7.84E-06
Systemic lu	Systemic lu	Systemic lu	TRUE	igd	54437578	6	0.3755	6.4E-06
Systemic lu	Systemic lu	Systemic lu	TRUE	igd	1.43E+08	3	0.1451	8.96E-06

samplesize	id.exposure	exposure	mr_keep.e:	pval_origin	data_sourc	action	mr_keep	reliability.e
162964	finn-b-I9_H	|| id:finn-l	TRUE	reported	igd	2	TRUE	high
162964	finn-b-I9_H	|| id:finn-l	TRUE	reported	igd	2	TRUE	high
162964	finn-b-I9_H	|| id:finn-l	TRUE	reported	igd	2	TRUE	high
162964	finn-b-I9_H	|| id:finn-l	TRUE	reported	igd	2	TRUE	high
162964	finn-b-I9_H	|| id:finn-l	TRUE	reported	igd	2	TRUE	high
162964	finn-b-I9_H	|| id:finn-l	TRUE	reported	igd	2	TRUE	high
162964	finn-b-I9_H	|| id:finn-l	TRUE	reported	igd	2	TRUE	high
162964	finn-b-I9_H	|| id:finn-l	TRUE	reported	igd	2	TRUE	high
162964	finn-b-I9_H	|| id:finn-l	TRUE	reported	igd	2	TRUE	high

R2	F
0.000131	21.33933
0.000123	20.10694
0.000136	22.22922
0.00013	21.16921
0.000145	23.6885
0.000129	21.03981
0.000123	19.9797
0.000125	20.36376
0.000121	19.73515

SNP	effect_allel	other_allel	effect_allel	other_allel	beta.expos	beta.outco	eaf.exposu	eaf.outcom
rs1125519	C	T	C	T	0.7636	0.0027	0.1531	0.1801
rs1128226	T	C	T	C	1.9887	0.1535	0.0179	0.0176
rs1140952	T	A	T	A	1.4095	-0.027	0.0606	0.0606
rs1197075	C	T	C	T	2.6634	-0.0318	0.0298	0.0324
rs1212586	T	C	T	C	1.43	0.0951	0.0368	0.0361
rs1467516	T	C	T	C	1.5969	-0.0055	0.0318	0.0357
rs2214447	T	C	T	C	-0.9206	-0.0326	0.8608	0.8677
rs2316776	T	C	T	C	1.1729	0.0619	0.0557	0.058
rs3490259	A	T	A	T	1.0688	-0.0571	0.1243	0.1313
rs5590957	C	T	C	T	1.176	0.0817	0.0666	0.078
rs9367584	T	C	T	C	1.6945	0.0419	0.0298	0.0385
rs9863260	C	A	C	A	-0.6446	0.0006	0.664	0.6726

remove	palindromi	ambiguous	id.outcome	chr	pos	se.outcome	samplesize	pval.outcor
FALSE	FALSE	FALSE	ebi-a-GCST	10	7617096	0.0313	24840	0.9316
FALSE	FALSE	FALSE	ebi-a-GCST	11	97949762	0.0882	24840	0.081999
FALSE	TRUE	FALSE	ebi-a-GCST	2	203433118	0.0512	24840	0.5987
FALSE	FALSE	FALSE	ebi-a-GCST	6	139809258	0.0711	24840	0.6546
FALSE	FALSE	FALSE	ebi-a-GCST	1	112354879	0.0656	24840	0.147
FALSE	FALSE	FALSE	ebi-a-GCST	12	21468629	0.0654	24840	0.9325
FALSE	FALSE	FALSE	ebi-a-GCST	7	110828789	0.0345	24840	0.344
FALSE	FALSE	FALSE	ebi-a-GCST	17	43996933	0.0493	24840	0.2094
FALSE	TRUE	FALSE	ebi-a-GCST	4	48671322	0.0366	24840	0.1181
FALSE	FALSE	FALSE	ebi-a-GCST	5	180648732	0.0479	24840	0.088111
FALSE	FALSE	FALSE	ebi-a-GCST	6	54437578	0.0595	24840	0.4807
FALSE	FALSE	FALSE	ebi-a-GCST	3	143420702	0.0253	24840	0.9821

outcome	originalnan	outcome.d	mr_keep.o	data_sourc	pos.exposu	chr.exposu	se.exposur	pval.exposu
Type 1 diak	Type 1 diak	Type 1 diak	TRUE	igd	7617096	10	0.1653	3.85E-06
Type 1 diak	Type 1 diak	Type 1 diak	TRUE	igd	97949762	11	0.4435	7.31E-06
Type 1 diak	Type 1 diak	Type 1 diak	TRUE	igd	2.03E+08	2	0.3181	9.37E-06
Type 1 diak	Type 1 diak	Type 1 diak	TRUE	igd	1.4E+08	6	0.5649	2.42E-06
Type 1 diak	Type 1 diak	Type 1 diak	TRUE	igd	1.12E+08	1	0.3108	4.21E-06
Type 1 diak	Type 1 diak	Type 1 diak	TRUE	igd	21468629	12	0.3281	1.13E-06
Type 1 diak	Type 1 diak	Type 1 diak	TRUE	igd	1.11E+08	7	0.2007	4.51E-06
Type 1 diak	Type 1 diak	Type 1 diak	TRUE	igd	43996933	17	0.2624	7.84E-06
Type 1 diak	Type 1 diak	Type 1 diak	TRUE	igd	48671322	4	0.2362	6.06E-06
Type 1 diak	Type 1 diak	Type 1 diak	TRUE	igd	1.81E+08	5	0.243	1.3E-06
Type 1 diak	Type 1 diak	Type 1 diak	TRUE	igd	54437578	6	0.3755	6.4E-06
Type 1 diak	Type 1 diak	Type 1 diak	TRUE	igd	1.43E+08	3	0.1451	8.96E-06

samplesize	id.exposur	exposure	mr_keep.e	pval_origin	data_sourc	action	mr_keep	reliability.e
162964	finn-b-I9_H	|| id:finn-b	TRUE	reported	igd	2	TRUE	high
162964	finn-b-I9_H	|| id:finn-b	TRUE	reported	igd	2	TRUE	high
162964	finn-b-I9_H	|| id:finn-b	TRUE	reported	igd	2	TRUE	high
162964	finn-b-I9_H	|| id:finn-b	TRUE	reported	igd	2	TRUE	high
162964	finn-b-I9_H	|| id:finn-b	TRUE	reported	igd	2	TRUE	high
162964	finn-b-I9_H	|| id:finn-b	TRUE	reported	igd	2	TRUE	high
162964	finn-b-I9_H	|| id:finn-b	TRUE	reported	igd	2	TRUE	high
162964	finn-b-I9_H	|| id:finn-b	TRUE	reported	igd	2	TRUE	high
162964	finn-b-I9_H	|| id:finn-b	TRUE	reported	igd	2	TRUE	high
162964	finn-b-I9_H	|| id:finn-b	TRUE	reported	igd	2	TRUE	high
162964	finn-b-I9_H	|| id:finn-b	TRUE	reported	igd	2	TRUE	high
162964	finn-b-I9_H	|| id:finn-b	TRUE	reported	igd	2	TRUE	high

R2	F
0.000131	21.33933
0.000123	20.10694
0.00012	19.63349
0.000136	22.22922
0.00013	21.16921
0.000145	23.6885
0.000129	21.03981
0.000123	19.9797
0.000126	20.47518
0.000144	23.42053
0.000125	20.36376
0.000121	19.73515
