# Supplementary figures and images for: Exploring the Causal Link Between Autoimmune Diseases and Pulmonary Arterial Hypertension: A Bidirectional Mendelian Randomization Study
Source: Glob Heart. 2025 Jul 4;20(1):58. doi: 10.5334/gh.1445 (PMC12227091; doi:10.5334/gh.1445)

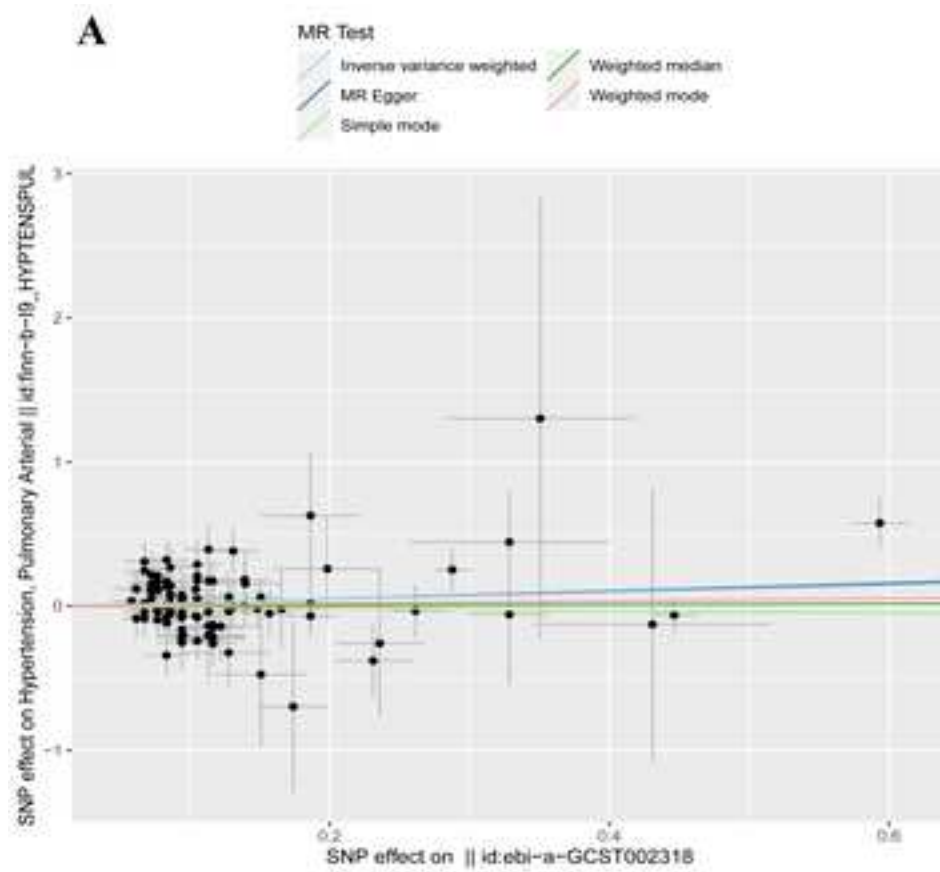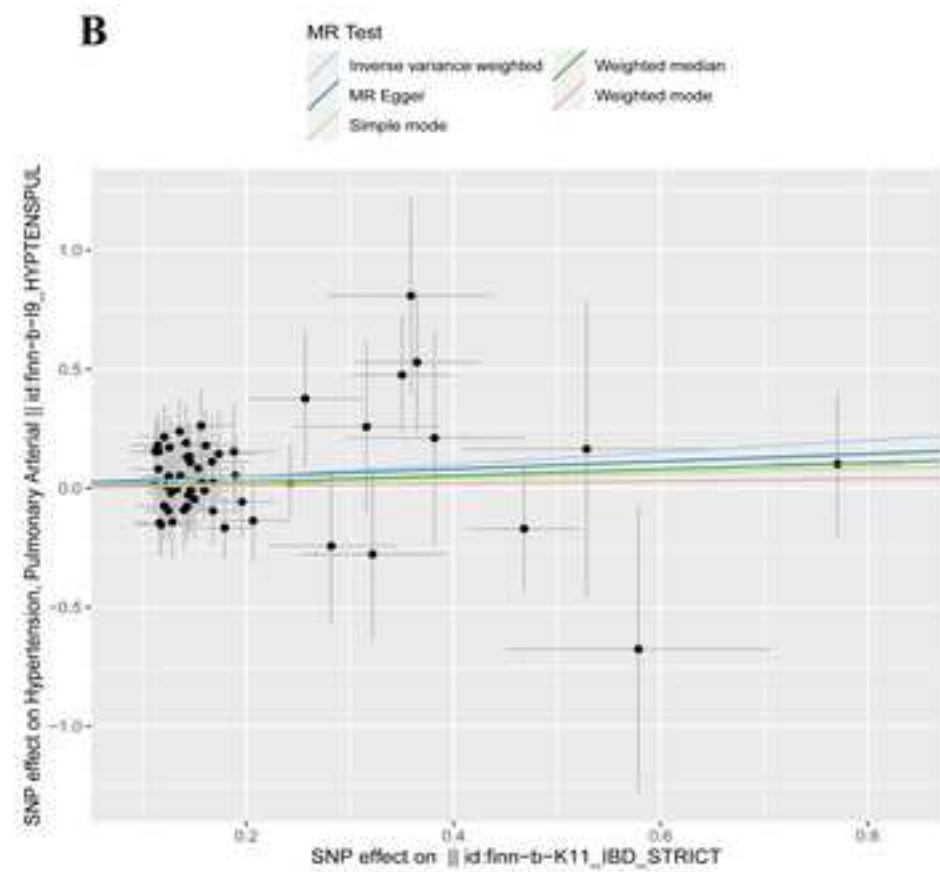

Supplement: Supplementary Figure 1. — Mendelian randomization analysis of autoimmune diseases and the risk of PAH. (A)RA to PAH; (B) IBD to PAH. [file gh-20-1-1445-s2.pdf]

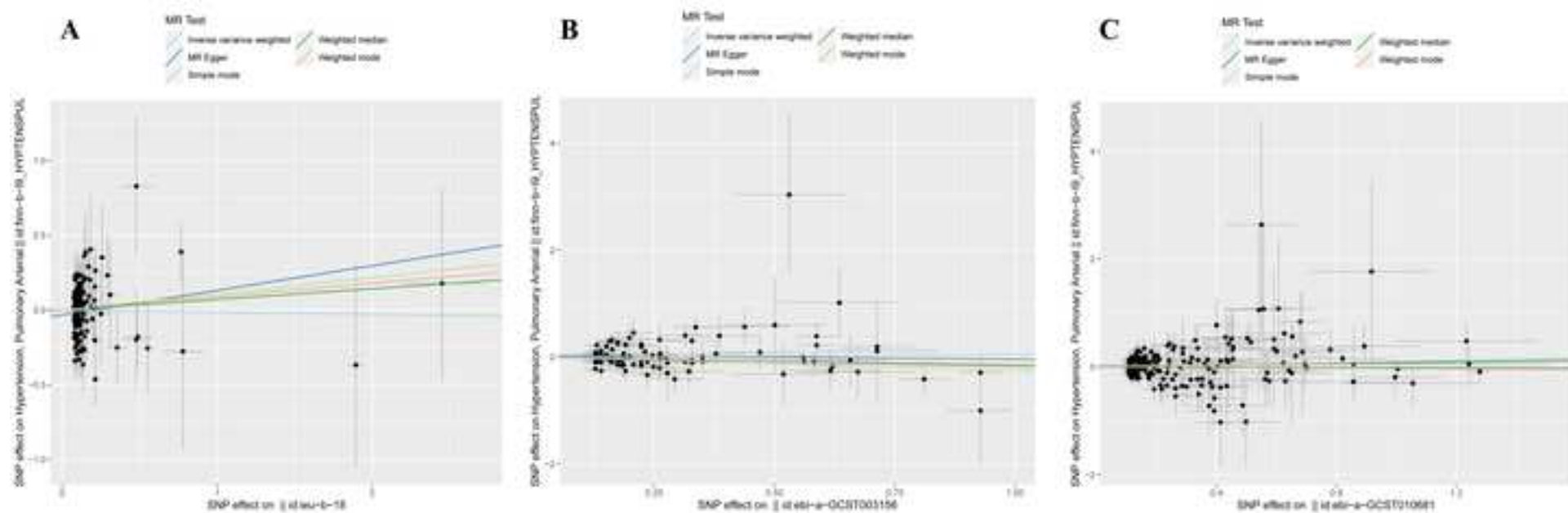

Supplement: Supplementary Figure 2. — Mendelian randomization analysis of autoimmune diseases and the risk of PAH. (A) MS to PAH; (B)SLE to PAH; (C)T1D to PAH. [file gh-20-1-1445-s3.pdf]

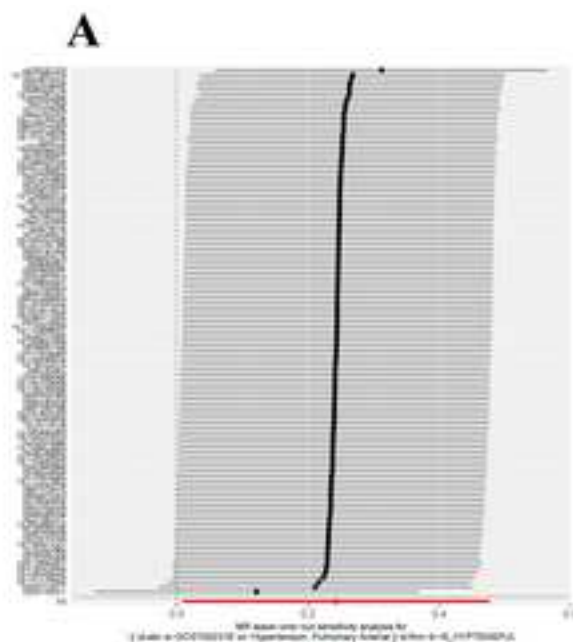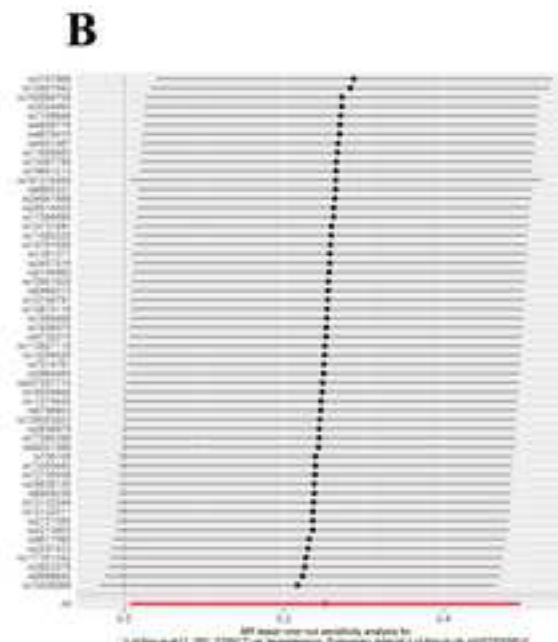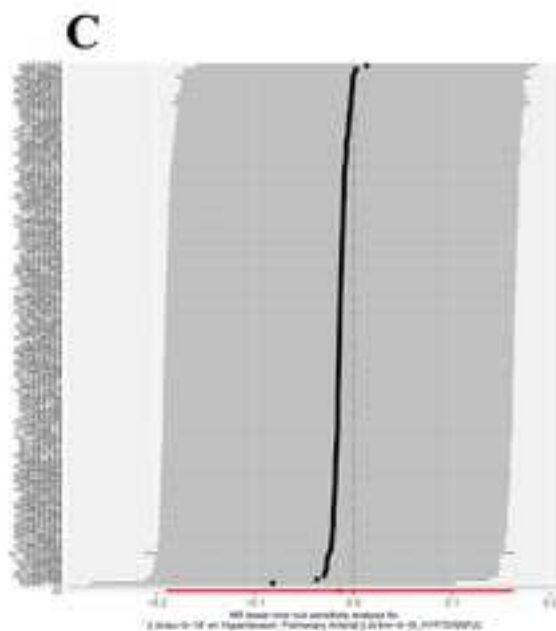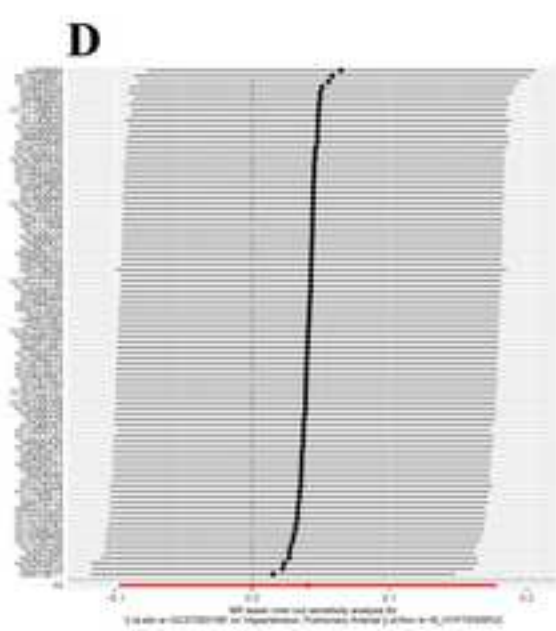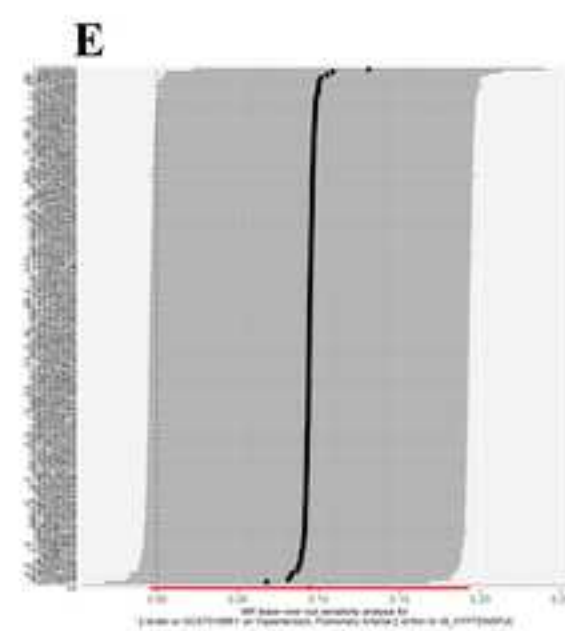

Supplement: Supplementary Figure 3. — The MR “leave-one-out” sensitivity analysis of five autoimmune diseases and PAH. (A)RA to PAH; (B) IBD to PAH; (C) MS to PAH; (D)SLE to PAH; (E)T1D to PAH. [file gh-20-1-1445-s4.pdf]

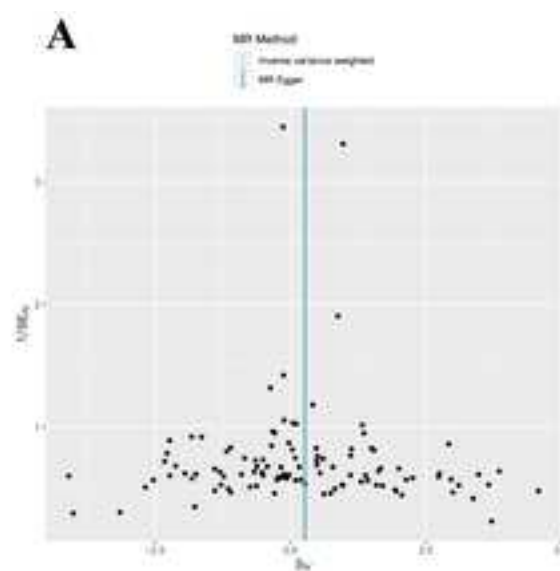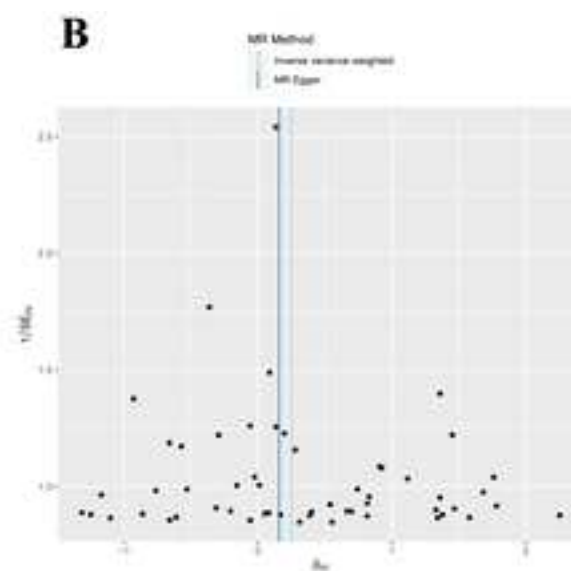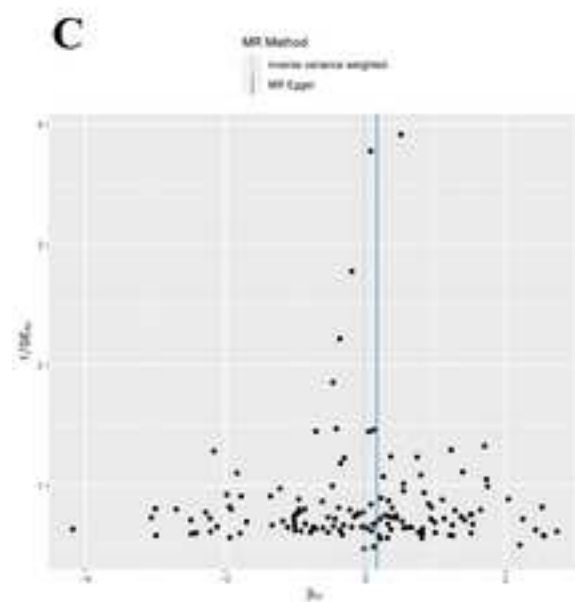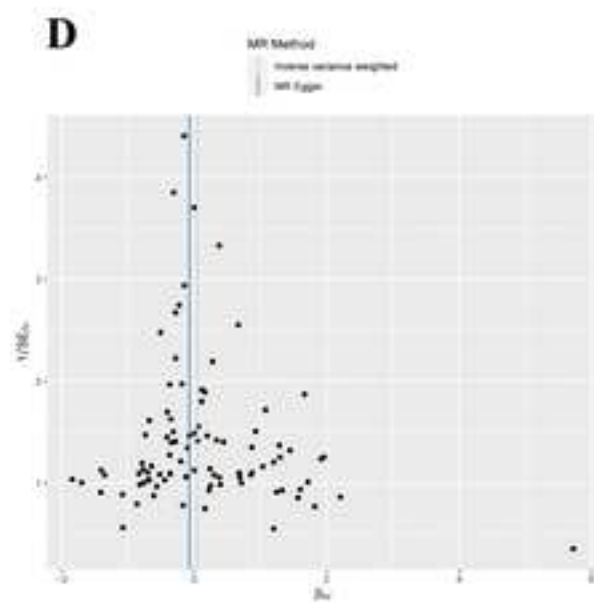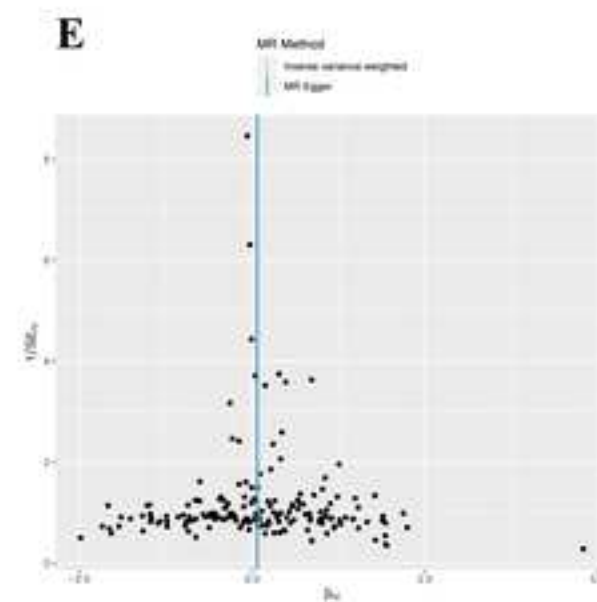

Supplement: Supplementary Figure 4. — Funnel plots of five autoimmune diseases with PAH. The X-axis represents odds ratio (OR), and the Y-axis represents standard error (SE). (A)RA to PAH; (B) IBD to PAH; (C) MS to PAH; (D)SLE to PAH; (E)T1D to PAH. [file gh-20-1-1445-s5.pdf]

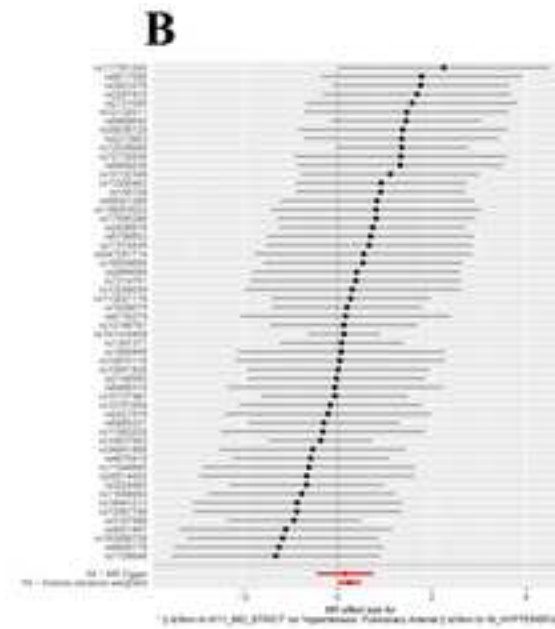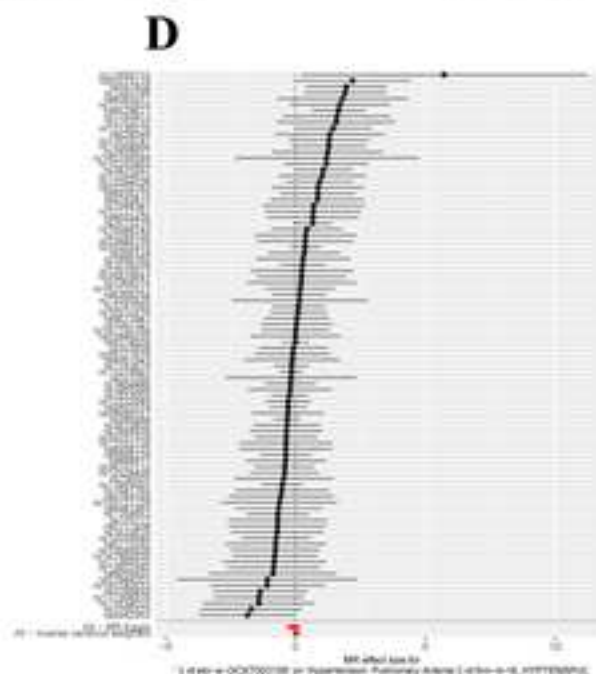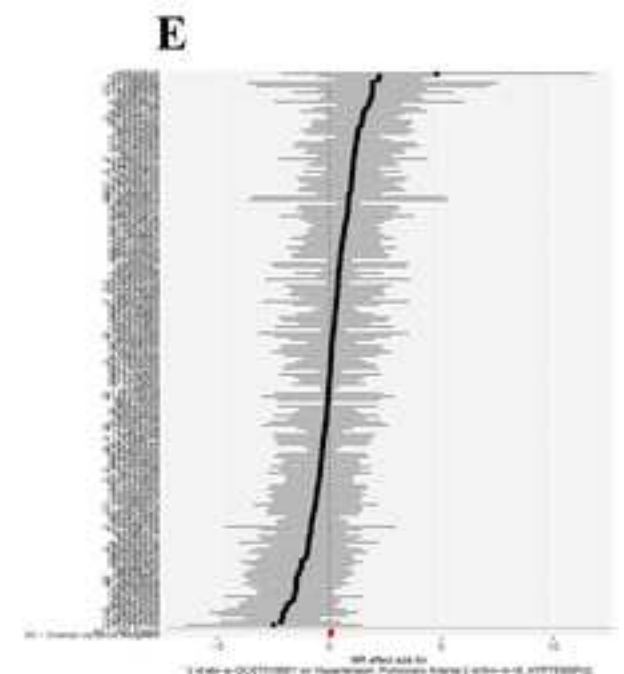

Supplement: Supplementary Figure 5. — Forest plots of five autoimmune diseases with PAH. (A)RA to PAH; (B) IBD to PAH; (C) MS to PAH; (D)SLE to PAH; (E)T1D to PAH. [file gh-20-1-1445-s6.pdf]
